# The SjD Map: an interactive pathway tour into Sjögren’s disease signalling mechanisms

**DOI:** 10.1038/s41540-026-00670-x

**Published:** 2026-03-28

**Authors:** Sacha E Silva-Saffar, Xavier Mariette, Jacques-Eric Gottenberg, Michele Bombardieri, Divi Cornec, Marta E. Alarcon-Riquelme, Michael R. Barnes, Sandra Ng, Wan-Fai Ng, Gaetane Nocturne, Anna Niarakis

**Affiliations:** 1 GenHotel – European Research Laboratory for Rheumatoid Arthritis, Univ. Evry, Univ. Paris-Saclay, Evry, France; 2 Immune Diseases, Microbiology and Innovative Therapies (IDMIT/UMRS1184), Université Paris-Saclay, Inserm, CEA, Fontenay-aux-Roses & Le Kremlin-Bicêtre, France; 3 Service de Rhumatologie, Hôpitaux Universitaires de Strasbourg, Strasbourg, France; 4 Centre for Experimental Medicine and Rheumatology, William Harvey Research Institute, Queen Mary University of London and Barts NIHR BRC & NHS Trust & National Institute for Health and Care Research (NIHR) Barts Biomedical Research Centre (BRC), London, UK; 5 LBAI, UMR1227, Univ Brest, Inserm, CHU Brest, Brest, France; 6 Department of Functional Genomics, Center for Genomics and Oncological Research (GENYO): Pfizer/University of Granada/Andalusian Government, Granada, Spain; 7 William Harvey Research Institute, Queen Mary University of London, London, UK; 8 Translational and Clinical Research Institute, Newcastle University Faculty of Medical Sciences, Newcastle upon Tyne, UK; 9 Molecular, Cellular and Developmental Biology Unit (MCD), Centre for Integrative Biology (CBI), University of Toulouse, UPS, CNRS, Toulouse, France; 10 Lifeware Group, Inria, Saclay-île de France, Palaiseau, France

**Keywords:** Computational biology and bioinformatics, Diseases, Genetics, Immunology

## Abstract

Sjögren’s disease (SjD) remains a major unmet medical challenge, characterised by biological complexity, patient heterogeneity, and the absence of curative treatments. To advance mechanistic understanding and support therapeutic discovery, we developed a comprehensive Molecular Interaction Map (MIM). Differential expression analyses were conducted on peripheral blood samples from SjD patients and healthy controls across three datasets (GSE51092, UKPSSR, PRECISESADS), identifying 1,625 differentially expressed genes (DEGs), of which 25 were shared across all datasets. Nine common DEGs were linked to interferon signalling, reinforcing its pivotal role in SjD pathogenesis. Pathway enrichment analysis revealed 137 pathways, 43 of which were integrated into the MIM alongside literature-derived knowledge. The resulting SjD Map, freely available at https://sjdmap.elixir-luxembourg.org/, encompasses 829 molecular entities connected by 598 interactions by transcriptomic data and by curated evidence. This first comprehensive SjD Map provides an integrative framework for visualising pathways, overlaying omics data, and exploring therapeutic opportunities.

## Introduction

Sjögren’s Disease (SjD) is a prototypic systemic autoimmune disorder that significantly impairs patients’ quality of life. This chronic condition affects 0.05–0.1% of the population, with a striking sex disparity: women are 9–20 times more likely to develop the disease^1,2^. Patients primarily experience a triad of symptoms: musculo-articular pain, dryness and fatigue. In addition, 30–50% of the patients develop systemic complications, with 5–10% of them developing lymphoma, the most severe complication^3^. No treatment has been approved until now in SjD^4^.

In recent years, systems biology and network medicine have emerged as powerful approaches to reduce biological complexity and extract meaningful insights from large-scale datasets. Traditional statistical methods, such as Gene Set Enrichment Analysis (GSEA) and Overrepresentation Analysis (ORA), are widely used to infer biological significance from differentially expressed genes (DEGs), typically by comparing them to curated pathway databases^5^. Resources such as KEGG^6^, Wikipathways^7^, Reactome^8^, and pantherDB^9^ have played a central role in enabling pathway-level interpretation of omics data, offering mechanistic detail and visual context.

Such bioinformatics approaches have already been applied to SjD to decipher its underlying pathogenesis, identify biomarkers, and suggest potential drug targets. However, most of these studies were limited in scope, primarily focusing on differential expression, pathway enrichment, and protein-protein interactions analysis, without addressing the broader mechanistic understanding of the disease ^10–13^.

To address the limitations of conventional approaches, systems biology has increasingly embraced integrative methods that combine heterogeneous data sources, including literature-based knowledge and high-throughput experimental data, within formalised, standardised frameworks. This holistic strategy has led to the development of several “disease map” projects^14^, centred around Molecular Interaction Maps (MIMs) that represent disease mechanisms as a whole. Notable examples include the Covid-19 map^15^, the Parkinson’s map^16^, the Atlas of Cancer Signalling Network^17^, and, more recently, the Rheumatoid Arthritis Atlas^18,19^. These initiatives facilitate a deeper understanding of disease pathogenesis and provide a structured platform for generating therapeutic hypotheses.

Such maps serve as disease-specific knowledge repositories that are both human- and machine-readable, enabling comprehensive exploration and integration of molecular mechanisms. To ensure interoperability, reproducibility, and standardisation, these resources are often developed using the Systems Biology Graphical Notation (SBGN) standard^20^, which formalises the representation and exchange of biological networks across computational tools and research groups.

In this study, we aimed to build the first fully standardised MIM for SjD, properly curated and annotated, available at https://sjdmap.elixir-luxembourg.org/ via a web hosting platform to allow for easy navigation and significant zoom-in capabilities. The SjD map, hosted on the MINERVA platform, ensures accessibility to all dedicated Sjogren-specific content for all users^21^. It offers an integrative framework that incorporates mechanistic insights from peer-reviewed literature, transcriptomic datasets, and relevant clinical information. Its primary aim is to bridge the existing knowledge gap between disciplines and facilitate cross-domain exploration of SjD pathogenesis. In this work, we highlighted the different uses and applications of MIMs, enabling both the visualisation of pathways involved in specific manifestations of SjD (e.g., lymphoma) and the identification of relevant biomarkers, thereby paving the way for future targeted therapeutic strategies.

## Results

### Identification of signalling pathways implicated in Sjögren’s disease pathogenesis via a data-driven approach

**Fig. 1:**
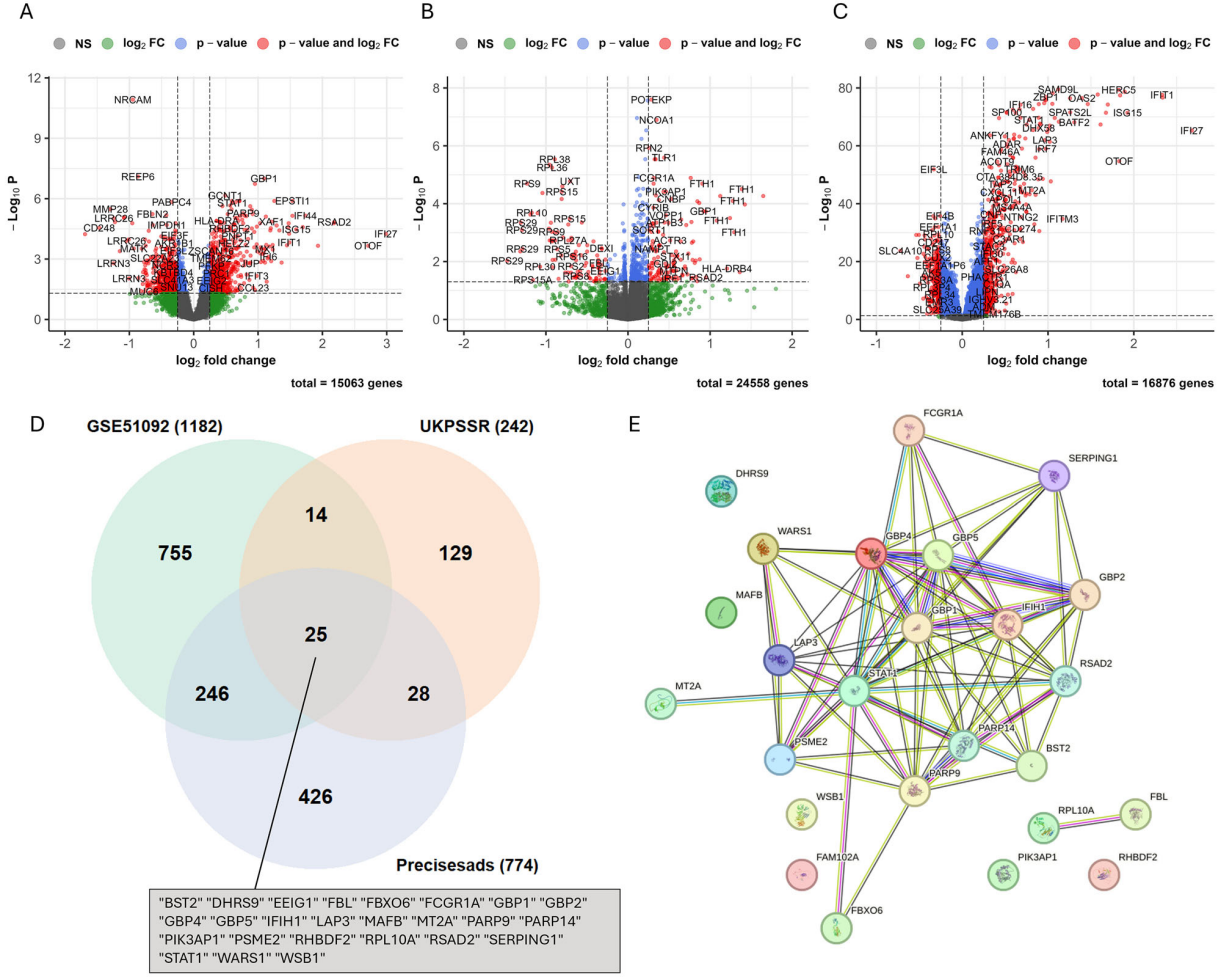
Gene expression changes in SjD. Volcano plots showing in **A** DEGs of SjD patients vs. controls in GSE51092 (microarray data including 190 SjD patients and 32 controls), **B** DEGs of SjD patients vs. controls in UKPSSR (microarray data including 151 SjD patients and 29 controls), **C** DEGs of SjD patients vs. controls in PRECISESADS (RNA-Seq data including 304 SjD patients and 341 controls), **D** Venn diagram showing the common DEGs of the three comparisons mentioned above. **E** Network Analysis of the 25 intersected genes was performed on STRINGDB^29^.

In the first step of our workflow, based on analysis of three blood transcriptomic datasets, we identified 1,162 DEGs in GSE51092, 239 in UKPSSR, and 774 in PRECISESADS (Supplementary Data S2). The volcano plots in Fig. 1 illustrate gene dispersion, showing that although the range of log fold changes (logFC) is similar across studies (approximately -2 to 4), the datasets differ in statistical significance. GSE51092 and UKPSSR, both microarray-based datasets, exhibit comparable maximum adjusted p-values (around 10^-10), whereas the RNA-seq dataset PRECISESADS shows much higher significance, with adjusted p-values approaching 10^-80. This greater statistical power can be attributed to the more advanced transcriptomic technology (RNA-seq) and the larger cohort size in the PRECISESADS study (see Methods).

Regarding expression patterns, GSE51092 identified 609 upregulated and 553 downregulated genes, UKPSSR identified 140 upregulated and 99 downregulated genes, and PRECISESADS identified 526 upregulated and 248 downregulated genes. A comparative analysis of the three datasets identified 25 genes commonly upregulated across all studies (Fig. 1D). Most of these genes, located in the upper-right quadrant of the volcano plots (Fig. 1A–C), are interferon-related, including STAT1 and interferon-stimulated genes (ISGs) such as *IRF7, IFI44*, and *IFIT1*. A network analysis using STRINGdb showed significant interactions among 17 of the 25 intersecting genes, further emphasising the involvement of interferon (IFN) signalling in these datasets (Fig. 1E).

**Fig. 2:**
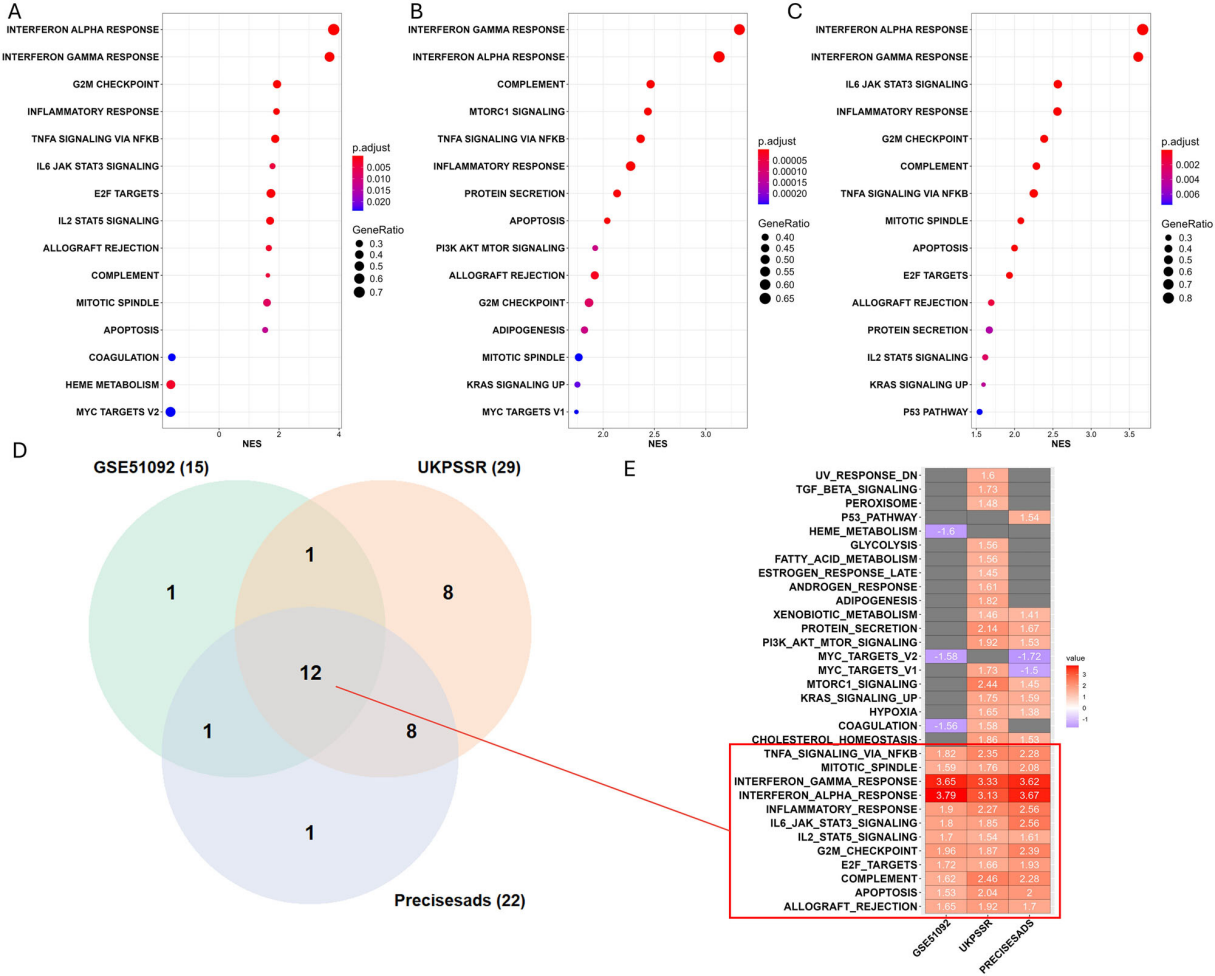
Enrichment analyses of sjögren blood transcriptome. Dotplots showing in **A** Enrichment GSEA of GSE51092 (microarray data including 190 SjD patients and 32 controls), **B** Enrichment GSEA of UKPSSR (microarray data including 151 SjD patients and 29 controls), **C** Enrichment GSEA of PRECISESADS (RNA-Seq data including 304 SjD patients and 341 controls), **D** Venn diagram showing the common enriched pathways, **E** Heatmap displaying normalised enrichment scores of the pathways enriched. The red box indicates the common enriched pathways across all three datasets.

Following the DEA, pathway enrichment analysis was conducted using two statistical techniques: GSEA and ORA. The GSEA identified 15, 29, and 22 enriched pathways for GSE51092, UKPSSR, and PRECISESADS, respectively. The Top 15 enriched pathways in GSEA are shown in Fig. 2A–C, which display a significant increase in pathways related to inflammation. The first two enriched pathways that are upregulated and common to all datasets are the IFN alpha and IFN gamma pathways, with a GeneRatio of around 0.6, corresponding to 60% of the common enriched genes between our study and the reference dataset. In the Venn Diagram in Fig. 2D, we observed 12 enriched pathways common to all datasets with a positive normalised enrichment score. Apart from the Mitotic Spindle, G2M checkpoint, E2F targets, and Apoptosis, the remaining eight pathways are all related to inflammation (Fig. 2E).

The ORA, using the Reactome knowledge base and the cumulative DEGs from SjD blood transcriptomic data, identified 137 enriched pathways. Of these, 43 pathways were retained based on mechanistic relevance, supported by expert validation and alignment with GSEA results (See Methods). The top 10 pathways of this list are presented in Table 1, while the remaining pathways, along with the Reactome report, are provided as Supplementary Data S3 and S4. The pathways highlight immune system-related processes, emphasising the pivotal role of immune cells in the pathogenesis of SjD. In particular, the analysis indicates a significant involvement of the type I and type II IFN pathways, with nearly half of the entities represented in each case, as well as signalling mechanisms related to macrophages and B cells. These 43 statistically and biologically significant mechanistic pathways were subsequently utilised to construct and enrich the SjD Map.

**Table 1:** TOP10 overrepresented and curated pathways on Reactome

Pathway identifier	Pathway name	#Entities found	#Entities total	Entities FDR
R-HSA-877300	Interferon gamma signalling	87	177	1.35E-14
R-HSA-909733	Interferon alpha/beta signalling	82	129	1.35E-14
R-HSA-198933	Immunoregulatory interactions between a Lymphoid and a non-Lymphoid cell	83	249	1.99E-12
R-HSA-2029481	FCGR activation	44	103	1.30E-09
R-HSA-2029485	Role of phospholipids in phagocytosis	48	129	1.30E-08
R-HSA-5690714	CD22 mediated BCR regulation	33	72	5.46E-08
R-HSA-2730905	Role of LAT2/NTAL/LAB on calcium mobilisation	41	107	8.98E-08
R-HSA-2029482	Regulation of actin dynamics for phagocytic cup formation	52	158	1.09E-07
R-HSA-2871809	FCERI mediated Ca+2 mobilisation	44	129	5.38E-07
R-HSA-2029480	Fcgamma receptor (FCGR) dependent phagocytosis	56	193	1.45E-06

### Assembly of the identified signalling pathways into the SjD Map

**Fig. 3:**
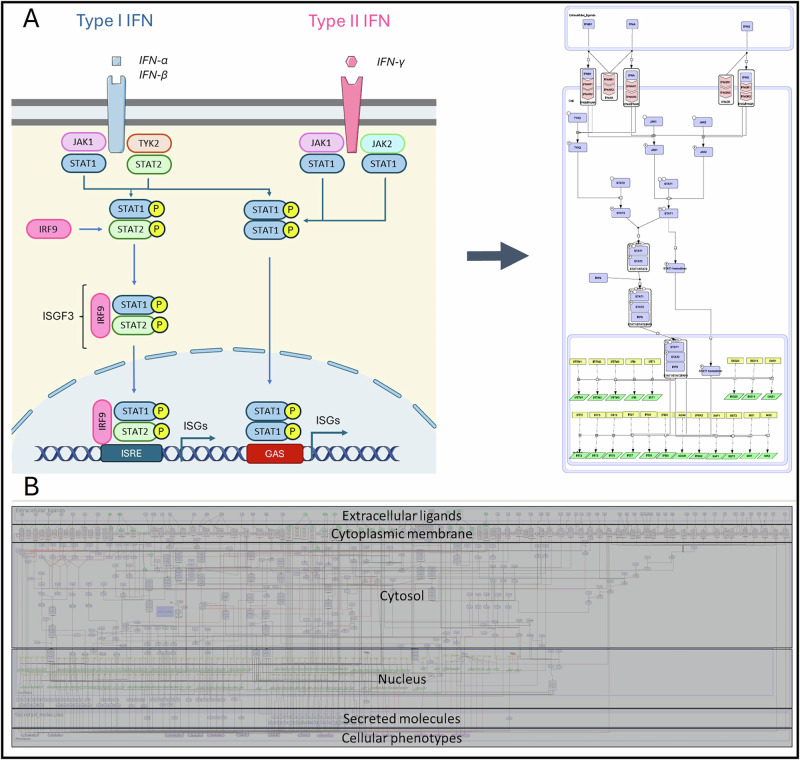
Map building and visualisation. **A** Representation of the IFN signalling pathway in classical scientific articles (left) vs. its depiction in a standardised disease map format (right). This figure was drawn with images from Servier Medical Art (Servier). **B** Overview of the SjD Map. The map’s layout follows the organisation of a cell, from extracellular ligands to distinct cellular phenotypes.

The SjD map depicts hallmark signalling pathways, gene regulation, molecular mechanisms and phenotypes involved in SjD pathogenesis. The MIM is constructed in CellDesigner, allowing the creation of a formalised, standardised map with detailed mechanistic insights into the biological reactions involved (See ‘Methods’). The SjD map is compartmentalised top-to-bottom to represent the flow of information from the extracellular space (ligands) to the secreted compartments and the cellular phenotype section. Figure 3A compares the conventional representation of the IFN signalling pathway commonly found in research articles with its standardised depiction using SBGN-PD within a MIM. The use of SBGN-PD enhances understanding of the biological processes undergone by individual molecular species. The signalling cascades start after ligand receptor connection, go through the plasma membrane, into the cytosol, to the nucleus (gene regulation) and end up in the secreted compartment (Fig. 3B).

The SjD map is based on transcriptomic analysis of SjD patient cohorts, an exhaustive literature search, and manual pathway curation and data mining. During pathway assembly, experts from SjD were consulted on data integration and pathway representation to ensure accurate interpretation of experimental evidence and to reflect scientific consensus on disease pathogenesis. The map benefited from several exhaustive reviews and constructive feedback from experts in the Necessity consortium. This thorough revision in every step of its construction led to scrutiny of the depicted mechanisms, both in terms of content and form. The SjD map comprises 829 molecular species interconnected by 598 interactions. The biological entities include 403 proteins, 123 complexes, 133 RNAs, 2 Antisense RNAs, 134 genes, two ions, and 15 simple molecules, such as lipopolysaccharides (LPS). The proteins encompass extracellular, membrane, and cytoplasmic proteins, including signalling intermediates, enzymes, and transcription factors. The 598 interactions between species represent various biological processes, including state transitions, catalysis, inhibition, transport, heterodimer formation, and physical stimulation.

The map contains hallmark cellular and molecular pathways involved in SjD pathogenesis, including type I and type II IFN signalling (IFN-α and IFN-γ) and a broad array of interleukin-mediated cascades (IL-2, IL-6, IL-7, IL-12, IL-15, IL-21), which primarily converge on the JAK-STAT pathway. Additionally, both canonical and non-canonical NF-κB pathways (activated by BAFF, APRIL, BCR, and CD40) are represented, along with Toll-like receptor (TLR) signalling and diverse chemokine axes (CXCL/CCL).

Finally, these signalling cascades converge to drive specific cellular outcomes, informed by both literature and pathway databases, and categorised into fourteen distinct phenotypes: MHC Class I Activation, MHC Class II Activation, T Cell Activation/Differentiation, B Cell Activation/Survival, Cell Proliferation/Survival, Inflammation, Chemotaxis/Infiltration, Angiogenesis, Lymphoid Organ Development, Apoptosis, Regulated Necrosis, Matrix Degradation, Fibrosis, and Phagocytosis. These phenotypes represent the end points of several convergent pathways and grouped biomarkers and can be viewed as cell-fate decisions.

### Validity of the SjD map

**Fig. 4:**
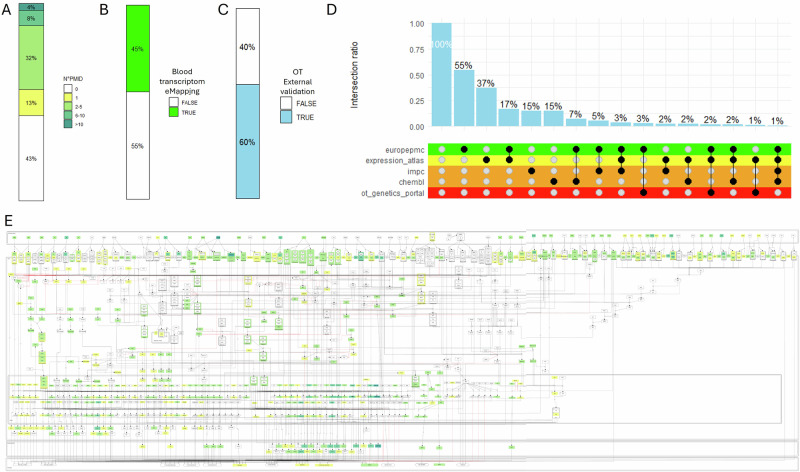
SjD Map annotation score and external validation. Barplots representing **A** the quantification of literature data. The number of publications associated with an entity, by categories in white, yellow, light green, and dark green, respectively, corresponding to 0, 1-5, 6-10, and more than 11 PMIDs. Literature sources are fully referenced with PMIDs and were integrated following the procedure described in Methods (covering publications from 2010 onward); **B** the quantification of SjD blood transcriptome. The transcriptomic datasets and their corresponding dates are as follows: GSE51092 (2013), UKPSSR (2011), and PRECISESADS (2015); and **C** Barplot representing the Coverage of Sjögren OpenTargets database information on the SjD Map. **D** Upset plot representing the primary sources retrieved in OpenTargets as an external validation database and their intersections. Each category is colour-coded consistently with the OpenTargets_Validation overlay available on the SjD map. **E** Overlay of Literature data on the SjD map. Each component is coloured according to its annotation.

The confidence in the relevance of a biological entity in SjD pathogenesis increases with the number of supporting publications or gene expression data linked to that entity. The SjD map incorporates 216 PubMed references, distributed across the map and accounting for 57% of its content (Fig. 4A, E). Of the biological entities, 13% are associated with a single PubMed reference, while 32% are linked to two to five references. Furthermore, 45% of the map is covered by blood transcriptomic data from Sjögren’s patients, which was used to construct it (Fig. 4B). However, some components in the map lack direct associations with published studies or enrichment from the blood dataset. In many cases, these are small, simple molecules such as Phosphatidylinositol-3,4,5-Trisphosphate (PI-^3–5^-P3) or Lipopolysaccharide (LPS), which are less frequently studied in SjD pathology than their receptors. Other unannotated components function as intermediates, included to ensure the complete visualisation of signalling pathways, offering a more comprehensive representation of the disease mechanism, and enhancing the connectivity of the underlying process description diagram.

To assess the relevance of the SjD map, we also used the OpenTargets database, which integrates data from various sources, including literature, genetic variants, and gene expression related to SjD. We found that 60% of the map was covered by this external database (Fig. 4C). Most of the matching content was derived from literature data (Europe PMC), accounting for 55%, followed by gene expression data (Expression Atlas) at 37%, with a 17% overlap between the two (Fig. 4D).

The important coverage indicates that our SjD map successfully captures the most relevant molecular entities associated with the disease, with the added value of the mechanistic representation.

### Network analysis

**Fig. 5:**
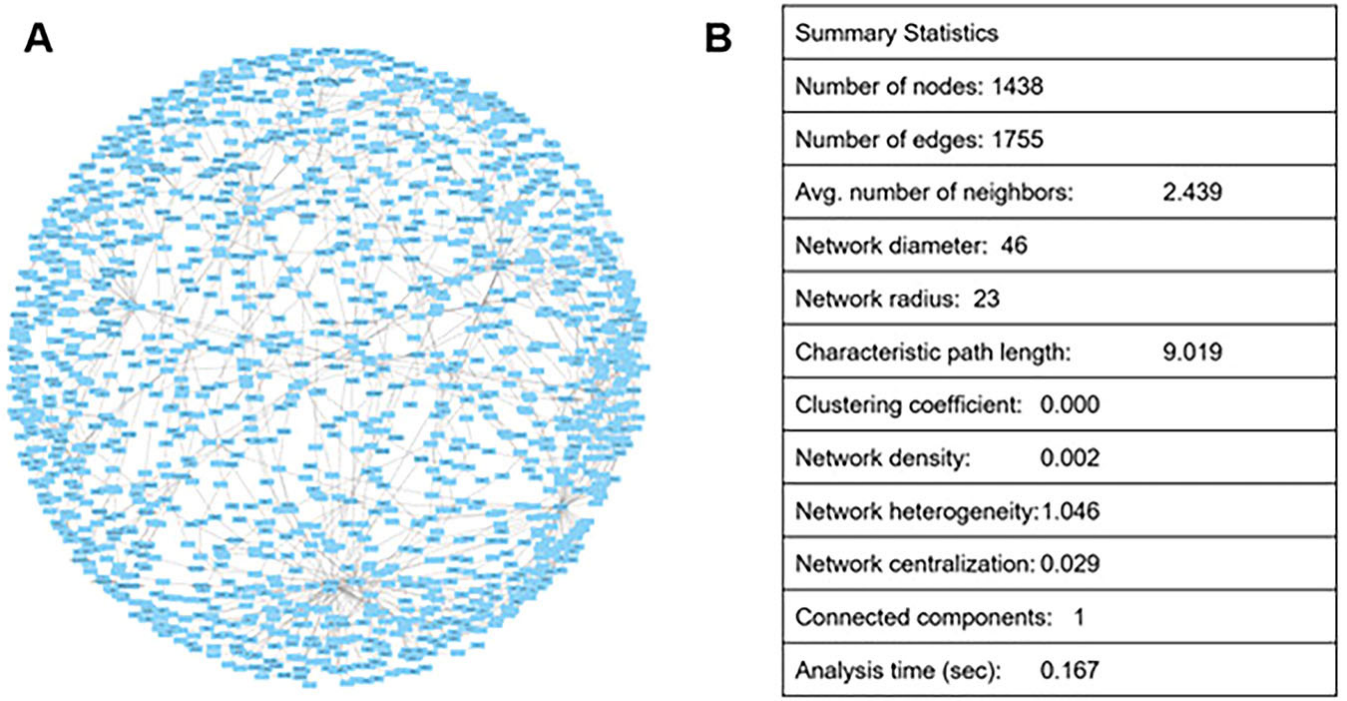
Visualisation and analysis of the SjD map as a complex network. Import of the SjD map in Cytoscape after MINERVA export **A** and analysis with NetworkAnalyzer **B**.

The SjD map can also be viewed as a complex graph and, as such, can be analysed in terms of its structure. We followed two different approaches. The first topological analysis of the SjD Map was performed by importing the network directly from the MINERVA-generated XML file into the software Cytoscape^22^, using the built-in *Network Analyzer* tool. This analysis revealed a network composed of 1438 nodes and 1755 edges. The relatively low average number of neighbours (2.44) and very low network density (0.002) indicate a sparse topology, consistent with a highly modular biological network in which most entities participate in localised interactions rather than forming a highly interconnected system. The network diameter (46) and characteristic path length (9.0) suggest that signal propagation across the map may require multiple intermediate steps, reflecting both the biological complexity of Sjögren’s Disease mechanisms and the inclusion of reactions as nodes in the imported structure. The clustering coefficient (0.000) supports the absence of dense subnetworks or cliques, while the low centralisation (0.029) and heterogeneity (1.046) indicate that no single node dominates the overall network organisation (Fig. 5).

**Fig. 6:**
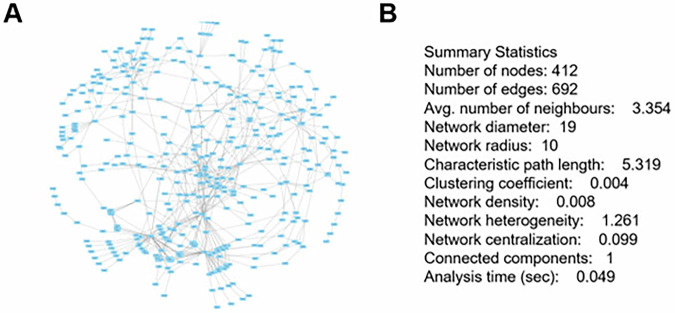
Visualisation and analysis of the SjD map as an Activity Flow network. Import of the SjD map in Cytoscape after CaSQ transformation to Activity Flow **A** and analysis with NetworkAnalyzer **B**.

The second topological analysis was performed on a CaSQ-reduced version of the map^23^, which simplifies the network by aggregating reactions, before analysing the network in Cytoscape (Fig. 6). When comparing the first analysis (1438 nodes, 1755 edges) with the second (412 nodes, 692 edges), an apparent increase in network compactness is observed. The average number of neighbours rises from 2.44 to 3.35, and the network density increases from 0.002 to 0.008, indicating a greater proportion of connections among the remaining nodes. Consistently, the characteristic path length decreases from 9.02 to 5.32, and the network diameter decreases from 46 to 19, reflecting a more cohesive, more efficiently connected structure. The slight increases in clustering coefficient (0.000 → 0.004) and centralisation (0.029 → 0.099) suggest a shift toward a more hierarchically organised topology, where specific key nodes play more prominent coordinating roles.

In both analyses, the top five hub nodes remained stable: *Inflammation*, *STAT1 homodimer*, *STAT1/STAT2/IRF9*, *RELA/NFKB1*, and *Chemotaxis/Infiltration*, emphasising the core inflammatory signature of the SjD Map. These hubs highlight the central role of interferon (JAK–STAT) and NF-κB signalling pathways in the molecular architecture of SjD.

### Applications

The SjD map is structured to support the integrative analysis of SjD experimental and clinical data through its standardised, mechanistic molecular representation.

A key feature of the map is its capacity to contextualise transcriptomic data by allowing differential expression overlays directly onto the molecular pathways, providing a systems-level view of pathway regulation.

**Fig. 7:**
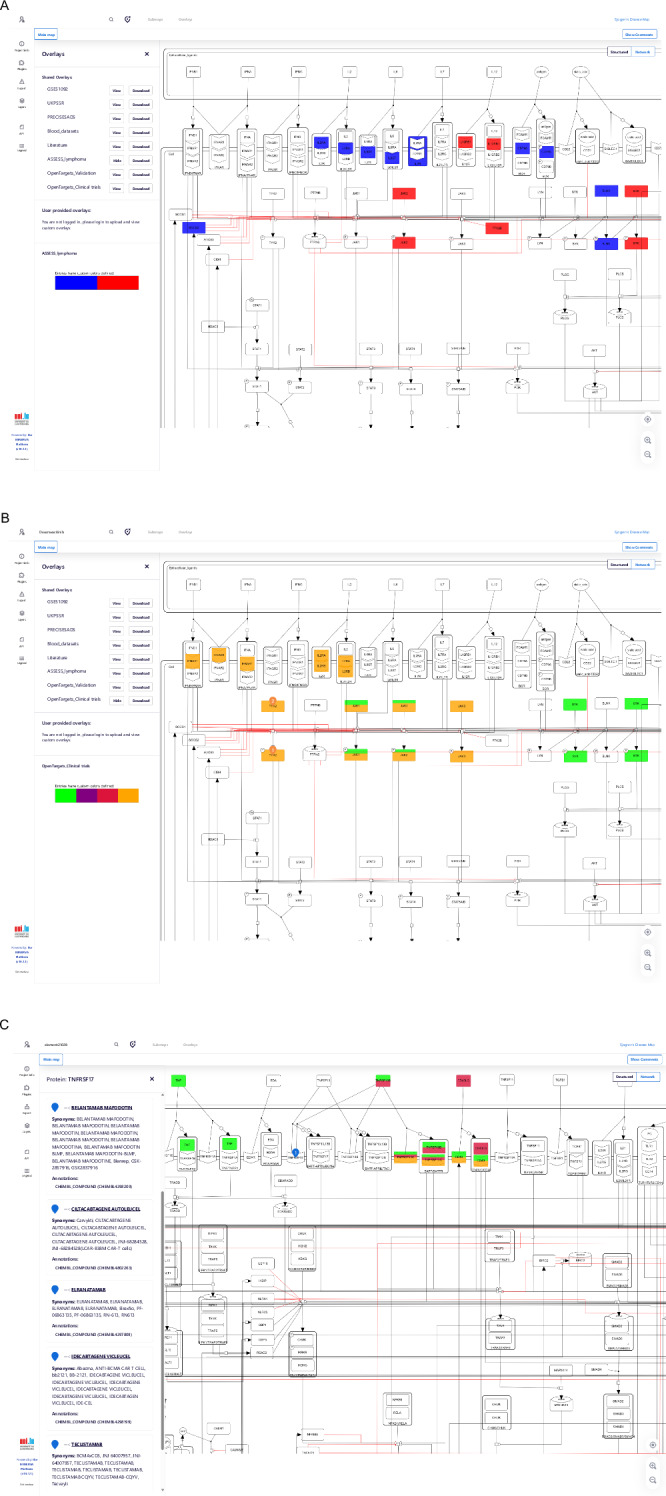
Functionality of the SjD map. **A** ASSESS lymphoma Differential Expression analysis overlay; Overexpressed species in red, underexpressed species in blue. **B** Drug targets search and overlay for SjD with different colours for the state of the clinical trial: Completed in Green, Not yet recruiting/Active not recruiting/Unknown status in Purple, Withdrawn or terminated in Red, Recruiting in orange; **C** Results of the search for Drug compounds targeting TNFSF17 (BCMA).

As an example of application shown in Fig. 7A, we projected the DEGs between SjD patients who developed lymphoma and those who did not (See “Methods”) onto the SjD map (DEGs are given in Supplementary Data S5). This overlay notably highlights, for example, the upregulation (in red) of BTK and APRIL (TNFSF13), two genes recently shown to be involved in lymphomagenesis in SjD patients ^24^.

In addition to transcriptomic projections, the SjD map allows users to explore drug-target interactions. For instance, users can visualise biological targets of drugs currently being tested in clinical trials for SjD via a combination of DrugBank^31^ and a specific overlay. In Fig. 7B, we show the result of the research for “Deucravacitinib”, a TYK2-selective JAK inhibitor. The map highlights TYK2 as the molecular target of this drug. The “Clinical trials overlay” feature provides further insights by showing the clinical trial status of specific drug targets. Deucravacitinib is shown in orange, indicating that the trial is still recruiting (ClinicalTrials.gov Identifier: NCT05946941). This Phase III trial, sponsored by Bristol Myers Squibb, was initiated in 2023 and is expected to report efficacy results by the end of 2026. In contrast, terminated or withdrawn trials are marked in red, such as those targeting Lymphotoxin A/B, suggesting that these pathways may have diminished clinical interest, helping prioritise more promising therapeutic avenues for this disease. Components displayed in green are of particular interest because their clinical trials have been completed, and results have already been posted.

Additionally, reverse searches can be performed for drug repurposing. By selecting a protein on the map, users can view all drugs that target it, which could inform future in vitro or in vivo experiments. For instance, to explore therapeutic strategies aimed at B-cell modulation, beyond BAFF and BAFF receptor, which have already been clinically tested, users can select TNFSF17 (also known as BCMA) on the map. This reveals several therapies targeting this receptor in other diseases, including Elranatamab (bispecific antibody), Belantamab mafodotin (antibody-drug conjugate), and Ciltacabtagene autoleucel (CAR-T cell therapy). Given the central role of B cells in SjD pathogenesis, such tools may help identify promising drug repurposing candidates for experimental validation. Finally, the SjD Map available on the MINERVA platform enables users to create custom overlays, facilitating the visualisation of impacted components or pathways in SjD according to their individual research objectives.

## Discussion

This study describes the first MIM dedicated to SjD. The SjD Map was developed using systems biology approaches and a hybrid framework that integrates transcriptomic data analysis and literature curation concomitantly, building upon previously described schemes^18,19^. The map is SjD-specific and can serve as a complementary tool for target identification, mechanistic insights, and drug repurposing, as demonstrated in the selected applications. Moreover, SjD Map allows the visualisation of pathways involved in clinical manifestations of particular interest in SjD, such as lymphomagenesis. As Sjögren’s disease is heterogeneous, understanding its different phenotypes is of utmost importance for patient stratification. The SjD map can also serve as a basis for targeted drug repurposing efforts and for in silico prediction of drug effects at both molecular and phenotypic levels ^25–28^.

The transcriptomic analysis of the three blood sample datasets primarily identified upregulated IFN-related genes in SjD patients. Subsequent enrichment analyses confirmed the IFN signatures and further highlighted inflammatory processes. Our findings align with previous studies, which have consistently identified strong inflammatory signatures, particularly an IFN type 1 and 2 response, across various omics levels in both salivary gland cells and immune cells, reinforcing the central role of IFN pathways in SjD pathophysiology^10–13^. However, despite advances in data analysis, many previous studies fail to integrate mechanistic details and prior knowledge. The SjD Map is an effort to combine omics data, clinical features, and mechanistic representations in an easy-to-use, web-accessible platform.

Existing resources such as STRINGDB^29^ and other PPI databases provide valuable interaction networks, yet they often fail to capture the mechanistic insights underlying biological processes or diseases. In contrast, most existing mechanistic pathway databases often lack either disease specificity, proper annotations, or referenced literature. The SjD Map addresses this gap by incorporating hallmark disease pathways through extensive manual curation, rigorous omics data analysis, and expert validation. As part of the disease map community^14^, the SjD map has been built using standardised methodologies to synthesise and visualise complex biological interactions in a disease-specific context.

Subjecting the map to omics data projection, we analysed potential DEGs driving lymphomagenesis and notably identified BTK and APRIL (TNFSF13), previously shown to be drivers of lymphomagenesis by Duret et al.^24^. The MIM thus provides a valuable tool for visualising the molecular heterogeneity of SjD. For instance, identifying upregulated pathways in patients who develop lymphoma may pave the way for targeted therapeutic strategies or the development of predictive biomarkers for this serious complication, both of which represent significant clinical challenges. Given the broad heterogeneity of the disease, the potential applications are numerous. The MIM could serve as an in-silico pre-screening platform to explore pathophysiological hypotheses related to specific, and sometimes rare, disease complications.

Additionally, overlaying clinical trials on the map allowed us to visualise drugs tested in completed SjD clinical trials, including well-known inhibitors such as filgotinib (JAK3 inhibitor), tirabrutinib (BTK inhibitor), and lanraplenib (SYK inhibitor). While most of these trials yielded adverse outcomes, the case of filgotinib illustrates the map’s potential to support refined therapeutic hypotheses. Although initially unsuccessful, filgotinib was later reconsidered for use in a molecularly defined subgroup of patients exhibiting an IFN signature^30^. Notably, the SjD map enables integration of transcriptomic signatures with clinical trial metadata, allowing users to explore associations between molecular phenotypes and therapeutic responses. These overlays enhance the clinical relevance of the SjD Map, making it useful for both educational purposes and in-depth drug discovery efforts.

SjD involves multiple biological processes and affects various cell types, particularly salivary gland epithelial cells and both blood and infiltrating immune cells. In this study, we conducted a literature curation to enrich the map with both blood and gland-related pathways. However, our transcriptomic analysis focused exclusively on blood datasets, as they are more relevant for identifying biomarkers. Although integrating omic data from salivary gland tissues could enhance the map’s biological relevance, obtaining clean, robust gland datasets remains challenging due to heterogeneity in methods and samples. Addressing this limitation was one of the primary motivations for developing ADEx (https://adex.genyo.es/), a web-based application designed to facilitate the integration and exploration of autoimmune disease datasets^31^. Future studies should incorporate this data to refine the map further.

The SjD Map was designed as a first comprehensive attempt to synthesise hallmark disease pathways into a single mechanistic blueprint. While we acknowledge that the resulting network is complex and that some pathways originate from different cell types, there are several advantages to this integrated approach, such as (a) comprehensive overview: Connecting pathways in a single map provides a clearer picture of the various biological processes involved in Sjögren’s Disease, allowing researchers to appreciate how different mechanisms may intersect and contribute to disease pathogenesis; (b) Identification of synergies: Integrated maps facilitate the study of combined effects, which is particularly relevant when exploring therapeutic interventions that target multiple pathways simultaneously, (c) Enhanced connectivity for modelling: Synthesis of pathways improves network connectivity, which is essential for understanding signal propagation from receptor activation to phenotypic outcomes, and for enabling computational modelling of disease processes; (d) Mechanistic detail and standards compliance: The SjD Map is process-oriented, capturing mechanistic information through biochemical reactions, is fully annotated, and is compliant with standardised formats, making it model-ready for further computational analyses, especially current frameworks dedicated to Boolean modelling ^23^.

Moreover, although the SjD Map is currently designed to represent single-cell context, it can be dissociated to illustrate interactions between multiple cell types, thereby improving its utility for modelling the complex cellular interplay characteristic of SjD. The integrated map serves as a foundational framework that can be refined and contextualised as more data become available, such as single-cell RNA-seq for specific immune or glandular cell types. Inspired by the RA-Atlas^19^, this flexibility allows for a more nuanced understanding of the disease’s pathophysiology, ultimately contributing to more targeted therapeutic approaches. One possible approach would be to structure the map into two or three compartments, corresponding to blood and glandular environments, with an intermediary compartment representing infiltrating immune cells.

Additionally, while the map was manually curated and enriched, a process that is both rigorous and time-intensive, future iterations could benefit from artificial intelligence algorithms capable of extracting biological insights and network relationships from literature, like INDRA^32^ or BioChatter^33^. The integration of large language models may further streamline this process and enhance both the efficiency and depth of map development.

Finally, the MIM currently provides a static framework for studying the immunopathology of SjD. Our goal is to go further by developing a dynamic model capable of performing in silico simulations to predict molecular behaviour in response to defined perturbations, such as pathway activation or modulation by therapeutic agents. This would allow for the assessment of downstream effects on molecular phenotypes. The MIM represents the essential foundation for such dynamic modelling, which could open new avenues for understanding and managing SjD.

## Methods

### Datasets

We sought to use three different whole-blood SjD-specific datasets in our study. We used GSE51092, a publicly available microarray dataset on GEO, containing samples of 190 SjD patients and 32 controls^34^. The series matrix file was downloaded for downstream analysis. The two other datasets were available upon request via the NECESSITY consortium. UKPSSR, a microarray dataset of 151 SjD patients and 29 controls, was sourced from the United Kingdom Primary Sjögren’s Syndrome Registry^35^. PRECISESADS, an RNA-Seq study for 304 SjD patients and 341 controls. More technical details about data collection and processing are available in PRECISESADS’ main article^36^. The availability of data and metadata, as well as the release status of the datasets used in this study, are summarised in Table 2.

**Table 2:** Data and metadata availability, and release status of the datasets used in this study

Dataset	Expression matrix	Metadata	Used for analysis
ASSESS	GEO (public) Raw + processed	Private	GEO (normalised) + metadata from internal source
PRECISESADS	ELIXIR (upon request) VST	ELIXIR (upon request)	TRANSMART files (PreciseSADS_SjS_Matched_Control_RNASEQ_VST; PreciseSADS_SjS_Matched_Control_CLINICAL^1^)
UKPSSR (GSE66795)	GEO (public) raw raw_data_matrix	Public	TRANSMART files (illumina_array_expression_data_normalized.tsv; illumina_array_phenodata.tsv)
GSE51092	GEO (public) RAW non-normalised	Public	Public (normalised)

### Statistical analyses

Differential expression analysis (DEA) was performed on R v4.3 using the limma pipeline^37^. STRING database was used to assess interaction relationships among intersecting genes and construct a protein-protein interaction (PPI) network^29^. Gene set enrichment analysis was performed using the *t*-test statistic to rank DEGs on the Hallmark pathways reference dataset (H)^38^. Overrepresentation Analysis on Reactome was performed by inputting significant total DEGs (FDR-adjusted *p*-value < 0.05 and logFC <-0.25 or >0.25) in the “Analyze gene list“ of the pathway analyzer present on their web-based server, with the options “Project to human” validated. Reactome overrepresented pathways were then filtered using an adjusted p-value (FDR < 0.05) and excluded disease maps. Significant pathways were compared between techniques, and shared identified biological species were selected for integration into the map.

### Map construction process, standards and annotations

**Fig. 8:**
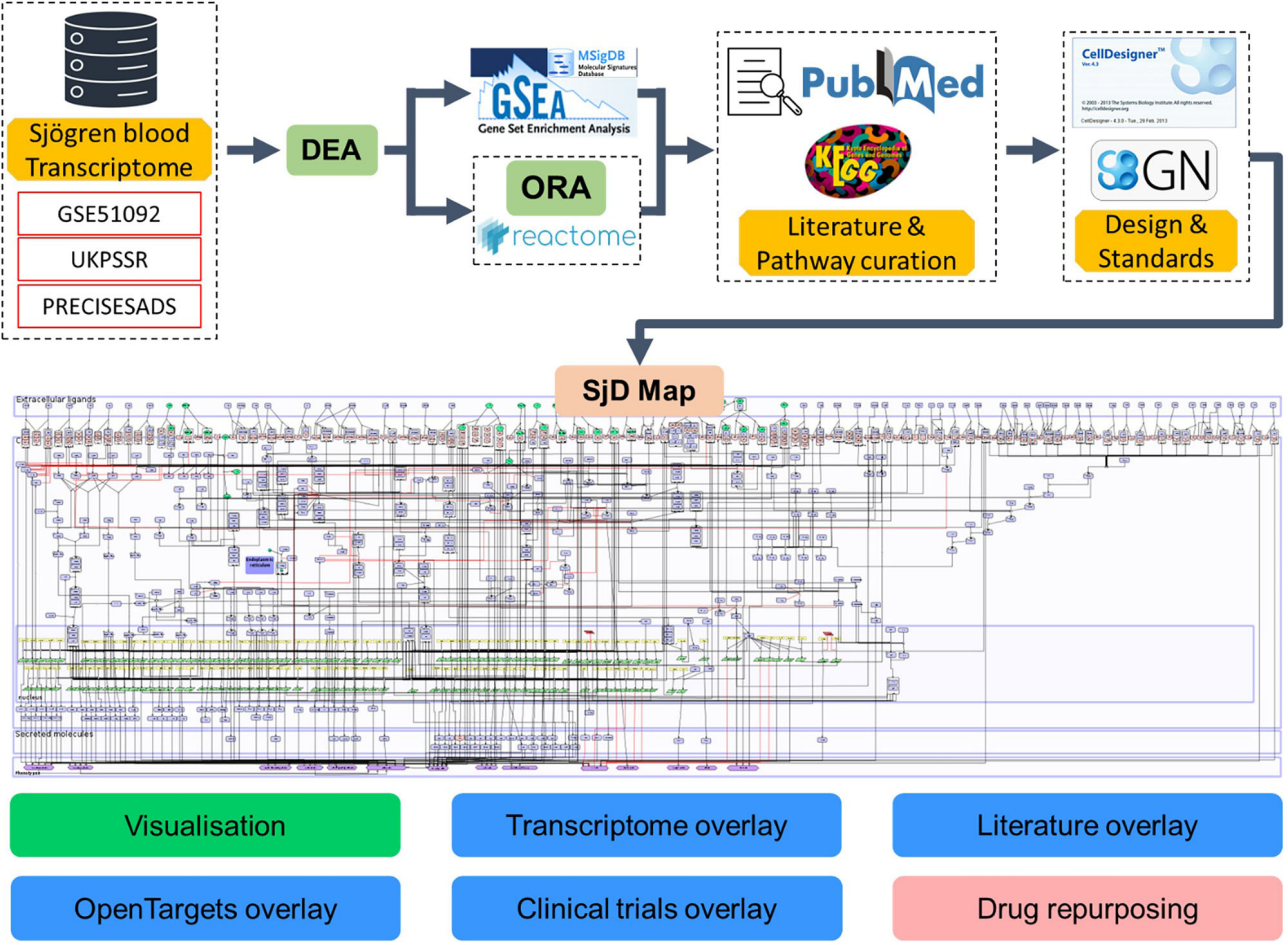
Workflow for building the Sjögren’s disease map. Differentially expressed genes from three transcriptomic datasets were analysed and subjected to pathway enrichment, and the results were then integrated with curated literature knowledge. These data were assembled into a Molecular Interaction Map incorporating experimental and clinical trial overlays to provide an interactive resource for exploring SjD mechanisms and therapeutic opportunities.

We used a framework for building disease maps based on a hybrid approach, combining literature, transcriptomic and pathways analyses inspired by the RA-map and RA-atlas^18,19^ (Fig. 8). It was built through a multi-step manual curation and integration process that combined data from pathway databases, transcriptomic analyses, and the literature. The overall goal was to produce a mechanistic, disease-specific network in Systems Biology Graphical Notation Process Description (SBGN-PD) format, suitable for visualisation and computational modelling. More specifically, the resource development included:

- Scaffold/skeleton construction: The network was primarily based on curated signalling pathways from Reactome, KEGG, and the literature, as well as network-based schemes. Relevant pathways were integrated using SjD–specific literature or pathways enriched in SjD transcriptomic analyses: network-based pathways from Reactome and descriptive pathways from GSEA. All pathways were manually adapted into SBGN-PD format during this process.
- Integration of descriptive pathways: GSEA results, along with specific Reactome pathways that are global pathway-level rather than network-level (e.g. IFN alpha signalling), were used to validate and complement the manually drawn pathways. Literature curation, including activity flow (AF) diagrams, guided the reconstruction of interactions not explicitly represented in existing databases.
- Handling overlapping nodes and interactions: Many pathways share common nodes or interactions. To avoid redundancy, identical nodes were unified based on standardised HGNC nomenclature, and interactions were merged when supported by multiple sources. Pathways were not duplicated based on their source, ensuring a single, coherent representation of each molecular entity.
- Criteria for inclusion/exclusion: Decisions were guided by expert assessment, with consideration of disease relevance, literature support, and transcriptomic evidence. Only components with sufficient mechanistic or experimental support were included.

The map was developed using the software CellDesigner (version 4.4.2)^39^. The CellDesigner maps are available in Systems Biology Markup Language (SBML) format. Information regarding species, reactions, and compartments of the map were referenced using MIRIAM (Minimal Information Requested In the Annotation of Models), a standard for annotating and curating computational models and maps. MIRIAM annotations are added in the CellDesigner dedicated section with the preceding “bqbiol: is describedby” term, which is used to define species or relationship between them according to the information source (e.g., PubMed references (PMIDs), DOI, KEGG identifier). When annotations could not be added into the MIRIAM section for technical issues, the pathway corresponding webpage link was added in the notes section of CellDesigner. Annotated maps provide confidence regarding the map specificity and source of information.

### Curation criteria: literature enrichment

First, a systematic Pubmed search was performed to extract biological species, reactions and pathway information about SjD. The search used the following keywords and criteria: (Sjögren[tiab] AND Human NOT mouse) AND (pathways OR cytokines OR Signal Transduction); Article type: Research support, Review, Systematic review; [en] articles; From 2010 until 2024. Were excluded and filtered manually from the search: Clinical studies; Drug/ethnopharmacology studies; General rheumatic/autoimmune diseases articles; Genetic association when not Caucasian; Intercellular studies (flow cytometry); Case reports; Microbiota studies; Dry eye/sicca without control studies. Besides this literature retrieval, a more precise search was performed using the “Sjögren” + “names of the biological species” to enhance literature enrichment. Articles focused on systemic autoimmune diseases were also added to enhance pathway/map connectivity.

### Visualisation and overlays

The SjD map is available as an interactive online map on MINERVA^21^. We provide eight downloadable data overlays that can be visualised directly on the map. PRECISESADS, UKPSSR, GSE51092 representing overexpressed genes in red and underexpressed in blue. Blood_transcriptome overlay that consists of the combination of all PRECISESADS, UKPSSR and GSE51092, in green.

ASSESS, an overlay highlighting species with DEGs in SjD patients who developed lymphoma (overexpressed genes in red and underexpressed in blue), was generated using data from the ASSESS cohort, a French longitudinal study of SjD patients. The associated transcriptomic data are available in the publicly available microarray dataset GSE140161/ASSESS via GEO^24^. Patients were classified based on their lymphoma status (25 of the 351 SjD patients in the ASSESS cohort) prior to differential expression analysis using the limma model, as detailed in the main text. Given the limited number of differentially expressed genes identified after stringent multiple-testing correction, we opted to retain genes with unadjusted *P* values < 0.05. This approach was justified by our focus on inferring pathway-level relevance rather than identifying individual differentially expressed genes.

GSE23117, a minor salivary gland dataset from a public microarray comprising 10 SjD patients and five controls, was used to generate an expression overlay, with overexpressed genes shown in red and underexpressed genes in blue. Differential expression analysis was performed using the limma model with FDR < 0.05.

A Literature overlay shows the number of publications associated with an entity by categories in white, yellow, light green, and dark green, respectively corresponding to 0, 1–5, 6–10, and more than 11 PMIDs.

Lastly, the map includes two OpenTargets overlays, that were created using the platform’s API: OpenTargets_Drugs used for identifying drug targets used in Sjögren’s clinical trials with a colour code associated to identify the stage of the clinical trial (green = completed; orange = Recruiting; purple = Not yet recruiting, Active not recruiting or unknown status; red = Withdrawn or terminated). OpenTargets_Validation was used for enrichment validation with a colour code associated to identify Sjögren’s (EFO_0000699) external sources of information (green = EuropePMC; red = genetic variant associations; yellow = Expression Atlas; orange = other databases)^40^^,41^. All data and scripts used to generate the drug and clinical-trial overlays are publicly available in the project repositories. Specifically, they can be found in the following folder: https://gitlab.com/genhotel/TheSjDMap/-/tree/main/Statistics_Overlays/Open_Targets?ref_type=heads. This directory contains both the scripts used to query the OpenTargets API and the downloaded data files (TSV/JSON). The data were retrieved from the OpenTargets platform (https://platform.opentargets.org/). For all Overlays, see the section in https://gitlab.com/genhotel/TheSjDMap/-/tree/main/Statistics_Overlays?ref_type=heads.

### Coverage and weights

We define coverage as the percentage of entities overlaid relative to the total number of entities in the map. Evidence categories and weights are as follows:

- For map creation: literature-curated interactions and blood transcriptome–enriched pathways. Each category is assigned a percentage based on the number of entities supported (as reported in the manuscript).
- For map validation, overlays indicate the proportion of entities supported by each dataset; the Upset plot shows these percentages for each evidence category.
- Data sources and dates:
- Literature sources are fully referenced with PMIDs and were integrated following the procedure described in Methods (covering publications from 2010 onward).
- The transcriptomic datasets and their corresponding dates are as follows: GSE51092 (2013), UKPSSR (2011), and PRECISESADS (2015).

The script used to calculate coverage, along with the input snapshot, is available at.https://gitlab.com/genhotel/TheSjDMap/-/blob/main/Statistics_Overlays/SjD_Map_validation.R?ref_type=heads.

### Ethics

As each dataset has already been published, written informed consent was obtained from all participants in accordance with the requirements of the institutional review board or ethics committee overseeing each respective study.

### Computational resources and runtimes

Versions and sources of all primary resources used in this study are listed below:

Hallmark gene sets: MSigDB v2024.1.Hs (retrieved August 2024); Reactome pathways: Reactome v86; OpenTargets platform: version 24.09; DrugBank: not used directly; DrugBank data are integrated through the MINERVA platform; MINERVA export: MINERVA version 19.0.1.

All analyses were performed on a DELL Latitude 14” laptop equipped with an Intel Core i7-1185G7 processor and 16 GB RAM. Each script for differential expression analysis (DEA) and gene set enrichment analysis (GSEA) typically runs in approximately 1–2 min under these conditions.

## Supplementary information

Supplementary Information

Supplementary Data S2

Supplementary Data S3

Supplementary Data S4

Supplementary Data S5

## Data Availability

The SjD Map is freely accessible at https://sjdmap.elixir-luxembourg.org/. All PMIDs of the scientific articles used for map construction are available in the map’s annotation section. All data and code used to generate results, including XML files of the maps, confidence score calculations, overlays, and gene expression analysis, are available on the Zenodo: 10.5281/zenodo.15373342 and GitLab repository: https://gitlab.com/genhotel/TheSjDMap.
