# Supplementary material for: The SjD Map: an interactive pathway tour into Sjögren’s disease signalling mechanisms
Source: NPJ Syst Biol Appl. 2026 Mar 28;12:78. doi: 10.1038/s41540-026-00670-x (PMC13230593; doi:10.1038/s41540-026-00670-x)
Supplement: Supplementary file 1 — Supplementary Information [file 41540_2026_670_MOESM1_ESM.pdf]

Supplementary Information

Supplementary Data S1: PRECISESADS consortium members

#The PRECISESADS Clinical Consortium is composed of the following members:

Lorenzo Beretta1 , Barbara Vigone1 , Jacques-Olivier Pers2 , Alain Saraux2 , Valérie Devauchelle-Pensec2 , Divi Cornec2 , Sandrine Jousse-Joulin2 , Bernard Lauwerys3 , Julie Ducreux3 , Anne-Lise Maudoux3 , Carlos Vasconcelos4 , Ana Tavares4 , Esmeralda Neves4 , Raquel Faria4 , Mariana Brandão4 , Ana Campar4 , António Marinho4 , Fátima Farinha4 , Isabel Almeida4 , Miguel Angel Gonzalez-Gay Montecón5 , Ricardo Blanco Alonso5 , Alfonso Corrales Martinez5 , Ricard Cervera6 , Ignasi Rodríguez-Pintó6 , Gerard Espinosa6 , Rik Lories7 , Ellen De Langhe7 , Nicolas Huzelmann8 , Doreen Belz8 , Torsten Witte9 , Niklas Baerlecken9 , Georg Stummvoll10, Michael Zauner10, Michaela Lehner10, Eduardo Collantes11, Rafaela Ortega-Castro11, Ma Angeles Aguirre-Zamorano11, Alejandro Escudero-Contreras11, Ma Carmen Castro-Villegas11, Norberto Ortego12, María Concepción Fernández Roldán12, Enrique Raya13, Immaculada Jiménez Moleón13, Enrique de Ramon14, Isabel Díaz Quintero14, Pier Luigi Meroni15, Maria Gerosa15, Tommaso Schioppo15, Carolina Artusi15, Carlo Chizzolini16, Aleksandra Zuber16, Donatienne Wynar16, Laszló Kovács17, Attila Balog17, Magdolna Deák17, Márta Bocskai17, Sonja Dulic17, Gabriella Kádár17, Falk Hiepe18, Velia Gerl18, Silvia Thiel18, Manuel Rodriguez Maresca19, Antonio López-Berrio19, Rocío Aguilar-Quesada19, Héctor Navarro-Linares19, and Marta E. Alarcon-Riquelme20.

1 Referral Center for Systemic Autoimmune Diseases, Fondazione IRCCS Ca' Granda Ospedale Maggiore Policlinico di Milano, Italy; 2 Centre Hospitalier Universitaire de Brest, Hospital de la Cavale Blanche, Brest, France; 3 Pôle de pathologies rhumatismales systémiques et inflammatoires, Institut de Recherche Expérimentale et Clinique, Université catholique de Louvain, Brussels, Belgium; 4 Centro Hospitalar do Porto, Portugal; 5 Servicio Cantabro de Salud, Hospital Universitario Marqués de Valdecilla, Santander, Spain; 6 Hospital Clinic I Provicia, Institut d'Investigacions Biomèdiques August Pi i Sunyer, Barcelona, Spain; 7 Katholieke Universiteit Leuven, Belgium; 8 Klinikum der Universitaet zu Koeln, Cologne, Germany; 9 Medizinische Hochschule Hannover, Germany; 10Medical University Vienna, Vienna, Austria; 11Servicio Andaluz de Salud, Hospital Universitario Reina Sofía Córdoba, Spain; 12Servicio Andaluz de Salud, Complejo hospitalario Universitario de Granada (Hospital Universitario San Cecilio), Spain; 13Servicio Andaluz de Salud, Complejo hospitalario Universitario de Granada (Hospital Virgen de las Nieves), Spain; 14Servicio Andaluz de Salud, Hospital Regional Universitario de Málaga, Spain; 15Università degli studi di Milano, Milan, Italy; 16Hospitaux Universitaires de Genève, Switzerland; 17University of Szeged, Szeged, Hungary; 18Charite, Berlin, Germany; 19Andalusian Public Health System Biobank, Granada, Spain; 20Genyo, Center for Genomics and Oncological Research, Pfizer/University of Granada/Andalusian Regional Government, Granada, Spain.

The study was approved by the following ethic committees: Comitato Etico Area 2 (Fondazione IRCCS Ca' Granda Ospedale Maggiore Policlinico di Milano and University of

Milan); approval no. 425bis Nov 19, 2014, and no. 671_2018 Sep 19, 2018; Klinikum der Universitaet zu Koeln, Cologne, Germany. Geschaftsstelle Ethikkommission; Pôle de pathologies rhumatismales systémiques et inflammatoires, Institut de Recherche Expérimentale et Clinique, Université catholique de Louvain, Brussels, Belgium. Comité d`Èthique Hospitalo-Facultaire; University of Szeged, Szeged, Hungary. Csongrad Megyei Kormanyhivatal; Hospital Clinic I Provicia, Institut d'Investigacions Biomèdiques August Pi i Sunyer, Barcelona, Spain. Comité Ética de Investigación Clínica del Hospital Clínic de Barcelona. Hospital Clinic del Barcelona; Servicio Andaluz de Salud, Hospital Universitario Reina Sofía Córdoba, Spain. Comité de Ética e la Investigación de Centro de Granada (CEI – Granada); Centro Hospitalar do Porto, Portugal. Comissao de ética para a Saude – CES do CHP; Centre Hospitalier Universitaire de Brest, Hospital de la Cavale Blanche, Avenue Tanguy Prigent 29609, Brest, France. Comite de Protection des Personnes Ouest VI; Hospitaux Universitaires de Genève, Switzerland. DEAS – Commission Cantonale d'ethique de la recherche Hopitaux universitaires de Geneve; Andalusian Public Health System Biobank, Granada, Spain; Katholieke Universiteit Leuven, Belgium. Commissie Medische Ethiek UZ KU Leuven /Onderzoek; Charite, Berlin, Germany. Ethikkommission; Medizinische Hochschule Hannover, Germany. Ethikkommission. PRECISESADS Study was funded by the Innovative Medicines Initiative of the European Union with grant number 115565 partly supported by the EFPIA Companies (Alarcon-Riquelme).

Supplementary Data S2: Differentially expressed genes (DEGs) identified in Sjögren's disease (SjD) versus healthy controls across the GSE51092, PRECISESADS, and UKPSSR datasets

Supplementary Data S3: Results of Reactome pathway enrichment analysis performed on the merged DEGs derived from the GSE51092, PRECISESADS, and UKPSSR datasets

Supplementary Data S4: Detailed graphical Reactome Pathway Analysis Report for the merged DEGs from GSE51092, PRECISESADS, and UKPSSR

Supplementary Data S5: DEGs identified in the ASSESS cohort, comparing lymphoma versus non-lymphoma SjD patients
