# Supplementary material for: The SjD Map: an interactive pathway tour into Sjögren’s disease signalling mechanisms
Source: NPJ Syst Biol Appl. 2026 Mar 28;12:78. doi: 10.1038/s41540-026-00670-x (PMC13230593; doi:10.1038/s41540-026-00670-x)
Supplement: Supplementary file 4 — Supplementary Data S4 [file 41540_2026_670_MOESM4_ESM.pdf]

Pathway Analysis Report

This report contains the pathway analysis results for the submitted sample ''. Analysis was performed against Reactome version 86 on 06/12/2023. The web link to these results is:

https://reactome.org/PathwayBrowser/#/ANALYSIS=MjAyMzEyMDYwOTU0MzdfNDc3Nw%3D%3D

Please keep in mind that analysis results are temporarily stored on our server. The storage period depends on usage of the service but is at least 7 days. As a result, please note that this URL is only valid for a limited time period and it might have expired.

Table of Contents

1. Introduction
2. Properties
3. Genome-wide overview
4. Most significant pathways
5. Pathways details
6. Identifiers found
7. Identifiers not found

1. Introduction

Reactome is a curated database of pathways and reactions in human biology. Reactions can be considered as pathway 'steps'. Reactome defines a 'reaction' as any event in biology that changes the state of a biological molecule. Binding, activation, translocation, degradation and classical biochemical events involving a catalyst are all reactions. Information in the database is authored by expert biologists, entered and maintained by Reactome's team of curators and editorial staff. Reactome content frequently cross-references other resources e.g. NCBI, Ensembl, UniProt, KEGG (Gene and Compound), ChEBI, PubMed and GO. Orthologous reactions inferred from annotation for Homo sapiens are available for 14 non-human species including mouse, rat, chicken, puffer fish, worm, fly and yeast. Pathways are represented by simple diagrams following an SBGN-like format.

Reactome's annotated data describe reactions possible if all annotated proteins and small molecules were present and active simultaneously in a cell. By overlaying an experimental dataset on these annotations, a user can perform a pathway over-representation analysis. By overlaying quantitative expression data or time series, a user can visualize the extent of change in affected pathways and its progression. A binomial test is used to calculate the probability shown for each result, and the p-values are corrected for the multiple testing (Benjamini–Hochberg procedure) that arises from evaluating the submitted list of identifiers against every pathway.

To learn more about our Pathway Analysis, please have a look at our relevant publications:

Fabregat A, Sidiropoulos K, Garapati P, Gillespie M, Hausmann K, Haw R, … D'Eustachio P (2016). The reactome pathway knowledgebase. Nucleic Acids Research, 44(D1), D481–D487. https://doi.org/10.1093/nar/gkv1351.

Fabregat A, Sidiropoulos K, Viteri G, Forner O, Marin-Garcia P, Arnau V, … Hermjakob H (2017). Reactome pathway analysis: a high-performance in-memory approach. BMC Bioinformatics, 18.

2. Properties

- This is an **overrepresentation** analysis: A statistical (hypergeometric distribution) test that determines whether certain Reactome pathways are over-represented (enriched) in the submitted data. It answers the question ‘Does my list contain more proteins for pathway X than would be expected by chance?’ This test produces a probability score, which is corrected for false discovery rate using the Benjamani-Hochberg method.
- 1084 out of 1660 identifiers in the sample were found in Reactome, where 1805 pathways were hit by at least one of them.
- All non-human identifiers have been converted to their human equivalent.
- This report is filtered to show only results for species 'Homo sapiens' and resource 'all resources'.
- The unique ID for this analysis (token) is MjAyMzEyMDYwOTU0MzdfNDc3Nw%3D%3D. This ID is valid for at least 7 days in Reactome's server. Use it to access Reactome services with your data.

3. Genome-wide overview

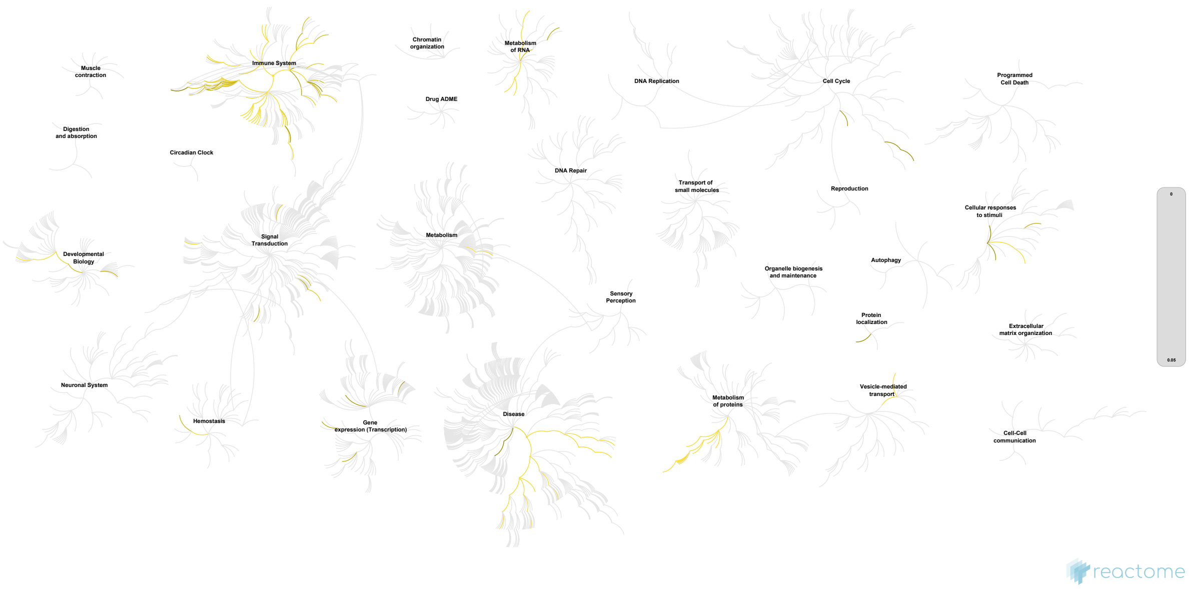

This figure shows a genome-wide overview of the results of your pathway analysis. Reactome pathways are arranged in a hierarchy. The center of each of the circular "bursts" is the root of one top-level pathway, for example "DNA Repair". Each step away from the center represents the next level lower in the pathway hierarchy. The color code denotes over-representation of that pathway in your input dataset. Light grey signifies pathways which are not significantly over-represented.

4. Most significant pathways

The following table shows the 25 most relevant pathways sorted by p-value.

Pathway name	Entities				Reactions	
	found	ratio	p-value	FDR*	found	ratio
Eukaryotic Translation Elongation	60 / 102	0.007	1.11e-16	1.35e-14	9 / 9	6.21e-04
Peptide chain elongation	57 / 97	0.006	1.11e-16	1.35e-14	5 / 5	3.45e-04
Nonsense Mediated Decay (NMD) independent of the Exon Junction Complex (EJC)	56 / 101	0.007	1.11e-16	1.35e-14	1 / 1	6.91e-05
Formation of a pool of free 40S subunits	57 / 106	0.007	1.11e-16	1.35e-14	2 / 2	1.38e-04
Nonsense-Mediated Decay (NMD)	61 / 124	0.008	1.11e-16	1.35e-14	6 / 6	4.14e-04
Nonsense Mediated Decay (NMD) enhanced by the Exon Junction Complex (EJC)	61 / 124	0.008	1.11e-16	1.35e-14	5 / 5	3.45e-04
L13a-mediated translational silencing of Ceruloplasmin expression	59 / 120	0.008	1.11e-16	1.35e-14	3 / 3	2.07e-04
GTP hydrolysis and joining of the 60S ribosomal subunit	58 / 120	0.008	1.11e-16	1.35e-14	3 / 3	2.07e-04
SRP-dependent cotranslational protein targeting to membrane	57 / 119	0.008	1.11e-16	1.35e-14	5 / 5	3.45e-04
Interferon gamma signaling	87 / 177	0.012	1.11e-16	1.35e-14	22 / 23	0.002
Interferon alpha/beta signaling	82 / 129	0.008	1.11e-16	1.35e-14	23 / 25	0.002
Interferon Signaling	153 / 398	0.026	1.11e-16	1.35e-14	109 / 119	0.008
Cytokine Signaling in Immune system	265 / 1,101	0.072	1.11e-16	1.35e-14	524 / 785	0.054
Immune System	525 / 2,661	0.173	1.11e-16	1.35e-14	1,091 / 1,704	0.118
Eukaryotic Translation Termination	55 / 106	0.007	1.11e-16	1.35e-14	3 / 5	3.45e-04
Response of EIF2AK4 (GCN2) to amino acid deficiency	57 / 115	0.007	1.11e-16	1.35e-14	6 / 16	0.001
Selenocysteine synthesis	55 / 112	0.007	2.22e-16	2.55e-14	2 / 7	4.83e-04
Viral mRNA Translation	55 / 114	0.007	4.44e-16	4.31e-14	2 / 2	1.38e-04
Eukaryotic Translation Initiation	59 / 130	0.008	4.44e-16	4.31e-14	14 / 21	0.001
Cap-dependent Translation Initiation	59 / 130	0.008	4.44e-16	4.31e-14	11 / 18	0.001
Immunoregulatory interactions between a Lymphoid and a non-Lymphoid cell	83 / 249	0.016	2.14e-14	1.99e-12	33 / 44	0.003
Regulation of expression of SLITs and ROBOs	64 / 183	0.012	2.81e-12	2.48e-10	5 / 20	0.001
Cellular response to starvation	62 / 176	0.011	4.58e-12	3.89e-10	18 / 28	0.002

Pathway name	Entities				Reactions	
	found	ratio	p-value	FDR*	found	ratio
**SARS-CoV-1 modulates host translation machinery**	27 / 41	0.003	1.56e-11	1.26e-09	2 / 3	2.07e-04
**FCGR activation**	44 / 103	0.007	1.66e-11	1.30e-09	6 / 6	4.14e-04

* False Discovery Rate

5. Pathways details

For every pathway of the most significant pathways, we present its diagram, as well as a short summary, its bibliography and the list of inputs found in it.

1. Eukaryotic Translation Elongation (R-HSA-156842)

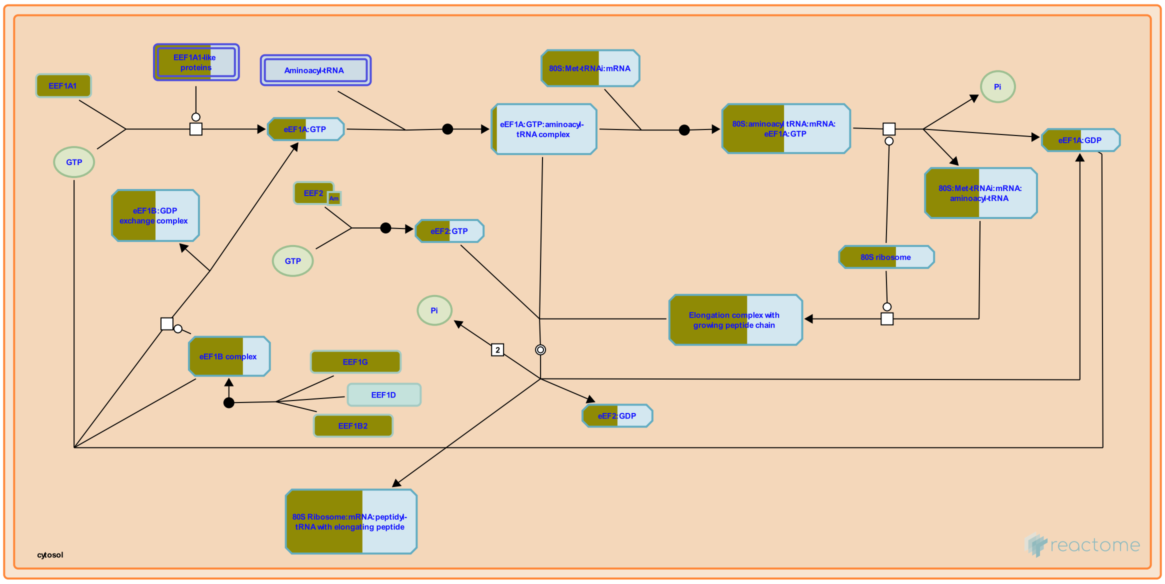

Cellular compartments: cytosol.

The translation elongation cycle adds one amino acid at a time to a growing polypeptide according to the sequence of codons found in the mRNA. The next available codon on the mRNA is exposed in the aminoacyl-tRNA (aa-tRNA) binding site (A site) on the 30S subunit.

A: Ternary complexes of aa -tRNA:eEF1A:GTP enter the ribosome and enable the anticodon of the tRNA to make a codon/anticodon interaction with the A-site codon of the mRNA. B: Upon cognate recognition, the eEF1A:GTP is brought into the GTPase activating center of the ribosome, GTP is hydrolyzed and eEF1A:GDP leaves the ribosome. C: The peptidyl transferase center of ribosome catalyses the formation of a peptide bond between the incoming amino acid and the peptide found in the peptidyl-tRNA binding site (P site). D: In the pre-translocation state of the ribosome, the eEF2:GTP enters the ribosome, physically translocating the peptidyl-tRNA out of the A site to P site and leaves the ribosome eEF2:GDP. This action of eEF2:GTP accounts for the precise movement of the mRNA by 3 nucleotides.Consequently, deacylated tRNA is shifted to the E site. A ribosome associated ATPase activity is proposed to stimulate the release of deacylated tRNA from the E site subsequent to translocation (Elskaya et al., 1991). In this post-translocation state, the ribosome is now ready to receive a new ternary complex.

This process is illustrated below with: an amino acyl-tRNA with an amino acid, a peptidyl-tRNA with a growing peptide, a deacylated tRNA with an -OH, and a ribosome with A,P and E sites to accommodate these three forms of tRNA.

References

Kapp LD & Lorsch JR (2004). The molecular mechanics of eukaryotic translation. Annu Rev Biochem, 73, 657-704.

Edit history

Date	Action	Author
2004-12-14	Created	Balar B, Ulloque R, Ortiz PA, Pittman YR, Kinzy TG et al.
2005-03-13	Authored	Gopinathrao G
2023-08-26	Modified	Wright A

57 submitted entities found in this pathway, mapping to 71 Reactome entities

Input	UniProt Id	Input	UniProt Id	Input	UniProt Id
AGRN	P32969	EEF1A1	P68104	EEF1A1P5	Q5VTE0
EEF1B2	P24534	EEF1G	P26641	EEF2	P13639
FAU	P62861	RPL10	P27635, Q96L21	RPL10A	P61313, P62906
RPL13	P26373, P40429	RPL13A	P40429, P61313	RPL15	P61313
RPL18	Q07020	RPL21	P46778	RPL27A	P46776, P61353
RPL29	P47914	RPL3	P39023, Q92901	RPL30	P62888
RPL31	P62899	RPL32	P62888, P62910	RPL34	P49207, P62899
RPL35	P42766	RPL35A	P18077	RPL36	Q9Y3U8
RPL38	P63173	RPL3L	Q92901	RPL4	P36578
RPL5	P46777	RPL7	P18124	RPL7A	P18124, P62424
RPL9P8	P32969	RPLP0	P05388	RPLP2	P05387
RPS10	P46783	RPS11	P62280	RPS12	P25398
RPS13	P62277	RPS14	P62263	RPS15	P62841
RPS15A	P62244	RPS16	P62249	RPS17	P08708
RPS18	P62269	RPS2	P15880, P46782	RPS23	P62266
RPS27	P42677, Q71UM5	RPS27A	P42677, P62979, P62987	RPS29	P62273
RPS3	P23396	RPS3A	P61247	RPS4X	P62701, Q8TD47
RPS5	P46782	RPS6	P62753	RPS8	P62241
RPS9	P46781	RPSA	P08865	SOS2	P42766

2. Peptide chain elongation (R-HSA-156902)

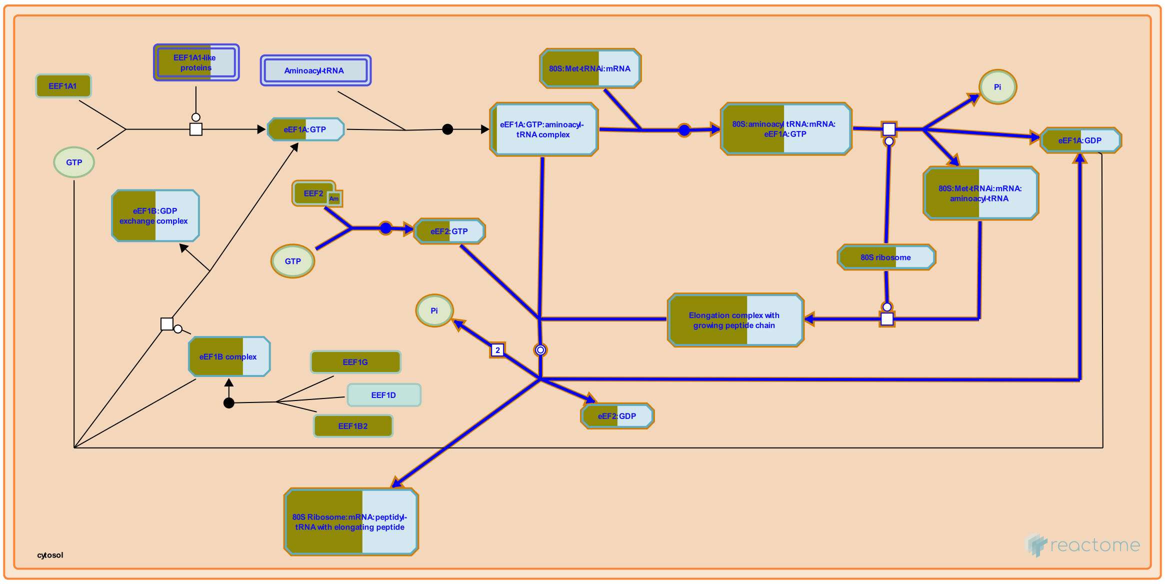

Cellular compartments: cytosol.

The mechanism of a peptide bond requires the movement of three protons. First the deprotonation of the ammonium ion generates a reactive amine, allowing a nucleophilic attack on the carbonyl group. This is followed by the loss of a proton from the reaction intermediate, only to be taken up by the oxygen on the leaving group (from the end of the amino acid chain bound to the tRNA in the P-site). The peptide bond formation results in the net loss of one water molecule, leaving a deacylated-tRNA in the P-site, and a nascent polypeptide chain one amino acid larger in the A-site.

For the purpose of illustration, the figures used in the section show one amino acid being added to a peptidyl-tRNA with a growing peptide chain.

References

Lorsch JR & Green R (2002). The path to perdition is paved with protons. Cell, 110, 665-8.

Edit history

Date	Action	Author
2005-03-13	Authored	Gopinathrao G
2005-03-17	Created	Balar B, Ulloque R, Kinzy TG
2023-08-31	Modified	Wright A

54 submitted entities found in this pathway, mapping to 68 Reactome entities

Input	UniProt Id	Input	UniProt Id	Input	UniProt Id
AGRN	P32969	EEF1A1	P68104	EEF2	P13639
FAU	P62861	RPL10	P27635, Q96L21	RPL10A	P61313, P62906
RPL13	P26373, P40429	RPL13A	P40429, P61313	RPL15	P61313
RPL18	Q07020	RPL21	P46778	RPL27A	P46776, P61353
RPL29	P47914	RPL3	P39023, Q92901	RPL30	P62888
RPL31	P62899	RPL32	P62888, P62910	RPL34	P49207, P62899
RPL35	P42766	RPL35A	P18077	RPL36	Q9Y3U8

Input	UniProt Id	Input	UniProt Id	Input	UniProt Id
RPL38	P63173	RPL3L	Q92901	RPL4	P36578
RPL5	P46777	RPL7	P18124	RPL7A	P18124, P62424
RPL9P8	P32969	RPLP0	P05388	RPLP2	P05387
RPS10	P46783	RPS11	P62280	RPS12	P25398
RPS13	P62277	RPS14	P62263	RPS15	P62841
RPS15A	P62244	RPS16	P62249	RPS17	P08708
RPS18	P62269	RPS2	P15880, P46782	RPS23	P62266
RPS27	P42677, Q71UM5	RPS27A	P42677, P62979, P62987	RPS29	P62273
RPS3	P23396	RPS3A	P61247	RPS4X	P62701, Q8TD47
RPS5	P46782	RPS6	P62753	RPS8	P62241
RPS9	P46781	RPSA	P08865	SOS2	P42766

3. Nonsense Mediated Decay (NMD) independent of the Exon Junction Complex (EJC) (R-HSA-975956)

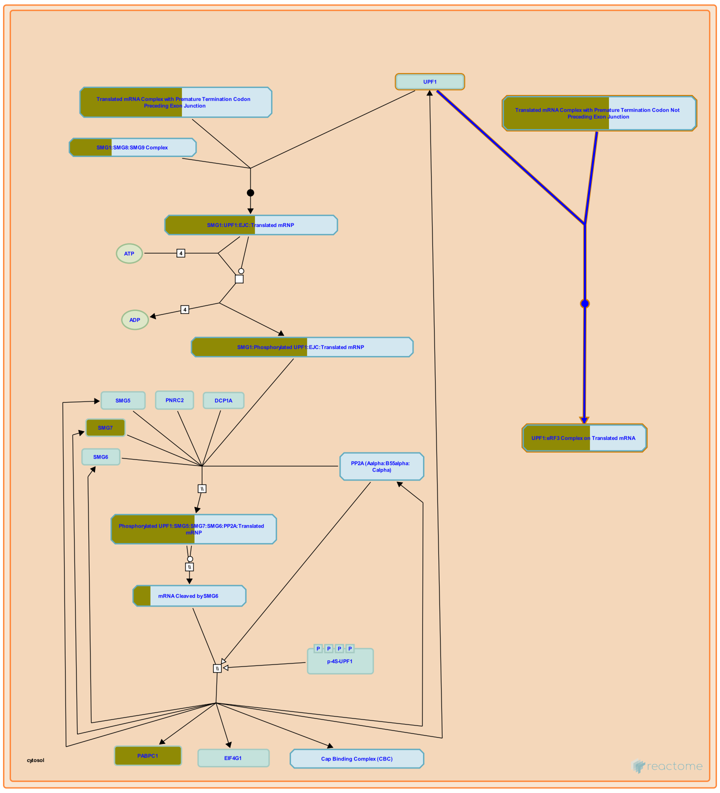

Cellular compartments: cytosol.

Nonsense-mediated decay has been observed with mRNAs that do not have an exon junction complex (EJC) downstream of the termination codon (reviewed in Isken and Maquat 2007, Chang et al. 2007, Behm-Ansmant et al. 2007, Rebbapragada and Lykke-Andersen 2009, Nicholson et al. 2010). In these cases the trigger is unknown but a correlation with the length of the 3' UTR has sometimes been seen. The current model posits a competition between PABP and UPF1 for access to eRF3 at the terminating ribosome (Ivanov et al. 2008, Singh et al. 2008, reviewed in Bhuvanagiri et al. 2010). Abnormally long 3' UTRs may prevent PABP from efficiently interacting with eRF3 and allow UPF1 to bind eRF3 instead. Long UTRs with hairpin loops may bring PABP closer to eRF3 and help evade NMD (Eberle et al. 2008).

The pathway of degradation taken during EJC-independent NMD has not been elucidated. It is thought that phosphorylation of UPF1 by SMG1 and recruitment of SMG6 or SMG5 and SMG7 are involved, as with EJC-enhanced NMD, but this has not yet been shown.

References

Yepiskoposyan H, Kleinschmidt N, Zamudio Orozco R, Metze S, Muhlemann O & Nicholson P (2010). Nonsense-mediated mRNA decay in human cells: mechanistic insights, functions beyond quality control and the double-life of NMD factors. Cell Mol Life Sci, 67, 677-700.

Stalder L, Eberle AB, Mathys H, Orozco RZ & Muhlemann O (2008). Posttranscriptional gene regulation by spatial rearrangement of the 3' untranslated region. PLoS Biol, 6, e92.

Saulière J, Wittkopp N, Behm-Ansmant I, Izaurralde E, Rehwinkel J & Kashima I (2007). mRNA quality control: an ancient machinery recognizes and degrades mRNAs with nonsense codons. FEBS Lett, 581, 2845-53.

Rebbapragada I, Singh G & Lykke-Andersen J (2008). A competition between stimulators and antagonists of Upf complex recruitment governs human nonsense-mediated mRNA decay. PLoS Biol, 6, e111.

Bhuvanagiri M, Kulozik AE, Hentze MW & Schlitter AM (2010). NMD: RNA biology meets human genetic medicine. Biochem J, 430, 365-77.

Edit history

Date	Action	Author
2010-10-08	Edited	May B
2010-10-08	Authored	May B
2010-10-11	Created	May B
2011-05-19	Reviewed	Neu-Yilik G
2023-08-31	Modified	Wright A

53 submitted entities found in this pathway, mapping to 67 Reactome entities

Input	UniProt Id	Input	UniProt Id	Input	UniProt Id
AGRN	P32969	FAU	P62861	PABPC1	P11940
RPL10	P27635, Q96L21	RPL10A	P61313, P62906	RPL13	P26373, P40429
RPL13A	P40429, P61313	RPL15	P61313	RPL18	Q07020
RPL21	P46778	RPL27A	P46776, P61353	RPL29	P47914
RPL3	P39023, Q92901	RPL30	P62888	RPL31	P62899
RPL32	P62888, P62910	RPL34	P49207, P62899	RPL35	P42766
RPL35A	P18077	RPL36	Q9Y3U8	RPL38	P63173
RPL3L	Q92901	RPL4	P36578	RPL5	P46777
RPL7	P18124	RPL7A	P18124, P62424	RPL9P8	P32969
RPLP0	P05388	RPLP2	P05387	RPS10	P46783
RPS11	P62280	RPS12	P25398	RPS13	P62277
RPS14	P62263	RPS15	P62841	RPS15A	P62244
RPS16	P62249	RPS17	P08708	RPS18	P62269
RPS2	P15880, P46782	RPS23	P62266	RPS27	P42677, Q71UM5
RPS27A	P42677, P62979, P62987	RPS29	P62273	RPS3	P23396
RPS3A	P61247	RPS4X	P62701, Q8TD47	RPS5	P46782
RPS6	P62753	RPS8	P62241	RPS9	P46781
RPSA	P08865	SOS2	P42766		

4. Formation of a pool of free 40S subunits (R-HSA-72689)

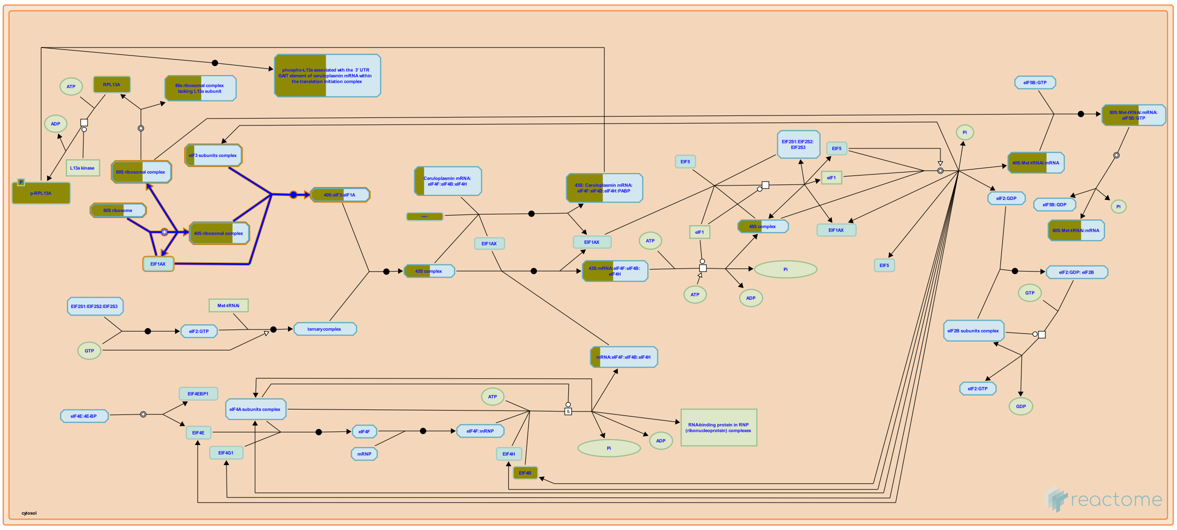

Cellular compartments: cytosol.

The 80S ribosome dissociates into free 40S (small) and 60S (large) ribosomal subunits. Each ribosomal subunit is constituted by several individual ribosomal proteins and rRNA.

References

Edit history

Date	Action	Author
2023-08-31	Modified	Wright A

54 submitted entities found in this pathway, mapping to 68 Reactome entities

Input	UniProt Id	Input	UniProt Id	Input	UniProt Id
AGRN	P32969	EIF3F	O00303	EIF3L	Q9Y262
FAU	P62861	RPL10	P27635, Q96L21	RPL10A	P61313, P62906
RPL13	P26373, P40429	RPL13A	P40429, P61313	RPL15	P61313
RPL18	Q07020	RPL21	P46778	RPL27A	P46776, P61353
RPL29	P47914	RPL3	P39023, Q92901	RPL30	P62888
RPL31	P62899	RPL32	P62888, P62910	RPL34	P49207, P62899
RPL35	P42766	RPL35A	P18077	RPL36	Q9Y3U8
RPL38	P63173	RPL3L	Q92901	RPL4	P36578
RPL5	P46777	RPL7	P18124	RPL7A	P18124, P62424
RPL9P8	P32969	RPLP0	P05388	RPLP2	P05387
RPS10	P46783	RPS11	P62280	RPS12	P25398
RPS13	P62277	RPS14	P62263	RPS15	P62841
RPS15A	P62244	RPS16	P62249	RPS17	P08708
RPS18	P62269	RPS2	P15880, P46782	RPS23	P62266
RPS27	P42677, Q71UM5	RPS27A	P42677, P62979, P62987	RPS29	P62273
RPS3	P23396	RPS3A	P61247	RPS4X	P62701, Q8TD47
RPS5	P46782	RPS6	P62753	RPS8	P62241
RPS9	P46781	RPSA	P08865	SOS2	P42766

5. Nonsense-Mediated Decay (NMD) (R-HSA-927802)

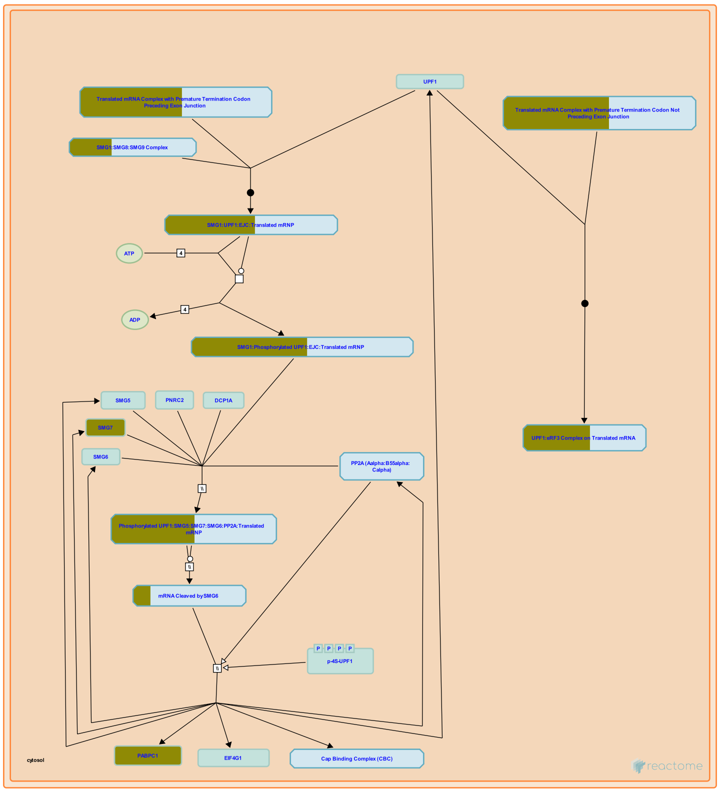

Cellular compartments: cytosol.

The Nonsense-Mediated Decay (NMD) pathway activates the destruction of mRNAs containing premature termination codons (PTCs) (reviewed in Isken and Maquat 2007, Chang et al. 2007, Behm-Ansmant et al. 2007, Neu-Yilik and Kulozik 2008, Rebbapragada and Lykke-Andersen 2009, Bhuvanagiri et al. 2010, Nicholson et al. 2010, Durand and Lykke-Andersen 2011). In mammalian cells a termination codon can be recognized as premature if it precedes an exon-exon junction by at least 50-55 nucleotides or if it is followed by an abnormal 3' untranslated region (UTR). While length of the UTR may play a part, the qualifications for being "abnormal" have not been fully elucidated. Also, some termination codons preceding exon junctions are not degraded by NMD so the criteria for triggering NMD are not yet fully known (reviewed in Rebbapragada and Lykke-Andersen 2009). While about 30% of disease-associated mutations in humans activate NMD, about 10% of normal human transcripts are also degraded by NMD (reviewed in Stalder and Muhlemann 2008, Neu-Yilik and Kulozik 2008, Bhuvanagiri et al. 2010, Nicholson et al. 2010). Thus NMD is a normal physiological process controlling mRNA stability in unmutated cells.

Exon junction complexes (EJCs) are deposited on an mRNA during splicing in the nucleus and are displaced by ribosomes during the first round of translation. When a ribosome terminates translation the A site encounters the termination codon and the eRF1 factor enters the empty A site and recruits eRF3. Normally, eRF1 cleaves the translated polypeptide from the tRNA in the P site and eRF3 interacts with Polyadenylate-binding protein (PABP) bound to the polyadenylated tail of the mRNA.

During activation of NMD eRF3 interacts with UPF1 which is contained in a complex with SMG1, SMG8, and SMG9. NMD can arbitrarily be divided into EJC-enhanced and EJC-independent pathways. In EJC-enhanced NMD, an exon junction is located downstream of the PTC and the EJC remains on the mRNA after termination of the pioneer round of translation. The core EJC is associated with UPF2 and UPF3, which interact with UPF1 and stimulate NMD. Once bound near the PTC, UPF1 is phosphorylated by SMG1. The phosphorylation is the rate-limiting step in NMD and causes UPF1 to recruit either SMG6, which is an endoribonuclease, or SMG5 and SMG7, which recruit ribonucleases. SMG6 and SMG5:SMG7 recruit phosphatase PP2A to dephosphorylate UPF1 and allow further rounds of degradation. How EJC-independent NMD is activated remains enigmatic but may involve competition between PABP and UPF1 for eRF3.

References

Yepiskoposyan H, Kleinschmidt N, Zamudio Orozco R, Metze S, Muhlemann O & Nicholson P (2010). Nonsense-mediated mRNA decay in human cells: mechanistic insights, functions beyond quality control and the double-life of NMD factors. Cell Mol Life Sci, 67, 677-700.

Saulière J, Wittkopp N, Behm-Ansmant I, Izaurralde E, Rehwinkel J & Kashima I (2007). mRNA quality control: an ancient machinery recognizes and degrades mRNAs with nonsense codons. FEBS Lett, 581, 2845-53.

Durand S & Lykke-Andersen J (2011). SnapShot: Nonsense-Mediated mRNA Decay. Cell, 145, 324-324.e2.

Bhuvanagiri M, Kulozik AE, Hentze MW & Schlitter AM (2010). NMD: RNA biology meets human genetic medicine. Biochem J, 430, 365-77.

Neu-Yilik G & Kulozik AE (2008). NMD: multitasking between mRNA surveillance and modulation of gene expression. Adv Genet, 62, 185-243.

Edit history

Date	Action	Author
2010-08-06	Edited	May B
2010-08-06	Authored	May B
2010-08-10	Created	May B
2011-05-19	Reviewed	Neu-Yilik G
2023-08-26	Modified	Wright A

58 submitted entities found in this pathway, mapping to 72 Reactome entities

Input	UniProt Id	Input	UniProt Id	Input	UniProt Id
AGRN	P32969	CASC3	O15234	FAU	P62861
MAGOH	P61326	PABPC1	P11940	RPL10	P27635, Q96L21
RPL10A	P61313, P62906	RPL13	P26373, P40429	RPL13A	P40429, P61313
RPL15	P61313	RPL18	Q07020	RPL21	P46778
RPL27A	P46776, P61353	RPL29	P47914	RPL3	P39023, Q92901
RPL30	P62888	RPL31	P62899	RPL32	P62888, P62910
RPL34	P49207, P62899	RPL35	P42766	RPL35A	P18077
RPL36	Q9Y3U8	RPL38	P63173	RPL3L	Q92901
RPL4	P36578	RPL5	P46777	RPL7	P18124

Input	UniProt Id	Input	UniProt Id	Input	UniProt Id
RPL7A	P18124, P62424	RPL9P8	P32969	RPLP0	P05388
RPLP2	P05387	RPS10	P46783	RPS11	P62280
RPS12	P25398	RPS13	P62277	RPS14	P62263
RPS15	P62841	RPS15A	P62244	RPS16	P62249
RPS17	P08708	RPS18	P62269	RPS2	P15880, P46782
RPS23	P62266	RPS27	P42677, Q71UM5	RPS27A	P42677, P62979, P62987
RPS29	P62273	RPS3	P23396	RPS3A	P61247
RPS4X	P62701, Q8TD47	RPS5	P46782	RPS6	P62753
RPS8	P62241	RPS9	P46781	RPSA	P08865
SMG7	Q92540	SMG9	Q9H0W8	SOS2	P42766
UPF3B	Q9BZI7				

6. Nonsense Mediated Decay (NMD) enhanced by the Exon Junction Complex (EJC) (R-HSA-975957)

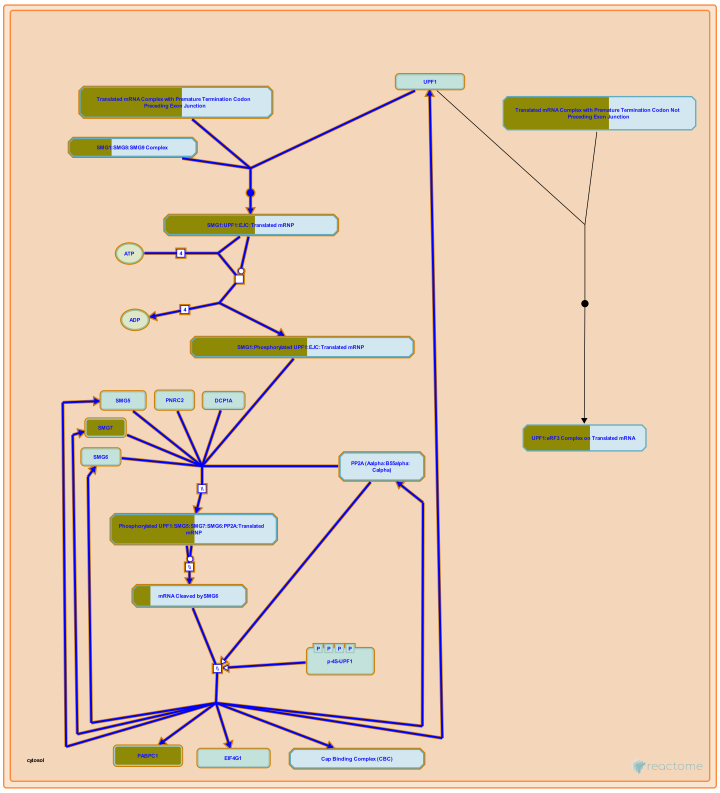

Cellular compartments: cytosol.

During normal translation termination eRF3 associates with the ribosome and then interacts with PABP bound to the polyadenylate tail of the mRNA to release the ribosome and allow a new round of translation to commence. Nonsense-mediated decay (NMD) is triggered if eRF3 at the ribosome interacts with UPF1, which may compete with PABP (reviewed in Isken and Maquat 2007, Chang et al. 2007, Behm-Ansmant et al. 2007, Rebbapragada and Lykke-Andersen 2009, Bhuvanagiri et al. 2010, Nicholson et al. 2010, Durand and Lykke-Andersen 2011). An exon junction located 50-55 nt downstream of a termination codon is observed to enhance NMD.

Exon-junction complexes (EJCs) are deposited on the mRNA during splicing in the nucleus, remain on mRNAs after transport to the cytosol, and are dislodged by the ribosome as it progresses along the mRNA during the pioneer round of translation (Gehring et al. 2009). EJCs contain the core factors eIF4A-III, Magoh-Y14, and CASC3 as well as the peripheral factors RNPS1, UPF2, and UPF3. UPF2 and UPF3 recruit UPF1 to eRF3 at the terminating ribosome. Thus an EJC downstream of a termination codon will not have been dislodged during translation and will recruit UPF1, triggering NMD.

UPF1 is believed to form a complex containing SMG1, SMG8, and SMG9. In the key regulatory step of NMD SMG1 phosphorylates UPF1. The phosphorylated UPF1 then recruits either SMG6 or SMG5 and SMG7. SMG6 is itself an endoribonuclease that cleaves the mRNA. SMG5 and SMG7 do not have endoribonuclease activty, but are thought to recruit ribonucleases. Nonsense-mediated decay has been observed to involve deadenlyation, decapping, and both 5' to 3' and 3' to 5' exonuclease activities, but the exact degradative pathways taken by a given mRNA are not yet known.

UPF1 also plays roles in Staufen-mediated decay, histone mRNA decay, telomere maintenance, genome integrity, and may play a role in normal termination of translation.

References

Yepiskoposyan H, Kleinschmidt N, Zamudio Orozco R, Metze S, Muhlemann O & Nicholson P (2010). Nonsense-mediated mRNA decay in human cells: mechanistic insights, functions beyond quality control and the double-life of NMD factors. Cell Mol Life Sci, 67, 677-700.

Saulière J, Wittkopp N, Behm-Ansmant I, Izaurralde E, Rehwinkel J & Kashima I (2007). mRNA quality control: an ancient machinery recognizes and degrades mRNAs with nonsense codons. FEBS Lett, 581, 2845-53.

Durand S & Lykke-Andersen J (2011). SnapShot: Nonsense-Mediated mRNA Decay. Cell, 145, 324-324.e2.

Gehring NH, Lamprinaki S, Kulozik AE & Hentze MW (2009). Disassembly of exon junction complexes by PYM. Cell, 137, 536-48.

Bhuvanagiri M, Kulozik AE, Hentze MW & Schlitter AM (2010). NMD: RNA biology meets human genetic medicine. Biochem J, 430, 365-77.

Edit history

Date	Action	Author
2010-10-08	Edited	May B
2010-10-08	Authored	May B
2010-10-11	Created	May B
2011-05-19	Reviewed	Neu-Yilik G
2023-08-31	Modified	Wright A

58 submitted entities found in this pathway, mapping to 72 Reactome entities

Input	UniProt Id	Input	UniProt Id	Input	UniProt Id
AGRN	P32969	CASC3	O15234	FAU	P62861
MAGOH	P61326	PABPC1	P11940	RPL10	P27635, Q96L21
RPL10A	P61313, P62906	RPL13	P26373, P40429	RPL13A	P40429, P61313
RPL15	P61313	RPL18	Q07020	RPL21	P46778
RPL27A	P46776, P61353	RPL29	P47914	RPL3	P39023, Q92901
RPL30	P62888	RPL31	P62899	RPL32	P62888, P62910
RPL34	P49207, P62899	RPL35	P42766	RPL35A	P18077
RPL36	Q9Y3U8	RPL38	P63173	RPL3L	Q92901
RPL4	P36578	RPL5	P46777	RPL7	P18124
RPL7A	P18124, P62424	RPL9P8	P32969	RPLP0	P05388
RPLP2	P05387	RPS10	P46783	RPS11	P62280
RPS12	P25398	RPS13	P62277	RPS14	P62263
RPS15	P62841	RPS15A	P62244	RPS16	P62249
RPS17	P08708	RPS18	P62269	RPS2	P15880, P46782
RPS23	P62266	RPS27	P42677, Q71UM5	RPS27A	P42677, P62979, P62987
RPS29	P62273	RPS3	P23396	RPS3A	P61247
RPS4X	P62701, Q8TD47	RPS5	P46782	RPS6	P62753

Input	UniProt Id	Input	UniProt Id	Input	UniProt Id
RPS8	P62241	RPS9	P46781	RPSA	P08865
SMG7	Q92540	SMG9	Q9H0W8	SOS2	P42766
UPF3B	Q9BZI7				

7. L13a-mediated translational silencing of Ceruloplasmin expression (R-HSA-156827)

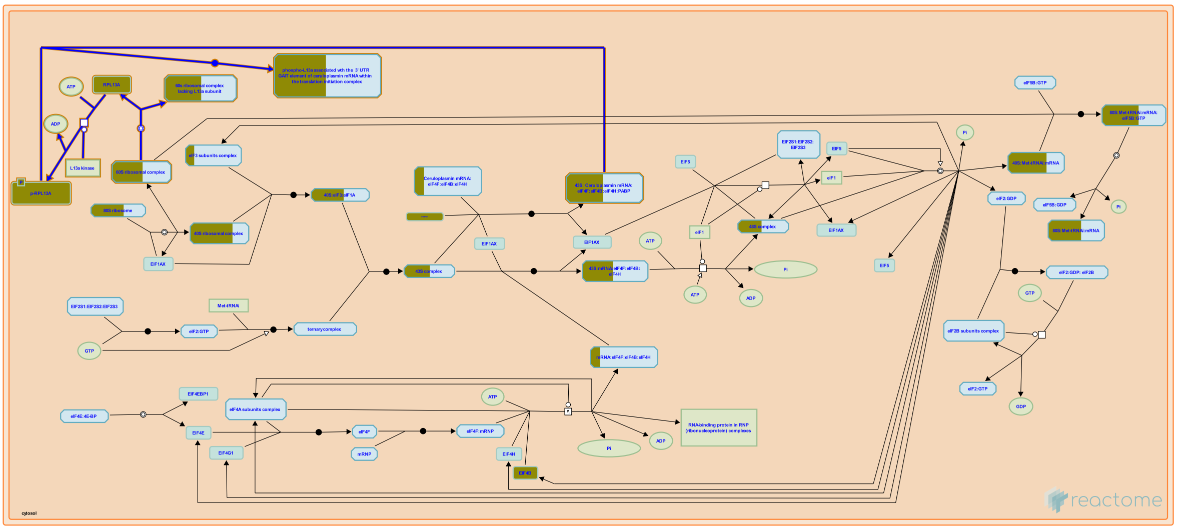

Cellular compartments: cytosol.

While circularization of mRNA during translation initiation is thought to contribute to an increase in the efficiency of translation, it also appears to provide a mechanism for translational silencing. This might be achieved by bringing inhibitory 3' UTR-binding proteins into a position in which they interfere either with the function of the translation initiation complex or with the assembly of the ribosome (Mazumder et al 2001). Translational silencing of Ceruloplasmin (Cp) occurs 16 hrs after its induction by INF-gamma (Mazumder et al., 1997). Although the mechanism by which silencing occurs has not yet been determined, this process is mediated by the L13a subunit of the 60s ribosome and thought to require circularization of the Cp mRNA (Sampath et al., 2003; Mazumder et al., 2001; Mazumder et al., 2003). Between 14 and 16 hrs after INF gamma induction, the L13a subunit of the 60s ribosome is phosphorylated and released from the 60s subunit. Phosphorylated L13a then associates with the GAIT element in the 3' UTR of the Cp mRNA inhibiting its translation.

References

Seshadri V, Sampath P, DiCorleto PE, Maitra RK, Fox PL & Mazumder B (2003). Regulated release of L13a from the 60S ribosomal subunit as a mechanism of transcript-specific translational control . Cell, 115, 187-98.

Edit history

Date	Action	Author
2004-12-13	Authored	Matthews L
2004-12-20	Created	Gebauer F
2013-11-25	Edited	Matthews L
2023-08-31	Modified	Wright A

56 submitted entities found in this pathway, mapping to 70 Reactome entities

Input	UniProt Id	Input	UniProt Id	Input	UniProt Id
AGRN	P32969	EIF3F	O00303	EIF3L	Q9Y262

Input	UniProt Id	Input	UniProt Id	Input	UniProt Id
EIF4B	P23588	FAU	P62861	PABPC1	P11940
RPL10	P27635, Q96L21	RPL10A	P61313, P62906	RPL13	P26373, P40429
RPL13A	P40429, P61313	RPL15	P61313	RPL18	Q07020
RPL21	P46778	RPL27A	P46776, P61353	RPL29	P47914
RPL3	P39023, Q92901	RPL30	P62888	RPL31	P62899
RPL32	P62888, P62910	RPL34	P49207, P62899	RPL35	P42766
RPL35A	P18077	RPL36	Q9Y3U8	RPL38	P63173
RPL3L	Q92901	RPL4	P36578	RPL5	P46777
RPL7	P18124	RPL7A	P18124, P62424	RPL9P8	P32969
RPLP0	P05388	RPLP2	P05387	RPS10	P46783
RPS11	P62280	RPS12	P25398	RPS13	P62277
RPS14	P62263	RPS15	P62841	RPS15A	P62244
RPS16	P62249	RPS17	P08708	RPS18	P62269
RPS2	P15880, P46782	RPS23	P62266	RPS27	P42677, Q71UM5
RPS27A	P42677, P62979, P62987	RPS29	P62273	RPS3	P23396
RPS3A	P61247	RPS4X	P62701, Q8TD47	RPS5	P46782
RPS6	P62753	RPS8	P62241	RPS9	P46781
RPSA	P08865	SOS2	P42766		

8. GTP hydrolysis and joining of the 60S ribosomal subunit (R-HSA-72706)

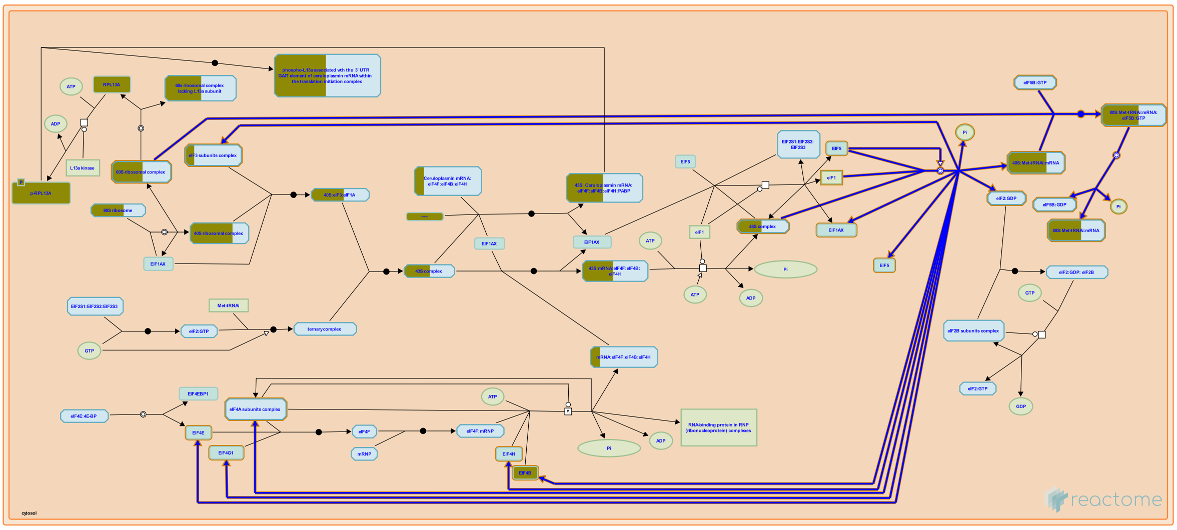

Hydrolysis of eIF2-GTP occurs after the Met-tRNAi has recognized the AUG. This reaction is catalyzed by eIF5 (or eIF5B) and is thought to cause dissociation of all other initiation factors and allow joining of the large 60S ribosomal subunit. The 60S subunit joins - a reaction catalyzed by eIF5 or eIF5B - resulting in a translation-competent 80S ribosome. Following 60S subunit joining, eIF5B hydrolyzes its GTP and is released from the 80S ribosome, which is now ready to start elongating the polypeptide chain.

References

Anderson WF, Merrick WC & Kemper WM (1975). Purification and characterization of homogeneous initiation factor M2A from rabbit reticulocytes. J Biol Chem, 250, 5556-62.

Erni B, Schreier MH, Trachsel H & Staehelin T (1978). Initiation of mammalian protein synthesis. II. The assembly of the initiation complex with purified initiation factors. J Mol Biol, 116, 755-67.

Benne R & Hershey JW (1978). The mechanism of action of protein synthesis initiation factors from rabbit reticulocytes. J Biol Chem, 253, 3078-87.

Choi SK, Dever TE, Lomakin IB, Lee JH, Hellen CU & Pestova TV (2000). The joining of ribosomal subunits in eukaryotes requires eIF5B. Nature, 403, 332-5.

Maitra U & Chakrabarti A (1991). Function of eukaryotic initiation factor 5 in the formation of an 80 S ribosomal polypeptide chain initiation complex. J Biol Chem, 266, 14039-45.

Edit history

Date	Action	Author
2002-12-16	Created	Merrick WC
2023-08-31	Modified	Wright A

55 submitted entities found in this pathway, mapping to 69 Reactome entities

Input	UniProt Id	Input	UniProt Id	Input	UniProt Id
AGRN	P32969	EIF3F	O00303	EIF3L	Q9Y262
EIF4B	P23588	FAU	P62861	RPL10	P27635, Q96L21

Input	UniProt Id	Input	UniProt Id	Input	UniProt Id
RPL10A	P61313, P62906	RPL13	P26373, P40429	RPL13A	P40429, P61313
RPL15	P61313	RPL18	Q07020	RPL21	P46778
RPL27A	P46776, P61353	RPL29	P47914	RPL3	P39023, Q92901
RPL30	P62888	RPL31	P62899	RPL32	P62888, P62910
RPL34	P49207, P62899	RPL35	P42766	RPL35A	P18077
RPL36	Q9Y3U8	RPL38	P63173	RPL3L	Q92901
RPL4	P36578	RPL5	P46777	RPL7	P18124
RPL7A	P18124, P62424	RPL9P8	P32969	RPLP0	P05388
RPLP2	P05387	RPS10	P46783	RPS11	P62280
RPS12	P25398	RPS13	P62277	RPS14	P62263
RPS15	P62841	RPS15A	P62244	RPS16	P62249
RPS17	P08708	RPS18	P62269	RPS2	P15880, P46782
RPS23	P62266	RPS27	P42677, Q71UM5	RPS27A	P42677, P62979, P62987
RPS29	P62273	RPS3	P23396	RPS3A	P61247
RPS4X	P62701, Q8TD47	RPS5	P46782	RPS6	P62753
RPS8	P62241	RPS9	P46781	RPSA	P08865
SOS2	P42766				

9. SRP-dependent cotranslational protein targeting to membrane (R-HSA-1799339)

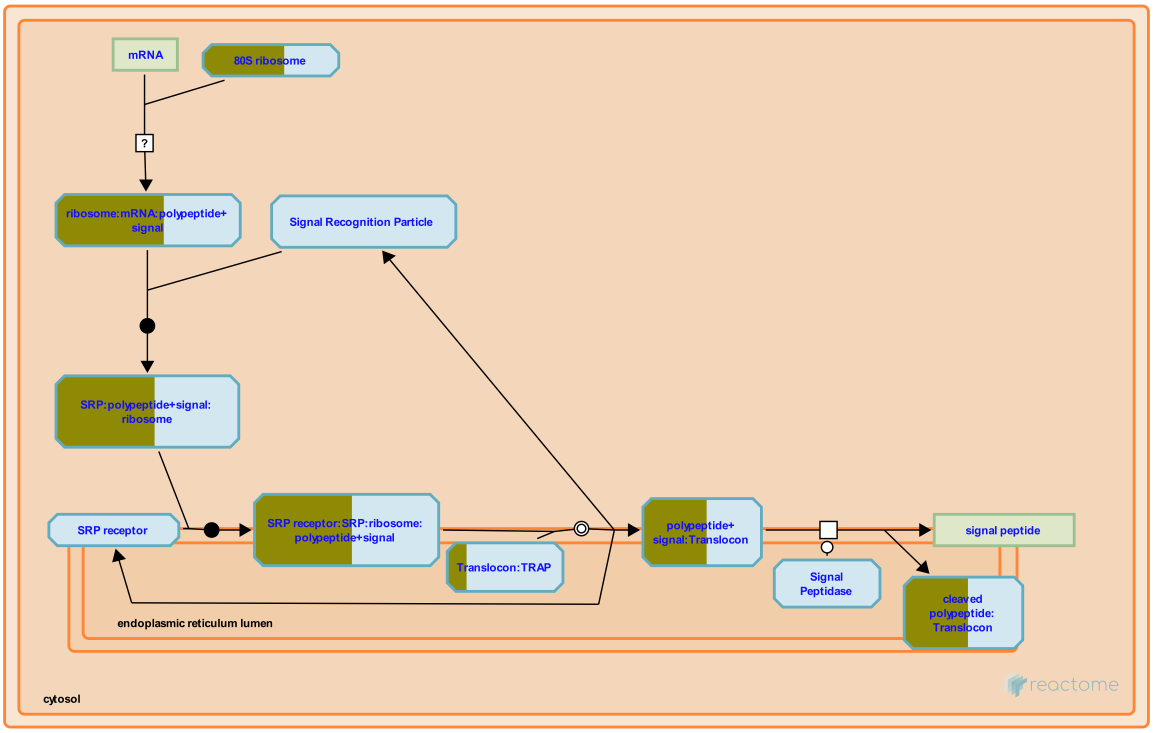

Cellular compartments: endoplasmic reticulum membrane, endoplasmic reticulum lumen, cytosol.

The process for translation of a protein destined for the endoplasmic reticulum (ER) branches from the canonical cytoslic translation process at the point when a nascent polypeptide containing a hydrophobic signal sequence is exposed on the surface of the cytosolic ribosome:mRNA:peptide complex. The signal sequence mediates the interaction of this complex with a cytosolic signal recognition particle (SRP) to form a complex which in turn docks with an SRP receptor complex on the ER membrane. There the ribosome complex is transferred from the SRP complex to a translocon complex embedded in the ER membrane and reoriented so that the nascent polypeptide protrudes through a pore in the translocon into the ER lumen. Translation, which had been halted by SRP binding, now resumes, the signal peptide is cleaved from the polypeptide, and elongation proceeds, with the growing polypeptide oriented into the ER lumen.

References

Edit history

Date	Action	Author
2008-11-20	Authored	May B, Gopinathrao G
2008-12-02	Reviewed	Matthews L, Gillespie ME, D'Eustachio P
2011-10-22	Revised	D'Eustachio P
2011-10-23	Edited	D'Eustachio P
2011-10-23	Created	D'Eustachio P
2023-08-31	Modified	Wright A

54 submitted entities found in this pathway, mapping to 68 Reactome entities

Input	UniProt Id	Input	UniProt Id	Input	UniProt Id
AGRN	P32969	FAU	P62861	RPL10	P27635, Q96L21
RPL10A	P61313, P62906	RPL13	P26373, P40429	RPL13A	P40429, P61313
RPL15	P61313	RPL18	Q07020	RPL21	P46778
RPL27A	P46776, P61353	RPL29	P47914	RPL3	P39023, Q92901
RPL30	P62888	RPL31	P62899	RPL32	P62888, P62910
RPL34	P49207, P62899	RPL35	P42766	RPL35A	P18077
RPL36	Q9Y3U8	RPL38	P63173	RPL3L	Q92901
RPL4	P36578	RPL5	P46777	RPL7	P18124
RPL7A	P18124, P62424	RPL9P8	P32969	RPLP0	P05388
RPLP2	P05387	RPN2	P04844	RPS10	P46783
RPS11	P62280	RPS12	P25398	RPS13	P62277
RPS14	P62263	RPS15	P62841	RPS15A	P62244
RPS16	P62249	RPS17	P08708	RPS18	P62269
RPS2	P15880, P46782	RPS23	P62266	RPS27	P42677, Q71UM5
RPS27A	P42677, P62979, P62987	RPS29	P62273	RPS3	P23396
RPS3A	P61247	RPS4X	P62701, Q8TD47	RPS5	P46782
RPS6	P62753	RPS8	P62241	RPS9	P46781
RPSA	P08865	SEC61G	P60059	SOS2	P42766

10. Interferon gamma signaling (R-HSA-877300)

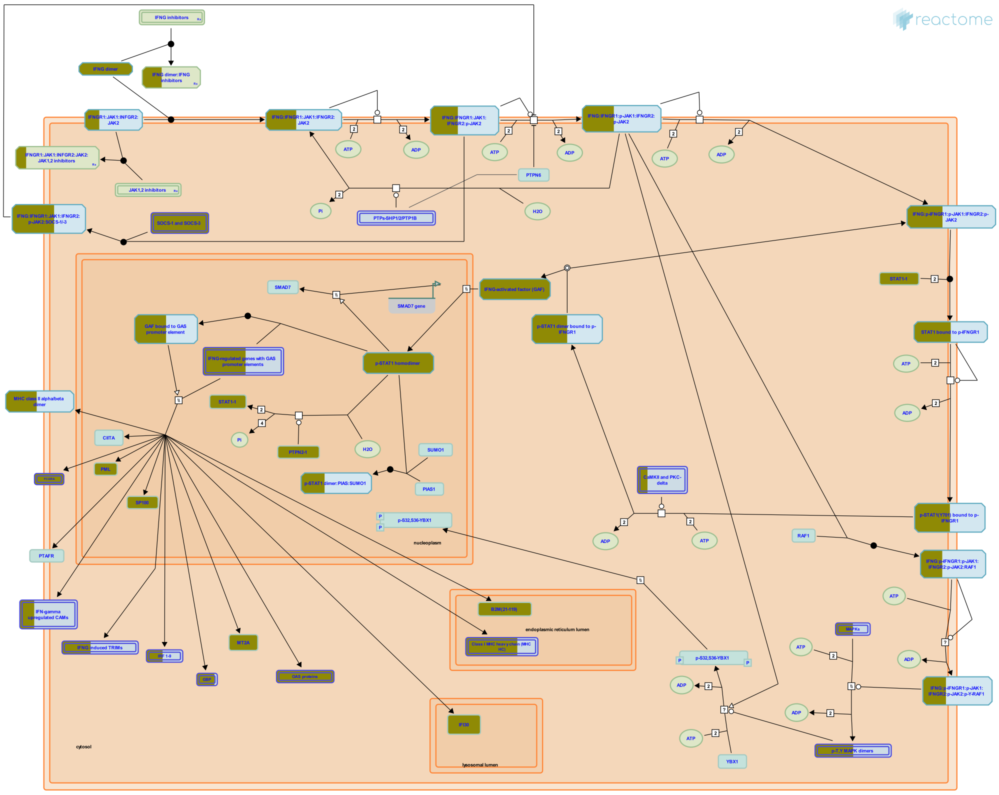

Interferon-gamma (IFN-gamma) belongs to the type II interferon family and is secreted by activated immune cells-primarily T and NK cells, but also B-cells and APC. INFG exerts its effect on cells by interacting with the specific IFN-gamma receptor (IFNGR). IFNGR consists of two chains, namely IFNGR1 (also known as the IFNGR alpha chain) and IFNGR2 (also known as the IFNGR beta chain). IFNGR1 is the ligand binding receptor and is required but not sufficient for signal transduction, whereas IFNGR2 do not bind IFNG independently but mainly plays a role in IFNG signaling and is generally the limiting factor in IFNG responsiveness. Both IFNGR chains lack intrinsic kinase/phosphatase activity and thus rely on other signaling proteins like Janus-activated kinase 1 (JAK1), JAK2 and Signal transducer and activator of transcription 1 (STAT-1) for signal transduction. IFNGR complex in its resting state is a preformed tetramer and upon IFNG association undergoes a conformational change. This conformational change induces the phosphorylation and activation of JAK1, JAK2, and STAT1 which in turn induces genes containing the gamma-interferon activation sequence (GAS) in the promoter.

References

Schroder K, Ravasi T, Hume DA & Hertzog PJ (2004). Interferon-gamma: an overview of signals, mechanisms and functions. J Leukoc Biol, 75, 163-89.

Aguet M, Bach EA & Schreiber RD (1997). The IFN gamma receptor: a paradigm for cytokine receptor signaling. Annu Rev Immunol, 15, 563-91.

Gough DJ, Levy DE, Clarke CJ & Johnstone RW (2008). IFNgamma signaling-does it mean JAK-STAT?. Cytokine Growth Factor Rev, 19, 383-94.

Izotova LS, Garotta G, Muthukumaran G, Kotenko SV, Cook JR & Pestka S (1997). The interferon gamma (IFN-gamma) receptor: a paradigm for the multichain cytokine receptor. Cytokine Growth Factor Rev, 8, 189-206.

Edit history

Date	Action	Author
2010-06-08	Edited	Garapati P V
2010-06-08	Authored	Garapati P V
2010-06-11	Created	Garapati P V
2010-08-17	Reviewed	Abdul-Sater AA, Schindler C
2023-08-31	Modified	Wright A

49 submitted entities found in this pathway, mapping to 89 Reactome entities

Input	UniProt Id	Input	UniProt Id	Input	UniProt Id
B2M	P61769	FCGR1A	P12314	FCGR1B	Q92637
GBP1	P32455	GBP2	P32456	GBP3	Q9H0R5
GBP4	Q96PP9	GBP5	Q96PP8	GBP6	Q6ZN66
HLA-A	P04439	HLA-B	P01889	HLA-C	P10321
HLA-DQA1	P01909, P20036	HLA-DQA2	P01906	HLA-DRA	P01903
HLA-DRB4	P13762	HLA-G	P17693	HLA-H	P01893
IFI30	P13284	IFNG	P01579	IRF1	P10914
IRF5	Q13568	IRF7	Q92985	IRF9	Q00978
JAK2	O60674	MAPK3	P27361	MT1A	P02795
MT2A	P02795	NCAM1	P13591	OAS1	P00973
OAS2	P29728	OAS3	Q9Y6K5	OASL	Q15646
PML	P29590	PRKCD	Q05655	PTPN2	P17706-2
SOCS1	O15524	SOCS3	O14543	SP100	P23497
SP110	P23497	STAT1	P42224-1	TRIM14	Q14142
TRIM21	P19474	TRIM22	Q8IYM9	TRIM25	Q14258
TRIM34	Q9BYJ4	TRIM38	O00635	TRIM5	Q9C035
TRIM6	Q9C030				

Input	Ensembl Id	Input	Ensembl Id	Input	Ensembl Id
B2M	ENSG00000166710	FCGR1A	ENSG00000150337	FCGR1B	ENSG00000198019
GBP1	ENSG00000117228	GBP2	ENSG00000162645	GBP3	ENSG00000117226
GBP4	ENSG00000162654	GBP5	ENSG00000154451	GBP6	ENSG00000183347
HLA-A	ENSG00000206503	HLA-B	ENSG00000234745	HLA-C	ENSG00000204525
HLA-DQA1	ENSG00000196735	HLA-DQA2	ENSG00000237541	HLA-DRA	ENSG00000204287
HLA-DRB4	ENSG00000227357	HLA-G	ENSG00000204632	HLA-H	ENSG00000206341
IFI30	ENSG00000216490	IRF1	ENSG00000125347	IRF5	ENSG00000128604
IRF7	ENSG00000185507	IRF9	ENSG00000213928	MT2A	ENSG00000125148
NCAM1	ENSG00000149294	OAS1	ENSG00000089127	OAS2	ENSG00000111335
OAS3	ENSG00000111331	OASL	ENSG00000135114	PML	ENSG00000140464
SP100	ENSG00000067066	TRIM14	ENSG00000106785	TRIM21	ENSG00000132109
TRIM22	ENSG00000132274	TRIM25	ENSG00000121060	TRIM34	ENSG00000258659
TRIM38	ENSG00000112343	TRIM5	ENSG00000132256	TRIM6	ENSG00000121236

11. Interferon alpha/beta signaling (R-HSA-909733)

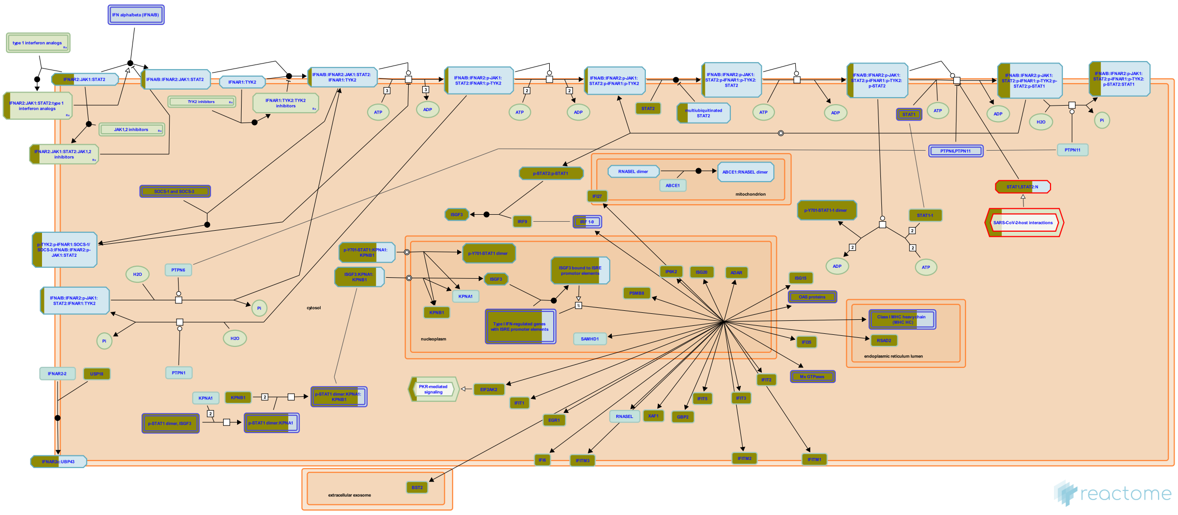

Type I interferons (IFNs) are composed of various genes including IFN alpha (IFNA), beta (IFNB), omega, epsilon, and kappa. In humans the IFNA genes are composed of more than 13 subfamily genes, whereas there is only one IFNB gene. The large family of IFNA/B proteins all bind to a single receptor which is composed of two distinct chains: IFNAR1 and IFNAR2. The IFNA/B stimulation of the IFNA receptor complex leads to the formation of two transcriptional activator complexes: IFNA-activated-factor (AAF), which is a homodimer of STAT1 and IFN-stimulated gene factor 3 (ISGF3), which comprises STAT1, STAT2 and a member of the IRF family, IRF9/P48. AAF mediates activation of the IRF-1 gene by binding to GAS (IFNG-activated site), whereas ISGF3 activates several IFN-inducible genes including IRF3 and IRF7.

References

Gupta S, Greenlund AC, Krolewski JJ, Yan H, Schreiber RD, Schindler CW, ... Krishnan K (1996). Phosphorylated interferon-alpha receptor 1 subunit (IFNaR1) acts as a docking site for the latent form of the 113 kDa STAT2 protein. EMBO J, 15, 1064-74.

Pellegrini S, Piehler J, Schreiber G & Uzé G (2007). The receptor of the type I interferon family. Curr Top Microbiol Immunol, 316, 71-95.

Gauzzi MC, Pellegrini S, Velazquez L, McKendry R, Fellous M & Mogensen KE (1996). Interferon-alpha-dependent activation of Tyk2 requires phosphorylation of positive regulatory tyrosines by another kinase. J Biol Chem, 271, 20494-500.

Stark GR, Darnell JE Jr, Qureshi S, Li X & Leung S (1996). Formation of STAT1-STAT2 heterodimers and their role in the activation of IRF-1 gene transcription by interferon-alpha. J Biol Chem, 271, 5790-4.

Edit history

Date	Action	Author
2010-07-07	Edited	Garapati P V
2010-07-07	Authored	Garapati P V
2010-07-07	Created	Garapati P V
2010-08-17	Reviewed	Abdul-Sater AA, Schindler C
2023-08-31	Modified	Wright A

43 submitted entities found in this pathway, mapping to 83 Reactome entities

Input	UniProt Id	Input	UniProt Id	Input	UniProt Id
ADAR	P55265	BST2	Q10589	EGR1	P18146
EIF2AK2	P19525	GBP2	P32456	HLA-A	P04439
HLA-B	P01889	HLA-C	P10321	HLA-G	P17693
HLA-H	P01893	IFI27	P40305	IFI35	P80217
IFI6	P09912	IFIT1	P09914	IFIT2	P09913
IFIT3	O14879	IFIT5	Q13325	IFITM1	P13164
IFITM2	Q01629	IFITM3	P13164, Q01628	IP6K2	Q9UHH9
IRF1	P10914	IRF5	Q13568	IRF7	Q92985
IRF9	Q00978	ISG15	P05161	ISG20	Q96AZ6
KPNB1	Q14974	MX1	P20591	MX2	P20592
OAS1	P00973	OAS2	P29728	OAS3	Q9Y6K5
OASL	Q15646	PSMB8	P28062	RPS27A	P62979, P62987
RSAD2	Q8WXG1	SOCS1	O15524	SOCS3	O14543
STAT1	P42224, P42224-1, P42224-2	STAT2	P52630	USP18	Q9UMW8
XAF1	Q6GPH4				

Input	Ensembl Id	Input	Ensembl Id	Input	Ensembl Id
ADAR	ENSG00000160710	BST2	ENSG00000130303	EGR1	ENSG00000120738
EIF2AK2	ENSG00000055332	GBP2	ENSG00000162645	HLA-A	ENSG00000206503
HLA-B	ENSG00000234745	HLA-C	ENSG00000204525	HLA-G	ENSG00000204632
HLA-H	ENSG00000206341	IFI27	ENSG00000165949	IFI35	ENSG00000068079
IFI6	ENSG00000126709	IFIT1	ENSG00000185745	IFIT2	ENSG00000119922
IFIT3	ENSG00000119917	IFIT5	ENSG00000152778	IFITM1	ENSG00000185885
IFITM2	ENSG00000185201	IFITM3	ENSG00000142089	IP6K2	ENSG00000068745
IRF1	ENSG00000125347	IRF5	ENSG00000128604	IRF7	ENSG00000185507
IRF9	ENSG00000213928	ISG15	ENSG00000187608	ISG20	ENSG00000172183
MX1	ENSG00000157601	MX2	ENSG00000183486	OAS1	ENSG00000089127
OAS2	ENSG00000111335	OAS3	ENSG00000111331	OASL	ENSG00000135114
PSMB8	ENSG00000204264	RSAD2	ENSG00000134321	XAF1	ENSG00000132530

12. Interferon Signaling (R-HSA-913531)

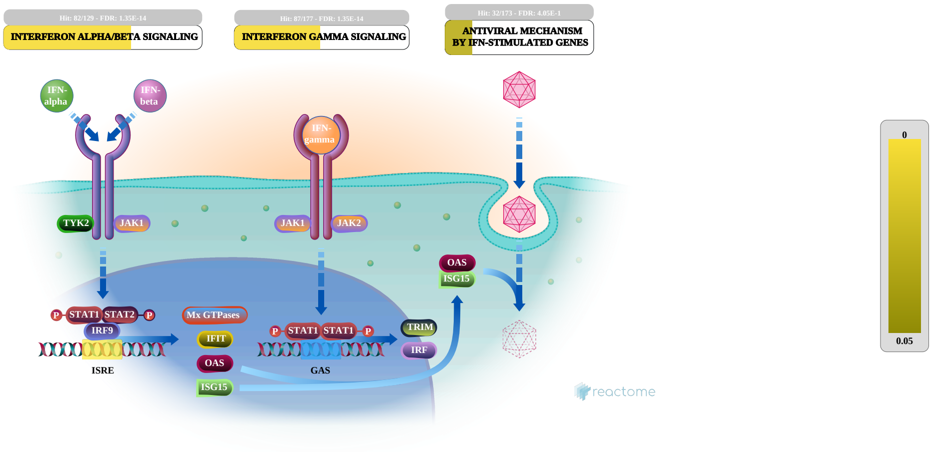

Interferons (IFNs) are cytokines that play a central role in initiating immune responses, especially antiviral and antitumor effects. There are three types of IFNs:Type I (IFN-alpha, -beta and others, such as omega, epsilon, and kappa), Type II (IFN-gamma) and Type III (IFN-lamda). In this module we are mainly focusing on type I IFNs alpha and beta and type II IFN-gamma. Both type I and type II IFNs exert their actions through cognate receptor complexes, IFNAR and IFNGR respectively, present on cell surface membranes. Type I IFNs are broadly expressed heterodimeric receptors composed of the IFNAR1 and IFNAR2 subunits, while the type II IFN receptor consists of IFNGR1 and IFNGR2. Type III interferon lambda has three members: lamda1 (IL-29), lambda2 (IL-28A), and lambda3 (IL-28B) respectively. IFN-lambda signaling is initiated through unique heterodimeric receptor composed of IFN-LR1/IF-28Ralpha and IL10R2 chains.

Type I IFNs typically recruit JAK1 and TYK2 proteins to transduce their signals to STAT1 and 2; in combination with IRF9 (IFN-regulatory factor 9), these proteins form the heterotrimeric complex ISGF3. In nucleus ISGF3 binds to IFN-stimulated response elements (ISRE) to promote gene induction.

Type II IFNs in turn rely upon the activation of JAKs 1 and 2 and STAT1. Once activated, STAT1 dimerizes to form the transcriptional regulator GAF (IFNG activated factor) and this binds to the IFNG activated sequence (GAS) elements and initiate the transcription of IFNG-responsive genes.

Like type I IFNs, IFN-lambda recruits TYK2 and JAK1 kinases and then promote the phosphorylation of STAT1/2, and induce the ISRE3 complex formation.

References

Schroder K, Ravasi T, Hume DA & Hertzog PJ (2004). Interferon-gamma: an overview of signals, mechanisms and functions. J Leukoc Biol, 75, 163-89.

Platanias LC (2005). Mechanisms of type-I- and type-II-interferon-mediated signalling. Nat Rev Immunol, 5, 375-86.

Gough DJ, Levy DE, Clarke CJ & Johnstone RW (2008). IFNgamma signaling-does it mean JAK-STAT?. Cytokine Growth Factor Rev, 19, 383-94.

Ferreira PC, Bonjardim CA & Kroon EG (2009). Interferons: signaling, antiviral and viral evasion. Immunol Lett, 122, 1-11.

Platanias LC & Uddin S (2004). Mechanisms of type-I interferon signal transduction. J Biochem Mol Biol, 37, 635-41.

Edit history

Date	Action	Author
2010-07-07	Edited	Garapati P V
2010-07-07	Authored	Garapati P V
2010-07-16	Created	Garapati P V
2010-08-17	Reviewed	Abdul-Sater AA, Schindler C
2023-08-26	Modified	Wright A

90 submitted entities found in this pathway, mapping to 158 Reactome entities

Input	UniProt Id	Input	UniProt Id	Input	UniProt Id
ADAR	P55265	B2M	P61769	BST2	Q10589
DDX58	O95786	EGR1	P18146	EIF2AK2	P19525
FAAP20	Q6NZ36	FANCA	O15360	FANCL	Q9NW38
FCGR1A	P12314	FCGR1B	Q92637	GBP1	P32455
GBP2	P32456	GBP3	Q9H0R5	GBP4	Q96PP9
GBP5	Q96PP8	GBP6	Q6ZN66	HERC5	Q9UII4
HLA-A	P04439	HLA-B	P01889	HLA-C	P10321
HLA-DQA1	P01909, P20036	HLA-DQA2	P01906	HLA-DRA	P01903
HLA-DRB4	P13762	HLA-G	P17693	HLA-H	P01893
HSPA1A	P0DMV8	IFI27	P40305	IFI30	P13284
IFI35	P80217	IFI6	P09912	IFIT1	P09914
IFIT2	P09913	IFIT3	O14879	IFIT5	Q13325
IFITM1	P13164	IFITM2	Q01629	IFITM3	P13164, Q01628
IFNG	P01579	IP6K2	Q9UHH9	IRF1	P10914
IRF5	Q13568	IRF7	Q92985	IRF9	Q00978
ISG15	P05161	ISG20	Q96AZ6	JAK2	O60674
KPNB1	Q14974	MAP2K6	P52564	MAPK3	P27361
MT1A	P02795	MT2A	P02795	MX1	P20591
MX2	P20592	NCAM1	P13591	NUP188	Q5SRE5
OAS1	P00973	OAS2	P29728	OAS3	Q9Y6K5
OASL	Q15646	PIN1	Q13526	PML	P29590
PRKCD	Q05655	PSMB8	P28062	PTPN2	P17706, P17706-2
RIGI	O95786	RPS27A	P62979, P62987	RSAD2	Q8WXG1
SOCS1	O15524	SOCS3	O14543	SP100	P23497
SP110	P23497	STAT1	P42224, P42224-1, P42224-2	STAT2	P52630
TARBP2	Q15633	TRIM14	Q14142	TRIM21	P19474
TRIM22	Q8IYM9	TRIM25	Q14258	TRIM34	Q9BYJ4
TRIM38	O00635	TRIM5	Q9C035	TRIM6	Q9C030
TUBA4A	P68366	UBA7	P41226	UBE2L6	O14933
USP18	Q3LFD5, Q9UMW8	USP41	Q3LFD5	XAF1	Q6GPH4

Input	Ensembl Id	Input	Ensembl Id	Input	Ensembl Id
ADAR	ENSG00000160710	B2M	ENSG00000166710	BST2	ENSG00000130303
EGR1	ENSG00000120738	EIF2AK2	ENSG00000055332	FCGR1A	ENSG00000150337
FCGR1B	ENSG00000198019	GBP1	ENSG00000117228	GBP2	ENSG00000162645
GBP3	ENSG00000117226	GBP4	ENSG00000162654	GBP5	ENSG00000154451
GBP6	ENSG00000183347	HLA-A	ENSG00000206503	HLA-B	ENSG00000234745
HLA-C	ENSG00000204525	HLA-DQA1	ENSG00000196735	HLA-DQA2	ENSG00000237541
HLA-DRA	ENSG00000204287	HLA-DRB4	ENSG00000227357	HLA-G	ENSG00000204632
HLA-H	ENSG00000206341	IFI27	ENSG00000165949	IFI30	ENSG00000216490
IFI35	ENSG00000068079	IFI6	ENSG00000126709	IFIT1	ENSG00000185745
IFIT2	ENSG00000119922	IFIT3	ENSG00000119917	IFIT5	ENSG00000152778
IFITM1	ENSG00000185885	IFITM2	ENSG00000185201	IFITM3	ENSG00000142089
IP6K2	ENSG00000068745	IRF1	ENSG00000125347	IRF5	ENSG00000128604
IRF7	ENSG00000185507	IRF9	ENSG00000213928	ISG15	ENSG00000187608
ISG20	ENSG00000172183	MT2A	ENSG00000125148	MX1	ENSG00000157601
MX2	ENSG00000183486	NCAM1	ENSG00000149294	OAS1	ENSG00000089127
OAS2	ENSG00000111335	OAS3	ENSG00000111331	OASL	ENSG00000135114
PML	ENSG00000140464	PSMB8	ENSG00000204264	RSAD2	ENSG00000134321
SP100	ENSG00000067066	TRIM14	ENSG00000106785	TRIM21	ENSG00000132109
TRIM22	ENSG00000132274	TRIM25	ENSG00000121060	TRIM34	ENSG00000258659
TRIM38	ENSG00000112343	TRIM5	ENSG00000132256	TRIM6	ENSG00000121236
XAF1	ENSG00000132530				

13. Cytokine Signaling in Immune system (R-HSA-1280215)

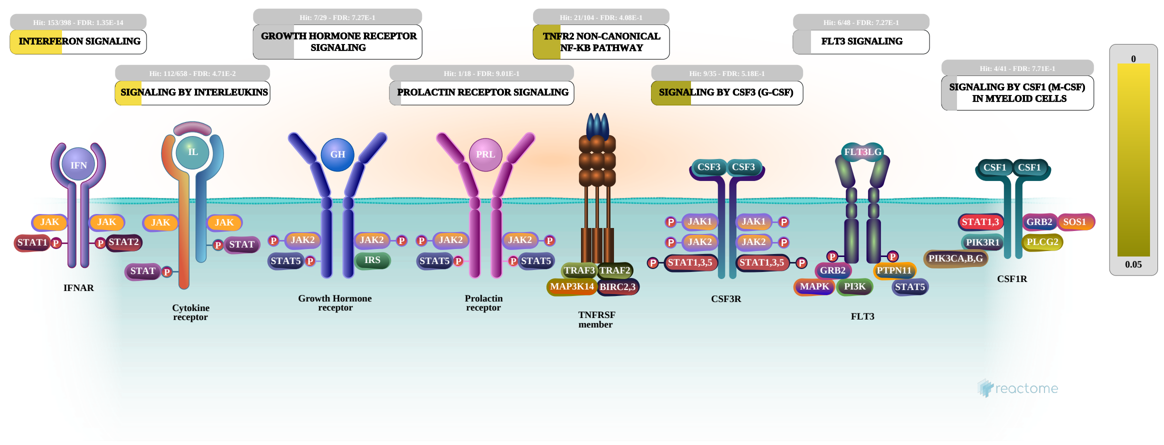

Cytokines are small proteins that regulate and mediate immunity, inflammation, and hematopoiesis. They are secreted in response to immune stimuli, and usually act briefly, locally, at very low concentrations. Cytokines bind to specific membrane receptors, which then signal the cell via second messengers, to regulate cellular activity.

References

Feldmann M & Oppenheim J (2002). *Cytokines and the immune system, Cytokine Reference* .

IMMPORT:Bioinformatics for the future of immunology. Retrieved from https://www.immport.org/immportWeb/queryref/geneListSummary.do

Santamaria P (2003). Cytokines and chemokines in autoimmune disease: an overview. Adv Exp Med Biol, 520, 1-7.

COPE. Retrieved from http://www.copewithcytokines.org/cope.cgi

Edit history

Date	Action	Author
2011-05-12	Created	Garapati P V
2011-05-22	Edited	Ray KP, Jupe S, Garapati P V
2011-05-22	Authored	Ray KP, Jupe S, Garapati P V
2011-05-29	Reviewed	Abdul-Sater AA, Schindler C, Pinteaux E
2023-08-26	Modified	Wright A

163 submitted entities found in this pathway, mapping to 272 Reactome entities

Input	UniProt Id	Input	UniProt Id	Input	UniProt Id
ADAR	P55265	ALOX15	P16050	ARF1	P84077
B2M	P61769	BIRC2	Q13489, Q13490	BST2	Q10589
CASP1	P29466	CCL2	P13500	CCL20	P78556

Input	UniProt Id	Input	UniProt Id	Input	UniProt Id
CCR1	P32246	CD27	P26842	CD40LG	P29965
CDKN1A	P38936	CISH	Q9NSE2	CXCL10	P02778
DDX58	O95786	DUSP6	Q16828	EDAR	Q9UNE0
EGR1	P18146	EIF2AK2	P19525	FAAP20	Q6NZ36
FANCA	O15360	FANCL	Q9NW38	FBXW11	Q9UKB1
FCGR1A	P12314	FCGR1B	Q92637	FLT3LG	P49771
GBP1	P32455	GBP2	P32456	GBP3	Q9H0R5
GBP4	Q96PP9	GBP5	Q96PP8	GBP6	Q6ZN66
H3C4	P68431, Q71DI3	HERC5	Q9UII4	HLA-A	P04439
HLA-B	P01889	HLA-C	P10321	HLA-DQA1	P01909, P20036
HLA-DQA2	P01906	HLA-DRA	P01903	HLA-DRB4	P13762
HLA-G	P17693	HLA-H	P01893	HNRNPA2B1	P22626
HSPA1A	P0DMV8	IFI27	P40305	IFI30	P13284
IFI35	P80217	IFI6	P09912	IFIT1	P09914
IFIT2	P09913	IFIT3	O14879	IFIT5	Q13325
IFITM1	P13164	IFITM2	Q01629	IFITM3	P13164, Q01628
IFNG	P01579	IGHE	P01854	IGHG1	P01857, P01861
IGHG4	P01861	IKBIP	Q70UQ0	IL11RA	P08887, Q14626
IL15	P40933	IL1RN	P18510	IL27	Q8NEV9
IL2RB	P14784	IL37	Q9NZH6	IL4R	P24394, Q01113
IL5RA	P15509, Q01344	IL6	P05231	IL6R	P08887, P08887-2
IL7R	P16871	IP6K2	Q9UHH9	IRF1	P10914
IRF5	Q13568	IRF7	Q92985	IRF9	Q00978
ISG15	P05161	ISG20	Q96AZ6	ITGB1	P05556
JAK2	O60674	KPNB1	Q14974	LGALS9	O00182
LIF	P15018	LMNB1	P20700	LTB	Q06643
MAP2K3	P46734	MAP2K6	P52564	MAPK3	P27361
MEF2C	Q06413	MT1A	P02795	MT2A	P02795
MUC1	P15941	MX1	P20591	MX2	P20592
MYD88	Q99836	NCAM1	P13591	NLRC5	Q86WI3
NLRX1	Q86UT6	NOD2	Q9HC29	NUP188	Q5SRE5
OAS1	P00973	OAS2	P29728	OAS3	Q9Y6K5
OASL	Q15646	PIK3CA	P42336	PIN1	Q13526
PML	P29590	PPIA	P62937	PPP2R5D	Q14738
PRKCD	Q05655	PSMB8	P28062	PSMB9	P28065
PSME1	Q06323	PSME2	Q9UL46	PTK2B	Q14289
PTPN2	P17706, P17706-2	REL	Q01201	RIGI	O95786
RORA	P35398	RORC	P51449	RPLP0	P05388
RPN2	Q99460	RPS27A	P62979, P62987	RSAD2	Q8WXG1
S100B	P04271	SIGIRR	Q6IA17	SOCS1	O15524
SOCS2	O14508	SOCS3	O14543	SOS2	Q07890
SP100	P23497	SP110	P23497	SQSTM1	Q13501
STAT1	P42224, P42224-1, P42224-2	STAT2	P52630	SYK	P43405
TAB3	Q8N5C8	TARBP2	Q15633	TIFA	Q96CG3
TNFRSF17	Q02223	TNFRSF25	Q93038	TNFSF13	O75888
TNFSF13B	Q9Y275	TNFSF14	O43557	TRAF3	Q13114
TRIM14	Q14142	TRIM21	P19474	TRIM22	Q8IYM9
TRIM25	Q14258	TRIM34	Q9BYJ4	TRIM38	O00635
TRIM5	Q9C035	TRIM6	Q9C030	TUBA4A	P68366

Input	UniProt Id	Input	UniProt Id	Input	UniProt Id
UBA7	P41226	UBE2L6	O14933	USP18	Q3LFD5, Q9UMW8
USP41	Q3LFD5	VIM	P08670	VRK3	Q8IV63
XAF1	Q6GPH4				

Input	Ensembl Id	Input	Ensembl Id	Input	Ensembl Id
ADAR	ENSG00000160710	ALOX15	ENSG00000161905	ARF1	ENSG00000143761
B2M	ENSG00000166710	BST2	ENSG00000130303	CCL2	ENSG00000108691
CCL20	ENSG00000115009	CCR1	ENSG00000163823	CDKN1A	ENSG00000124762
CISH	ENSG00000114737	CXCL10	ENSG00000169245	EGR1	ENSG00000120738
EIF2AK2	ENSG00000055332	FCGR1A	ENSG00000150337	FCGR1B	ENSG00000198019
GBP1	ENSG00000117228	GBP2	ENSG00000162645	GBP3	ENSG00000117226
GBP4	ENSG00000162654	GBP5	ENSG00000154451	GBP6	ENSG00000183347
HLA-A	ENSG00000206503	HLA-B	ENSG00000234745	HLA-C	ENSG00000204525
HLA-DQA1	ENSG00000196735	HLA-DQA2	ENSG00000237541	HLA-DRA	ENSG00000204287
HLA-DRB4	ENSG00000227357	HLA-G	ENSG00000204632	HLA-H	ENSG00000206341
HNRNPA2B1	ENSG00000122566	IFI27	ENSG00000165949	IFI30	ENSG00000216490
IFI35	ENSG00000068079	IFI6	ENSG00000126709	IFIT1	ENSG00000185745
IFIT2	ENSG00000119922	IFIT3	ENSG00000119917	IFIT5	ENSG00000152778
IFITM1	ENSG00000185885	IFITM2	ENSG00000185201	IFITM3	ENSG00000142089
IFNG	ENSG00000111537	IGHE	ENSG00000211891	IGHG1	ENSG00000211896
IGHG4	ENSG00000211892	IKBIP	ENSG00000166130	IL1RN	ENSG00000136689
IL4R	ENSG00000077238	IL6	ENSG00000136244	IL6R	ENSG00000160712
IP6K2	ENSG00000068745	IRF1	ENSG00000125347	IRF5	ENSG00000128604
IRF7	ENSG00000185507	IRF9	ENSG00000213928	ISG15	ENSG00000187608
ISG20	ENSG00000172183	ITGB1	ENSG00000150093	LIF	ENSG00000128342
LMNB1	ENSG00000113368	MT2A	ENSG00000125148	MUC1	ENSG00000185499
MX1	ENSG00000157601	MX2	ENSG00000183486	NCAM1	ENSG00000149294
OAS1	ENSG00000089127	OAS2	ENSG00000111335	OAS3	ENSG00000111331
OASL	ENSG00000135114	PML	ENSG00000140464	PPIA	ENSG00000196262
PSMB8	ENSG00000204264	PSME2	ENSG00000100911	PTPN2	ENSG00000175354
RORA	ENSG00000069667	RORC	ENSG00000143365	RPLP0	ENSG00000089157
RSAD2	ENSG00000134321	SOCS1	ENSG00000185338	SOCS2	ENSG00000120833
SOCS3	ENSG00000184557, ENST00000330871	SP100	ENSG00000067066	STAT1	ENSG00000115415
TRIM14	ENSG00000106785	TRIM21	ENSG00000132109	TRIM22	ENSG00000132274
TRIM25	ENSG00000121060	TRIM34	ENSG00000258659	TRIM38	ENSG00000112343
TRIM5	ENSG00000132256	TRIM6	ENSG00000121236	VIM	ENSG00000026025
XAF1	ENSG00000132530				

14. Immune System (R-HSA-168256)

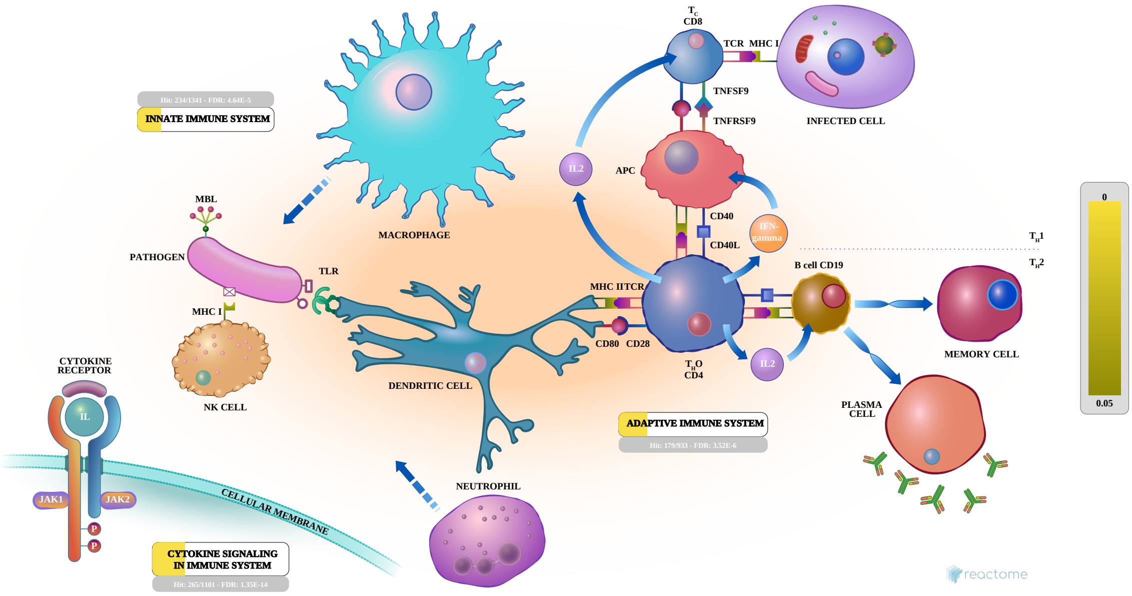

Humans are exposed to millions of potential pathogens daily, through contact, ingestion, and inhalation. Our ability to avoid infection depends on the adaptive immune system and during the first critical hours and days of exposure to a new pathogen, our innate immune system.

References

Edit history

Date	Action	Author
2005-11-12	Created	Gillespie ME
2006-03-30	Authored	Luo F, Ouwehand WH, Gillespie ME, de Bono B
2006-04-19	Reviewed	Zwaginga JJ, D'Eustachio P, Gay NJ, Gale M Jr
2023-08-26	Modified	Wright A

406 submitted entities found in this pathway, mapping to 537 Reactome entities

Input	UniProt Id	Input	UniProt Id	Input	UniProt Id
ACTR3	P61158	ADA2	Q9NZK5	ADAR	P55265
ADGRE3	Q9BY15	ADGRG1	Q86Y34	AIM2	O14862
ALDOA	P04075	ALDOC	P09972	ALOX15	P16050
ANPEP	P15144	AP1B1	Q10567	ARF1	P84077
ARPC1B	O15143	ATP6V0E2	Q8NHE4	B2M	P28068, P61769
BAIAP2	Q9UQB8	BIRC2	Q13489, Q13490	BRI3	O95415
BST1	Q10588	BST2	Q10589	BTF3	O00478, P78410
BTK	Q06187	BTN2A2	Q8WVV5, Q9UIR0	BTN3A1	O00481
BTNL8	Q6UX41	C1QA	P02745	C1QB	P02746
C1QC	P02747	C2	P06681	C3AR1	Q16581
C5	P01031	C5AR2	Q9P296	CASP1	P29466
CASP4	P49662	CAT	P04040	CCL2	P13500
CCL20	P78556	CCR1	P32246	CCR6	P51684

Input	UniProt Id	Input	UniProt Id	Input	UniProt Id
CD160	O95971	CD1C	P29017	CD22	P20273
CD247	P20963-1	CD27	P26842	CD274	Q9NZQ7
CD300E	Q496F6	CD34	P28906-1	CD40LG	P29965
CD68	P34810	CD8B	P10966	CD96	P40200
CDKN1A	P38936	CEACAM1	P13688	CFB	P00751
CFP	P27918	CISH	Q9NSE2	CKAP4	Q07065
CLEC10A	Q6UW15, Q8IUN9	CLEC12A	Q5QGZ9	CLEC2B	Q92478
CLEC4D	Q8WXI8	CLEC4E	Q9UJ71, Q9ULY5	CLEC7A	Q9BXN2
CLTC	Q00610, Q00610-1	CTSF	Q9UBX1	CTSL	O60911, P07711
CXCL10	P02778	CXCR2	P25024, P25025	CYBB	P04839
CYFIP2	Q96F07	DAPP1	Q9UN19	DBNL	Q9UJU6
DBP	Q03518	DDX58	O95786	DHX58	Q96C10
DNASE1L3	P49184	DSC1	Q08554	DTX3L	Q8TDB6
DUSP6	Q16828	DYNLT1	P63172	EDAR	Q9UNE0
EEF1A1	P68104	EEF2	P13639	EGR1	P18146
EIF2AK2	P19525	FAAP20	Q6NZ36	FANCA	O15360
FANCL	Q9NW38	FBXL8	Q96CD0	FBXO30	Q8TB52
FBXO6	Q9NRD1	FBXO7	Q9Y3I1	FBXO9	Q9UK97
FBXW11	Q9UKB1	FCAR	P24071	FCER1A	P12319
FCGR1A	P12314	FCGR1B	Q92637	FCGR2A	P12318
FCGR3A	P08637	FCGR3B	O75015	FCN1	O00602
FGL2	Q14314	FLT3LG	P49771	FTH1	P02794
GBP1	P32455	GBP2	P32456	GBP3	Q9H0R5
GBP4	Q96PP9	GBP5	Q96PP8	GBP6	Q6ZN66
GDI2	P50395	GM2A	P17900	GNLY	P22749
GOLGA7	Q7Z5G4	GPR84	Q9NQS5	GRN	P28799
GYG1	P46976	GZMM	P51124	H3C4	P68431, Q71DI3
HERC4	Q15034, Q5GLZ8	HERC5	Q9UII4	HERC6	Q8IVU3
HK3	P52790	HLA-A	P04439	HLA-B	P01889
HLA-C	P10321	HLA-DQA1	P01909, P20036	HLA-DQA2	P01906
HLA-DRA	P01903	HLA-DRB4	P13762	HLA-G	P17693
HLA-H	P01893	HNRNPA2B1	P22626	HP	P00738
HSPA1A	P0DMV8	HSPA6	P17066	ICOSLG	O75144
IFI16	Q16666	IFI27	P40305	IFI30	P13284
IFI35	P80217	IFI6	P09912	IFIH1	Q9BYX4
IFIT1	P09914	IFIT2	P09913	IFIT3	O14879
IFIT5	Q13325	IFITM1	P13164	IFITM2	Q01629
IFITM3	P13164, Q01628	IFNG	P01579	IGHE	P01854
IGHG1	P01857, P01861	IGHG3	P01860	IGHG4	P01861
IGHV1-69	P01742	IGHV3-11	P01762	IGHV3-23	P01764
IGHV3-30	P01768	IGHV3-33	P01772	IGHV4-34	P06331
IGHV4-39	P01824	IGHV4-59	P01825	IGKC	P01834
IGKV1-12	A0A0C4DH73	IGKV1-17	P01599	IGKV1-33	P01594
IGKV1-5	P01602	IGKV1D-39	P04432	IGKV3-11	P04433
IGKV3-15	P01624	IGKV3-20	P01619	IGLC1	P0CG04
IGLC2	P0DOY2	IGLC3	P0DOY3	IGLC7	A0M8Q6
IGLV1-40	P01703, Q5NV69	IGLV1-44	P01699, Q5NV81	IGLV1-47	P01700
IGLV1-51	P01701	IGLV2-14	P01704	IGLV2-23	P01705, Q5NV89
IGLV3-1	P01715	IGLV3-19	P01714	IGLV3-21	P80748

Input	UniProt Id	Input	UniProt Id	Input	UniProt Id
IGLV3-25	P01717, Q5NV90	IGLV6-57	P01721	IKBIP	Q70UQ0
IL11RA	P08887, Q14626	IL15	P40933	IL1RN	P18510
IL27	Q8NEV9	IL2RB	P14784	IL37	Q9NZH6
IL4R	P24394, Q01113	IL5RA	P15509, Q01344	IL6	P05231
IL6R	P08887, P08887-2	IL7R	P16871	IMPDH1	P20839
IP6K2	Q9UHH9	IQGAP1	P46940	IRF1	P10914
IRF5	Q13568	IRF7	Q92985	IRF9	Q00978
ISG15	P05161	ISG20	Q96AZ6	ITGA4	P13612
ITGB1	P05556	JAK2	O60674	JUP	P14923
KIR2DL1	P43626, P43629	KIR3DL1	P43629	KLHL3	Q9UH77
KLRB1	Q12918	KLRF1	Q9NZS2	KLRG1	Q96E93
KPNB1	Q14974	KRT1	P04264	LAG3	P18627
LAMP3	P34810	LAMTOR1	Q6IAA8	LAP3	Q13867
LGALS9	O00182	LGMN	Q99538	LIF	P15018
LILRA4	P59901	LILRA5	A6NI73, Q8N149	LILRA6	Q6PI73
LILRB1	Q8NHL6	LILRB2	Q8N423	LILRB4	Q8N149, Q8NHJ6
LMNB1	P20700	LTB	Q06643	LY96	Q9Y6Y9
MAL	P01732, P58753	MAP2K3	P46734	MAP2K6	P52564
MAP3K1	Q13233	MAPK3	P27361	MBP	P13727
MEF2C	Q06413	MEFV	O15553	MICB	Q29980
MMP8	P22894	MNDA	P41218	MT1A	P02795, P53803
MT2A	P02795	MUC1	P15941	MUC6	Q6W4X9
MX1	P20591	MX2	P20592	MYD88	Q99836
NAPA	Q9Y2A7	NCAM1	P13591	NCF1	P14598
NCF2	P19878	NCR3	O14931	NHLRC3	Q5JS37
NLRC5	Q86WI3	NLRX1	Q86UT6	NMUR1	O94822
NOD2	Q9HC29	NPC2	P61916	NPDC1	Q9NQX5
NUP188	Q5SRE5	OAS1	P00973	OAS2	P29728
OAS3	Q9Y6K5	OASL	Q15646	P2RX1	P51575
PDCD1LG2	Q9BQ51	PDPK1	O15530	PGAM1	P18669
PI3	P19957	PIK3AP1	Q6ZUJ8	PIK3CA	P42336
PILRA	Q9UKJ1	PIN1	Q13526	PJA1	Q8NG27
PLAUR	Q03405	PLPP5	Q8NEB5	PML	P29590
POLR1E	O15160	POLR3H	Q9Y535	PPBP	P02775
PPIA	P62937, Q9UNP9	PPP2R5D	Q14738	PPP3CA	Q08209
PPP3CB	P16298	PPP3CC	P16298	PPP3R1	P63098
PRKCB	P05771	PRKCD	Q05655	PRKCE	Q02156
PRKCSH	P14314	PRKDC	P78527	PRR5	P85299
PSMB8	P28062	PSMB9	P28065	PSME1	Q06323
PSME2	Q9UL46	PTK2B	Q14289	PTPN2	P17706, P17706-2
PTPN22	Q9Y2R2	PTPRN2	Q92932	RAB24	Q969Q5
RAB3D	O95716	RBCK1	Q9BYM8	REG4	Q06141
REL	Q01201, Q04864	RIGI	O95786	RLIM	Q9NVW2
RNASE2	P10153	RNF213	Q63HN8	RNF38	Q9P000
RORA	P35398	RORC	P51449	RPLP0	P05388
RPN2	Q99460	RPS27A	P62979, P62987	RSAD2	Q8WXG1
S100B	P04271	SDCBP	O00560	SEC24D	O94855
SEC61G	P60059	SELL	P14151	SERPING1	P05155
SH2D1B	O14796	SIGIRR	Q6IA17	SIGLEC1	Q9BZZ2

Input	UniProt Id	Input	UniProt Id	Input	UniProt Id
SIGLEC14	Q08ET2	SIGLEC5	O15389, O43699	SIGLEC7	Q9Y286
SIGLEC8	Q96PQ1, Q9NYZ4	SIRPA	P78324	SLC15A4	Q8N697
SLC27A3	O14975	SOCS1	O15524	SOCS2	O14508
SOCS3	O14543	SOS2	Q07890	SP100	P23497
SP110	P23497	SPSB2	Q99619	SQSTM1	Q13501
STAT1	P42224, P42224-1, P42224-2	STAT2	P52630	SYK	P43405
TAB3	Q8N5C8	TAP1	Q03518	TAP2	Q03519
TARBP2	Q15633	TCN1	P20061	TIFA	Q96CG3
TIMP2	P16035	TLR1	Q15399	TLR2	O60603
TLR4	O00206	TLR5	O60602	TLR7	Q9NYK1
TLR8	Q9NR97	TLR9	Q9NR96	TNFAIP6	P98066
TNFRSF17	Q02223	TNFRSF25	Q93038	TNFSF13	O75888
TNFSF13B	Q9Y275	TNFSF14	O43557	TOM1L2	O60784
TRAF3	Q13114	TREML1	Q86YW5	TREX1	Q9NSU2
TRIM14	Q14142	TRIM21	P19474	TRIM22	Q8IYM9
TRIM25	Q14258	TRIM34	Q9BYJ4	TRIM38	O00635
TRIM5	Q9C035	TRIM56	Q9BRZ2	TRIM6	Q9C030
TRIM69	Q86WT6	TRIP12	Q14669	TUBA4A	P68366
TXK	P42681	TXNDC5	Q8NBS9	UBA6	A0AVT1
UBA7	P41226	UBE2C	O00762	UBE2G1	P62253
UBE2J2	Q8N2K1	UBE2L6	O14933	UBOX5	O94941
UFL1	O94874	UNC93B1	Q9H1C4	USP18	Q3LFD5, Q9UMW8
USP41	Q3LFD5	VIM	P08670	VRK3	Q8IV63
WIPF1	O43516	WSB1	Q9Y6I7	XAF1	Q6GPH4
ZBP1	Q9H171				

Input	Ensembl Id	Input	Ensembl Id	Input	Ensembl Id
ADAR	ENSG00000160710	ALOX15	ENSG00000161905	ARF1	ENSG00000143761
B2M	ENSG00000166710	BST2	ENSG00000130303	CCL2	ENSG00000108691
CCL20	ENSG00000115009	CCR1	ENSG00000163823	CDKN1A	ENSG00000124762
CISH	ENSG00000114737	CXCL10	ENSG00000169245	EGR1	ENSG00000120738
EIF2AK2	ENSG00000055332	FCGR1A	ENSG00000150337	FCGR1B	ENSG00000198019
GBP1	ENSG00000117228	GBP2	ENSG00000162645	GBP3	ENSG00000117226
GBP4	ENSG00000162654	GBP5	ENSG00000154451	GBP6	ENSG00000183347
HLA-A	ENSG00000206503	HLA-B	ENSG00000234745	HLA-C	ENSG00000204525
HLA-DQA1	ENSG00000196735	HLA-DQA2	ENSG00000237541	HLA-DRA	ENSG00000204287
HLA-DRB4	ENSG00000227357	HLA-G	ENSG00000204632	HLA-H	ENSG00000206341
HNRNPA2B1	ENSG00000122566	IFI27	ENSG00000165949	IFI30	ENSG00000216490
IFI35	ENSG00000068079	IFI6	ENSG00000126709	IFIT1	ENSG00000185745
IFIT2	ENSG00000119922	IFIT3	ENSG00000119917	IFIT5	ENSG00000152778
IFITM1	ENSG00000185885	IFITM2	ENSG00000185201	IFITM3	ENSG00000142089
IFNG	ENSG00000111537	IGHE	ENSG00000211891	IGHG1	ENSG00000211896
IGHG4	ENSG00000211892	IKBIP	ENSG00000166130	IL1RN	ENSG00000136689
IL4R	ENSG00000077238	IL6	ENSG00000136244	IL6R	ENSG00000160712
IP6K2	ENSG00000068745	IRF1	ENSG00000125347	IRF5	ENSG00000128604
IRF7	ENSG00000185507	IRF9	ENSG00000213928	ISG15	ENSG00000187608
ISG20	ENSG00000172183	ITGB1	ENSG00000150093	LIF	ENSG00000128342
LMNB1	ENSG00000113368	MT2A	ENSG00000125148	MUC1	ENSG00000185499
MX1	ENSG00000157601	MX2	ENSG00000183486	NCAM1	ENSG00000149294

Input	Ensembl Id	Input	Ensembl Id	Input	Ensembl Id
OAS1	ENSG00000089127	OAS2	ENSG00000111335	OAS3	ENSG00000111331
OASL	ENSG00000135114	PML	ENSG00000140464	PPIA	ENSG00000196262
PSMB8	ENSG00000204264	PSME2	ENSG00000100911	PTPN2	ENSG00000175354
RORA	ENSG00000069667	RORC	ENSG00000143365	RPLP0	ENSG00000089157
RSAD2	ENSG00000134321	SOCS1	ENSG00000185338	SOCS2	ENSG00000120833
SOCS3	ENSG00000184557, ENST00000330871	SP100	ENSG00000067066	STAT1	ENSG00000115415
TRIM14	ENSG00000106785	TRIM21	ENSG00000132109	TRIM22	ENSG00000132274
TRIM25	ENSG00000121060	TRIM34	ENSG00000258659	TRIM38	ENSG00000112343
TRIM5	ENSG00000132256	TRIM6	ENSG00000121236	VIM	ENSG00000026025
XAF1	ENSG00000132530				

15. Eukaryotic Translation Termination (R-HSA-72764)

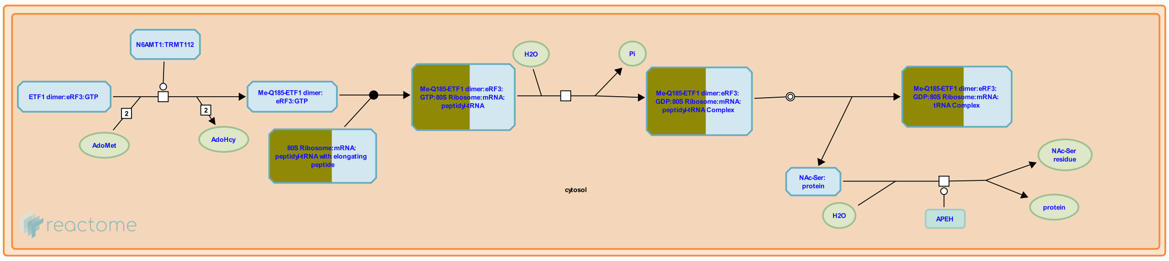

Cellular compartments: cytosol.

The arrival of any of the three stop codons (UAA, UAG and UGA) into the ribosomal A-site triggers the binding of a release factor (RF) to the ribosome and subsequent polypeptide chain release. In eukaryotes, the RF is composed of two proteins, eRF1 and eRF3. eRF1 is responsible for the hydrolysis of the peptidyl-tRNA, while eRF3 provides a GTP-dependent function. The ribosome releases the mRNA and dissociates into its two complex subunits, which can reassemble on another molecule to begin a new round of protein synthesis. It should be noted that at present, there is no factor identified in eukaryotes that would be the functional equivalent of the bacterial ribosome release (or recycling) factor, RRF, that catalyzes dissociation of the ribosome from the mRNA following release of the polypeptide

References

Salas-Marco J & Bedwell DM (2004). GTP hydrolysis by eRF3 facilitates stop codon decoding during eukaryotic translation termination. Mol Cell Biol, 24, 7769-78.

Ichikawa S & Kaji A (1989). Molecular cloning and expression of ribosome releasing factor. J Biol Chem, 264, 20054-9.

Edit history

Date	Action	Author
2004-11-09	Authored	Bedwell DM
2005-01-03	Created	Merrick WC, Bedwell DM
2023-08-24	Edited	Gillespie ME
2023-08-31	Modified	Wright A

52 submitted entities found in this pathway, mapping to 66 Reactome entities

Input	UniProt Id	Input	UniProt Id	Input	UniProt Id
AGRN	P32969	FAU	P62861	RPL10	P27635, Q96L21
RPL10A	P61313, P62906	RPL13	P26373, P40429	RPL13A	P40429, P61313
RPL15	P61313	RPL18	Q07020	RPL21	P46778
RPL27A	P46776, P61353	RPL29	P47914	RPL3	P39023, Q92901
RPL30	P62888	RPL31	P62899	RPL32	P62888, P62910
RPL34	P49207, P62899	RPL35	P42766	RPL35A	P18077
RPL36	Q9Y3U8	RPL38	P63173	RPL3L	Q92901
RPL4	P36578	RPL5	P46777	RPL7	P18124
RPL7A	P18124, P62424	RPL9P8	P32969	RPLP0	P05388
RPLP2	P05387	RPS10	P46783	RPS11	P62280

Input	UniProt Id	Input	UniProt Id	Input	UniProt Id
RPS12	P25398	RPS13	P62277	RPS14	P62263
RPS15	P62841	RPS15A	P62244	RPS16	P62249
RPS17	P08708	RPS18	P62269	RPS2	P15880, P46782
RPS23	P62266	RPS27	P42677, Q71UM5	RPS27A	P42677, P62979, P62987
RPS29	P62273	RPS3	P23396	RPS3A	P61247
RPS4X	P62701, Q8TD47	RPS5	P46782	RPS6	P62753
RPS8	P62241	RPS9	P46781	RPSA	P08865
SOS2	P42766				

16. Response of EIF2AK4 (GCN2) to amino acid deficiency (R-HSA-9633012)

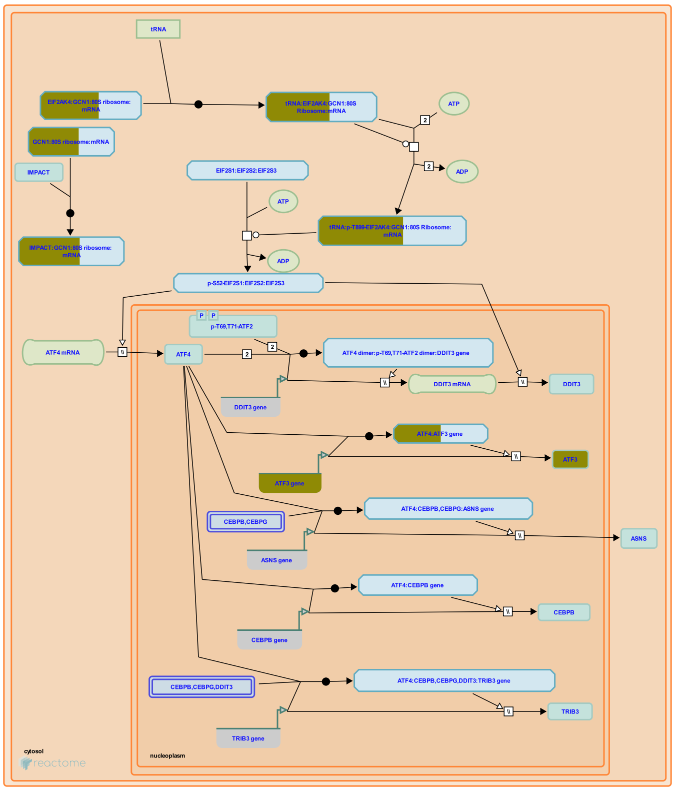

EIF2AK4 (GCN2) senses amino acid deficiency by binding uncharged tRNAs near the ribosome and responds by phosphorylating EIF2S1, the alpha subunit of the translation initiation factor EIF2 (inferred from yeast homologs and mouse homologs, reviewed in Chaveroux et al. 2010, Castilho et al. 2014, Gallinetti et al. 2013, Bröer and Bröer 2017, Wek 2018). Phosphorylated EIF2S1 reduces translation of most mRNAs but increases translation of downstream ORFs in mRNAs such as ATF4 that contain upstream ORFs (inferred from mouse homologs in Vattem and Wek 2004, reviewed in Hinnebusch et al. 2016, Sonenberg and Hinnebusch 2009). ATF4, in turn, activates expression of genes involved in responding to amino acid deficiency such as DDIT3 (CHOP), ASNS (asparagine synthetase), CEBPB, and ATF3 (reviewed in Kilberg et al. 2012, Wortel et al. 2017). In mice, EIF2AK4 in the brain may responsible for avoidance of diets lacking essential amino acids (Hao et al. 2005, Maurin et al. 2005, see also Leib and Knight 2015, Gietzen et al. 2016, reviewed in Dever and Hinnebusch 2005).

EIF2AK4 is bound to both the ribosome and GCN1, which is required for activation of EIF2AK4 and may act by shuttling uncharged tRNAs from the A site of the ribosome to EIF2AK4. Upon binding tRNA, EIF2AK4 trans-autophosphorylates. Phosphorylated EIF2AK4 then phosphorylates EIF2S1 on serine-52, the same serine residue phosphorylated by other kinases of the integrated stress response: EIF2AK1 (HRI, activated by heme deficiency and other stresses), EIF2AK2 (PKR, activated by double-stranded RNA), and EIF2AK3 (PERK, activated by unfolded proteins) (reviewed in Hinnebusch 1994, Wek et al. 2006, Donnelly et al. 2013, Pakos-Zebrucka et al. 2016, Wek 2018),

References

Koryga I, Pakos-Zebrucka K, Gorman AM, Samali A, Mnich K & Ljujic M (2016). The integrated stress response. EMBO Rep., 17, 1374-1395.

McGrath BC, Ross-Inta CM, Hao S, Koehnle TJ, McDaniel BJ, Sharp JW, ... Anthony TG (2005). Uncharged tRNA and sensing of amino acid deficiency in mammalian piriform cortex. Science, 307 , 1776-8.

Leib DE & Knight ZA (2015). Re-examination of Dietary Amino Acid Sensing Reveals a GCN2-Independent Mechanism. Cell Rep, 13, 1081-1089.

Shanmugam R, Himme BM, Sattlegger E, Silva RC, Ramesh R & Castilho BA (2014). Keeping the eIF2 alpha kinase Gcn2 in check. Biochim. Biophys. Acta, 1843, 1948-68.

Mitchell JR, Gallinetti J & Harputlugil E (2013). Amino acid sensing in dietary-restriction-mediated longevity: roles of signal-transducing kinases GCN2 and TOR. Biochem. J., 449, 1-10.

Edit history

Date	Action	Author
2018-12-28	Edited	May B
2018-12-28	Authored	May B
2018-12-28	Created	May B
2019-09-15	Reviewed	Bruhat A
2019-11-20	Reviewed	Staschke KA
2023-03-08	Modified	Matthews L

53 submitted entities found in this pathway, mapping to 68 Reactome entities

Input	UniProt Id	Input	UniProt Id	Input	UniProt Id
AGRN	P32969	ATF3	P18847	FAU	P62861
RPL10	P27635, Q96L21	RPL10A	P61313, P62906	RPL13	P26373, P40429
RPL13A	P40429, P61313	RPL15	P61313	RPL18	Q07020
RPL21	P46778	RPL27A	P46776, P61353	RPL29	P47914
RPL3	P39023, Q92901	RPL30	P62888	RPL31	P62899
RPL32	P62888, P62910	RPL34	P49207, P62899	RPL35	P42766
RPL35A	P18077	RPL36	Q9Y3U8	RPL38	P63173
RPL3L	Q92901	RPL4	P36578	RPL5	P46777
RPL7	P18124	RPL7A	P18124, P62424	RPL9P8	P32969
RPLP0	P05388	RPLP2	P05387	RPS10	P46783
RPS11	P62280	RPS12	P25398	RPS13	P62277
RPS14	P62263	RPS15	P62841	RPS15A	P62244
RPS16	P62249	RPS17	P08708	RPS18	P62269
RPS2	P15880, P46782	RPS23	P62266	RPS27	P42677, Q71UM5
RPS27A	P42677, P62979, P62987	RPS29	P62273	RPS3	P23396
RPS3A	P61247	RPS4X	P62701, Q8TD47	RPS5	P46782
RPS6	P62753	RPS8	P62241	RPS9	P46781
RPSA	P08865	SOS2	P42766		

Input	Ensembl Id
ATF3	ENSG00000162772

17. Selenocysteine synthesis (R-HSA-2408557)

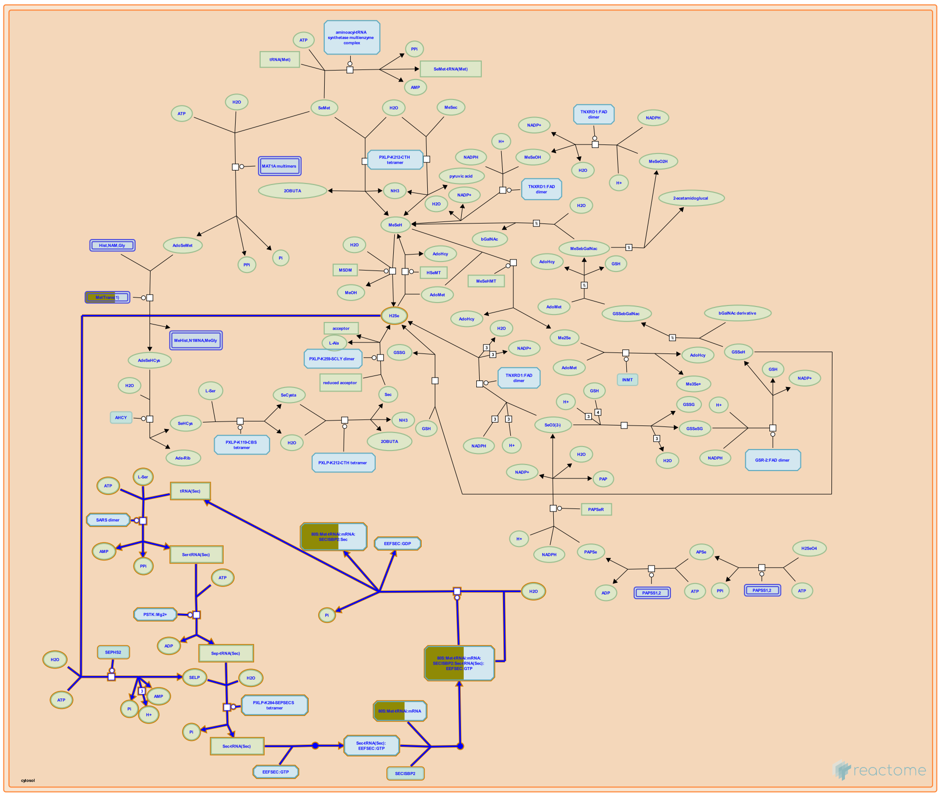

Selenocysteine, the 21st genetically encoded amino acid, is the major form of the antioxidant trace element selenium in the human body. In eukaryotes and archaea its synthesis proceeds through a phosphorylated intermediate in a tRNA-dependent fashion. The final step of selenocysteine formation is catalyzed by O-phosphoseryl-tRNA:selenocysteinyl-tRNA synthase (SEPSECS) that converts phosphoseryl-tRNA(Sec) to selenocysteinyl-tRNA(Sec).

References

Donovan J & Copeland PR (2010). Threading the needle: getting selenocysteine into proteins. Antioxid. Redox Signal., 12, 881-92.

Palioura S, Herkel J, Simonovic M, Lohse AW & Söll D (2010). Human SepSecS or SLA/LP: selenocysteine formation and autoimmune hepatitis. Biol. Chem., 391, 771-6.

Sheppard K, Yuan J, Devine KM, Jester B, Söll D & Hohn MJ (2008). From one amino acid to another: tRNA-dependent amino acid biosynthesis. Nucleic Acids Res., 36, 1813-25.

Edit history

Date	Action	Author
2012-07-17	Created	Williams MG
2014-05-06	Authored	Williams MG
2015-08-29	Edited	D'Eustachio P
2015-08-30	Reviewed	Rush MG
2023-08-26	Modified	Wright A

52 submitted entities found in this pathway, mapping to 66 Reactome entities

Input	UniProt Id	Input	UniProt Id	Input	UniProt Id
AGRN	P32969	FAU	P62861	RPL10	P27635, Q96L21
RPL10A	P61313, P62906	RPL13	P26373, P40429	RPL13A	P40429, P61313
RPL15	P61313	RPL18	Q07020	RPL21	P46778
RPL27A	P46776, P61353	RPL29	P47914	RPL3	P39023, Q92901
RPL30	P62888	RPL31	P62899	RPL32	P62888, P62910
RPL34	P49207, P62899	RPL35	P42766	RPL35A	P18077
RPL36	Q9Y3U8	RPL38	P63173	RPL3L	Q92901
RPL4	P36578	RPL5	P46777	RPL7	P18124
RPL7A	P18124, P62424	RPL9P8	P32969	RPLP0	P05388
RPLP2	P05387	RPS10	P46783	RPS11	P62280
RPS12	P25398	RPS13	P62277	RPS14	P62263
RPS15	P62841	RPS15A	P62244	RPS16	P62249
RPS17	P08708	RPS18	P62269	RPS2	P15880, P46782
RPS23	P62266	RPS27	P42677, Q71UM5	RPS27A	P42677, P62979, P62987
RPS29	P62273	RPS3	P23396	RPS3A	P61247
RPS4X	P62701, Q8TD47	RPS5	P46782	RPS6	P62753
RPS8	P62241	RPS9	P46781	RPSA	P08865
SOS2	P42766				

18. Viral mRNA Translation (R-HSA-192823)

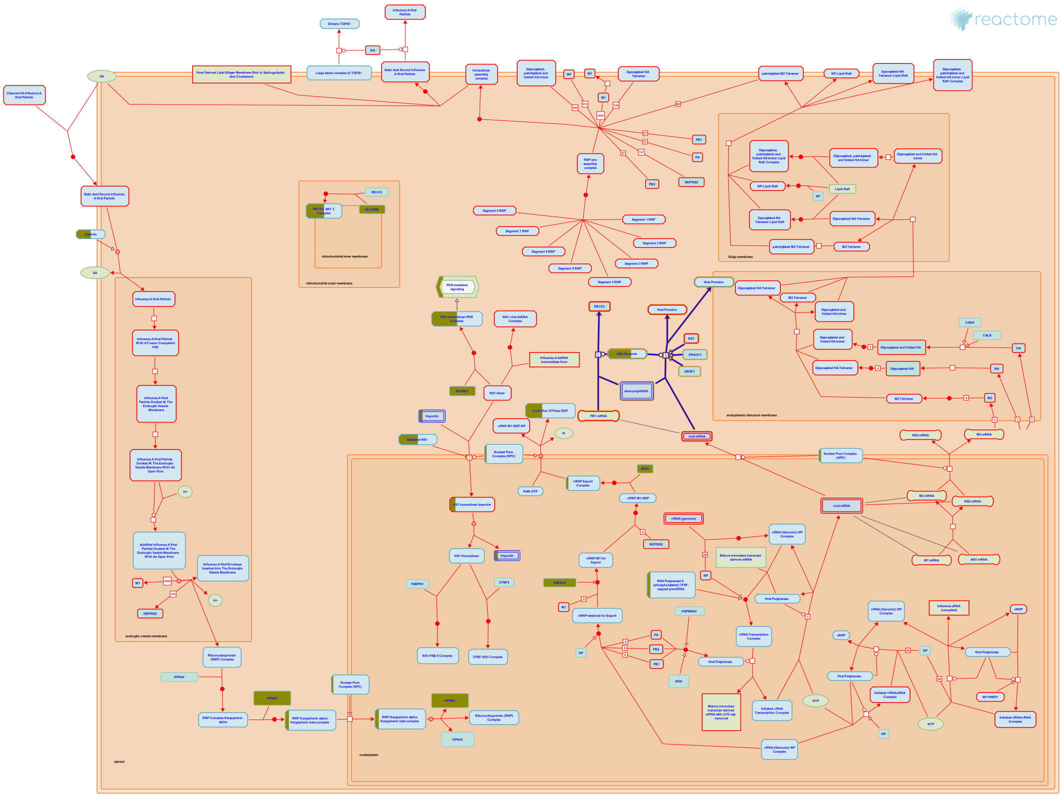

Cellular compartments: cytosol.

Diseases: influenza.

Spliced and unspliced viral mRNA in the cytoplasm are translated by host cell ribosomal translation machinery (reviewed in Kash, 2006). At least ten viral proteins are synthesized: HA, NA, PB1, PB2, PA, NP, NS1, NEP/NS2, M1, and M2. Viral mRNA translation is believed to be enhanced by conserved 5'UTR sequences that interact with the ribosomal machinery and at least one cellular RNA-binding protein, G-rich sequence factor 1 (GRSF-1), has been found to specifically interact with the viral 5' UTRs. (Park, 1995; Park, 1999). The viral NS1 protein and the cellular protein P58(IPK) enhance viral translation indirectly by preventing the activation of the translational inhibitor PKR (Salvatore, 2002; Goodman, 2006). The viral NS1 protein has also been proposed to specifically enhance translation through interaction with host poly(A)-binding protein 1 (PABP1) (Burgui, 2003). Simultaneously, host cell protein synthesis is downregulated in influenza virus infection through still uncharacterized mechanisms (Katze, 1986; Garfinkel, 1992; Kash, 2006). In most human influenza A strains (such as PR8), the PB1 mRNA segment is capable of producing a second protein, PB1-F2, from a short +1 open reading frame initiating downstream of the PB1 ORF initiation codon (Chen, 2001).

References

Korth MJ, Katze MG, Kash JC & Goodman AG (2006). Hijacking of the host-cell response and translational control during influenza virus infection. Virus Res, 119, 111-20.

Edit history

Date	Action	Author
2007-02-07	Created	Gillespie ME

Date	Action	Author
2007-02-13	Reviewed	Squires B
2007-02-13	Authored	Garcia-Sastre A, Bortz E
2023-08-31	Modified	Wright A

52 submitted entities found in this pathway, mapping to 66 Reactome entities

Input	UniProt Id	Input	UniProt Id	Input	UniProt Id
AGRN	P32969	FAU	P62861	RPL10	P27635, Q96L21
RPL10A	P61313, P62906	RPL13	P26373, P40429	RPL13A	P40429, P61313
RPL15	P61313	RPL18	Q07020	RPL21	P46778
RPL27A	P46776, P61353	RPL29	P47914	RPL3	P39023, Q92901
RPL30	P62888	RPL31	P62899	RPL32	P62888, P62910
RPL34	P49207, P62899	RPL35	P42766	RPL35A	P18077
RPL36	Q9Y3U8	RPL38	P63173	RPL3L	Q92901
RPL4	P36578	RPL5	P46777	RPL7	P18124
RPL7A	P18124, P62424	RPL9P8	P32969	RPLP0	P05388
RPLP2	P05387	RPS10	P46783	RPS11	P62280
RPS12	P25398	RPS13	P62277	RPS14	P62263
RPS15	P62841	RPS15A	P62244	RPS16	P62249
RPS17	P08708	RPS18	P62269	RPS2	P15880, P46782
RPS23	P62266	RPS27	P42677, Q71UM5	RPS27A	P42677, P62979, P62987
RPS29	P62273	RPS3	P23396	RPS3A	P61247
RPS4X	P62701, Q8TD47	RPS5	P46782	RPS6	P62753
RPS8	P62241	RPS9	P46781	RPSA	P08865
SOS2	P42766				

19. Eukaryotic Translation Initiation (R-HSA-72613)

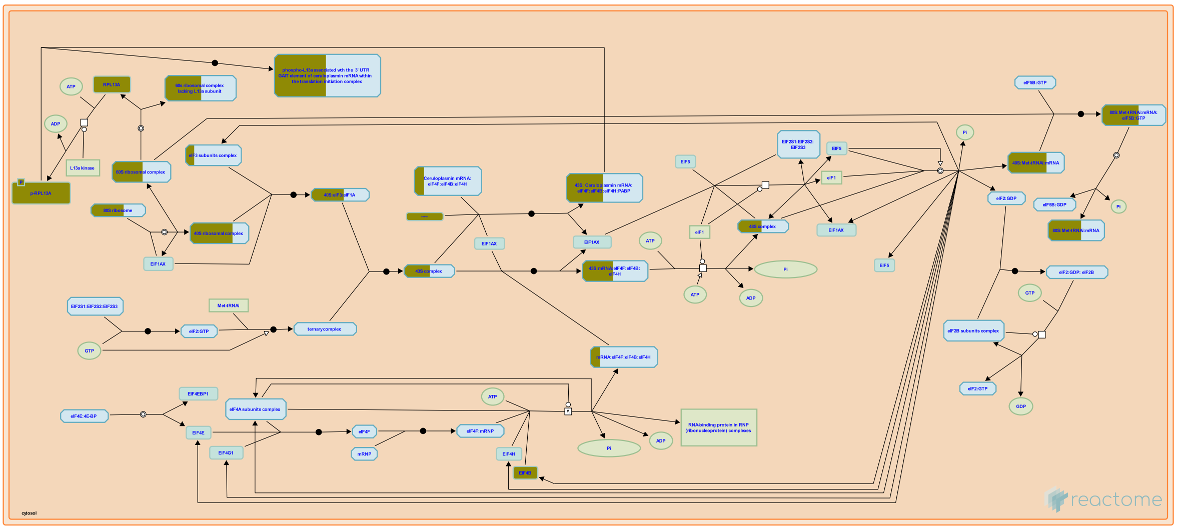

Initiation of translation in the majority of eukaryotic cellular mRNAs depends on the 5'-cap (m7GpppN) and involves ribosomal scanning of the 5' untranslated region (5'-UTR) for an initiating AUG start codon. Therefore, this mechanism is often called cap-dependent translation initiation. Proximity to the cap, as well as the nucleotides surrounding an AUG codon, influence the efficiency of the start site recognition during the scanning process. However, if the recognition site is poor enough, scanning ribosomal subunits will ignore and skip potential starting AUGs, a phenomenon called leaky scanning. Leaky scanning allows a single mRNA to encode several proteins that differ in their amino-termini. Merrick (2010) provides an overview of this process and hghlights several features of it that remain incompletely understood.

Several eukaryotic cell and viral mRNAs initiate translation by an alternative mechanism that involves internal initiation rather than ribosomal scanning. These mRNAs contain complex nucleotide sequences, called internal ribosomal entry sites, where ribosomes bind in a cap-independent manner and start translation at the closest downstream AUG codon.

Initiation on several viral and cellular mRNAs is cap-independent and is mediated by binding of the ribosome to internal ribosome entry site (IRES) elements. These elements are often found in characteristically long structured regions on the 5'-UTR of an mRNA that may or may not have regulatory upstream open reading frames (uORFs). Both of these features on the 5'-end of the mRNA hinder ribosomal scanning, and thus promote a cap-independent translation initiation mechanism. IRESs act as specific translational enhancers that allow translation initiation to occur in response to specific stimuli and under the control of different trans-acting factors, as for example when cap-dependent protein synthesis is shut off during viral infection. Such regulatory elements have been identified in the mRNAs of growth factors, protooncogenes, angiogenesis factors, and apoptosis regulators, which are translated under a variety of stress conditions, including hypoxia, serum deprivation, irradiation and apoptosis. Thus, cap-independent translational control might have evolved to regulate cellular responses in acute but transient stress conditions that would otherwise lead to cell death, while the same mechanism is of major importance for viral mRNAs to bypass the shutting-off of host protein synthesis after infection. Encephalomyocarditis virus (EMCV) and hepatitis C virus exemplify two distinct mechanisms of IRES-mediated initiation. In contrast to cap-dependent initiation, the eIF4A and eIF4G subunits of eIF4F bind immediately upstream of the EMCV initiation codon and promote binding of a 43S complex. Accordingly, EMCV initiation does not involve scanning and does not require eIF1, eIF1A, and the eIF4E subunit of eIF4F. Nonetheless, initiation on some EMCV-like IRESs requires additional non-canonical initiation factors, which alter IRES conformation and promote binding of eIF4A/eIF4G. Initiation on the hepatitis C virus IRES is simpler: a 43S complex containing only eIF2 and eIF3 binds directly to the initiation codon as a result of specific interaction of the IRES and the 40S subunit.

References

Merrick WC (2010). Eukaryotic protein synthesis: still a mystery. J Biol Chem, 285, 21197-201.

Edit history

Date	Action	Author
2002-12-16	Created	Merrick WC
2023-08-26	Modified	Wright A

56 submitted entities found in this pathway, mapping to 70 Reactome entities

Input	UniProt Id	Input	UniProt Id	Input	UniProt Id
AGRN	P32969	EIF3F	O00303	EIF3L	Q9Y262
EIF4B	P23588	FAU	P62861	PABPC1	P11940
RPL10	P27635, Q96L21	RPL10A	P61313, P62906	RPL13	P26373, P40429
RPL13A	P40429, P61313	RPL15	P61313	RPL18	Q07020
RPL21	P46778	RPL27A	P46776, P61353	RPL29	P47914
RPL3	P39023, Q92901	RPL30	P62888	RPL31	P62899
RPL32	P62888, P62910	RPL34	P49207, P62899	RPL35	P42766
RPL35A	P18077	RPL36	Q9Y3U8	RPL38	P63173
RPL3L	Q92901	RPL4	P36578	RPL5	P46777
RPL7	P18124	RPL7A	P18124, P62424	RPL9P8	P32969
RPLP0	P05388	RPLP2	P05387	RPS10	P46783
RPS11	P62280	RPS12	P25398	RPS13	P62277
RPS14	P62263	RPS15	P62841	RPS15A	P62244
RPS16	P62249	RPS17	P08708	RPS18	P62269

Input	UniProt Id	Input	UniProt Id	Input	UniProt Id
RPS2	P15880, P46782	RPS23	P62266	RPS27	P42677, Q71UM5
RPS27A	P42677, P62979, P62987	RPS29	P62273	RPS3	P23396
RPS3A	P61247	RPS4X	P62701, Q8TD47	RPS5	P46782
RPS6	P62753	RPS8	P62241	RPS9	P46781
RPSA	P08865	SOS2	P42766		

20. Cap-dependent Translation Initiation (R-HSA-72737)

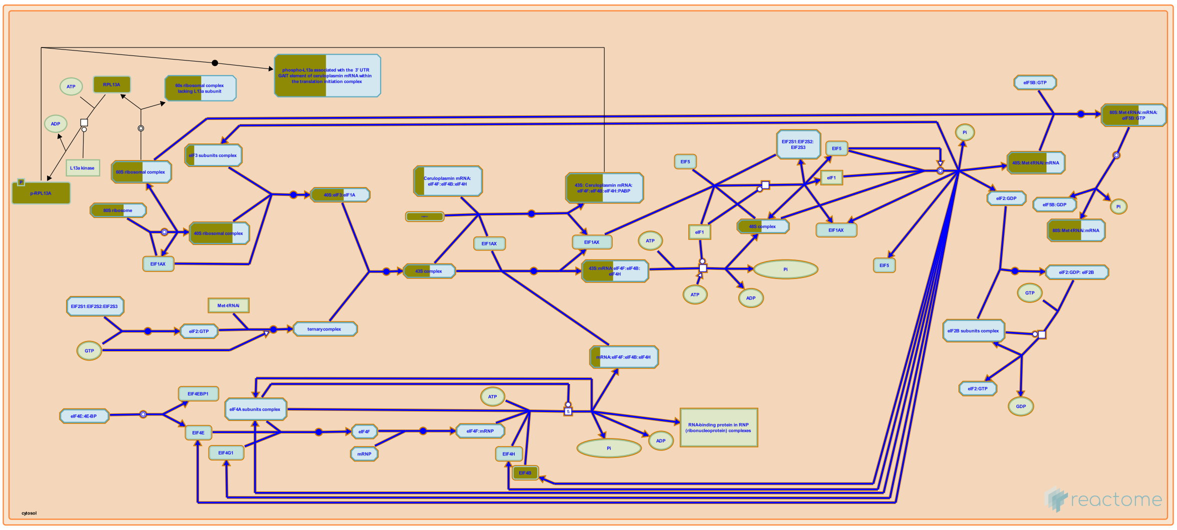

Translation initiation is a complex process in which the Met-tRNAi initiator, 40S, and 60S ribosomal subunits are assembled by eukaryotic initiation factors (eIFs) into an 80S ribosome at the start codon of an mRNA. The basic mechanism for this process can be described as a series of five steps: 1) formation of a pool of free 40S subunits, 2) formation of the ternary complex (Met-tRNAi/eIF2/GTP), and subsequently, the 43S complex (comprising the 40S subunit, Met-tRNAi/eIF2/GTP, eIF3 and eIF1A), 3) activation of the mRNA upon binding of the cap-binding complex eIF4F, and factors eIF4A, eIF4B and eIF4H, with subsequent binding to the 43S complex, 4) ribosomal scanning and start codon recognition, and 5) GTP hydrolysis and joining of the 60S ribosomal subunit.

References

Edit history

Date	Action	Author
2002-12-16	Created	Merrick WC
2023-08-26	Modified	Wright A

56 submitted entities found in this pathway, mapping to 70 Reactome entities

Input	UniProt Id	Input	UniProt Id	Input	UniProt Id
AGRN	P32969	EIF3F	O00303	EIF3L	Q9Y262
EIF4B	P23588	FAU	P62861	PABPC1	P11940
RPL10	P27635, Q96L21	RPL10A	P61313, P62906	RPL13	P26373, P40429
RPL13A	P40429, P61313	RPL15	P61313	RPL18	Q07020
RPL21	P46778	RPL27A	P46776, P61353	RPL29	P47914
RPL3	P39023, Q92901	RPL30	P62888	RPL31	P62899
RPL32	P62888, P62910	RPL34	P49207, P62899	RPL35	P42766
RPL35A	P18077	RPL36	Q9Y3U8	RPL38	P63173
RPL3L	Q92901	RPL4	P36578	RPL5	P46777
RPL7	P18124	RPL7A	P18124, P62424	RPL9P8	P32969
RPLP0	P05388	RPLP2	P05387	RPS10	P46783
RPS11	P62280	RPS12	P25398	RPS13	P62277
RPS14	P62263	RPS15	P62841	RPS15A	P62244

Input	UniProt Id
RPS16	P62249
RPS2	P15880, P46782
RPS27A	P42677, P62979, P62987
RPS3A	P61247
RPS6	P62753
RPSA	P08865

Input	UniProt Id
RPS17	P08708
RPS23	P62266
RPS29	P62273
RPS4X	P62701, Q8TD47
RPS8	P62241
SOS2	P42766

Input	UniProt Id
RPS18	P62269
RPS27	P42677, Q71UM5
RPS3	P23396
RPS5	P46782
RPS9	P46781

21. Immunoregulatory interactions between a Lymphoid and a non-Lymphoid cell (R-HSA-198933)

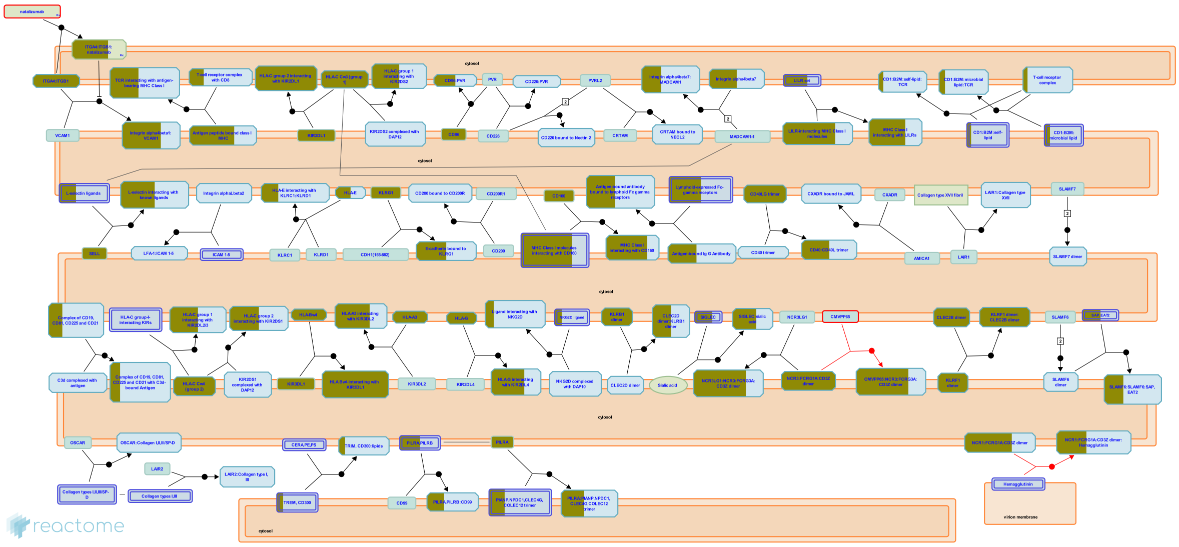

A number of receptors and cell adhesion molecules play a key role in modifying the response of cells of lymphoid origin (such as B-, T- and NK cells) to self and tumor antigens, as well as to pathogenic organisms.

Molecules such as KIRs and LILRs form part of a crucial surveillance system that looks out for any derangement, usually caused by cancer or viral infection, in MHC Class I presentation. Somatic cells are also able to report internal functional impairment by displaying surface stress markers such as MICA. The presence of these molecules on somatic cells is picked up by C-lectin NK immune receptors.

Lymphoid cells are able to regulate their location and movement in accordance to their state of activation, and home in on tissues expressing the appropriate complementary ligands. For example, lymphoid cells may fine tune the presence and concentration of adhesion molecules belonging to the IgSF, Selectin and Integrin class that interact with a number of vascular markers of inflammation.

Furthermore, there are a number of avenues through which lymphoid cells may interact with antigen. This may be presented directly to a specific T-cell receptor in the context of an MHC molecule. Antigen-antibody complexes may anchor to the cell via a small number of lymphoid-specific Fc receptors that may, in turn, influence cell function further. Activated complement factor C3d binds to both antigen and to cell surface receptor CD21. In such cases, the far-reaching influence of CD19 on B-lymphocyte function is tempered by its interaction with CD21.

References

Kelley J, Trowsdale J & Walter L (2005). Comparative genomics of natural killer cell receptor gene clusters. PLoS Genet., 1, 129-39.

Tomasello E, Walzer T, Vivier E, Baratin M & Ugolini S (2008). Functions of natural killer cells. Nat. Immunol., 9, 503-10.

Vivier E, Harris J, Trowsdale J, Vely F, Nedvetzki S, Davis DM, ... Pende D (2007). Reciprocal regulation of human natural killer cells and macrophages associated with distinct immune synapses. Blood, 109, 3776-85.

Batista FD & Carrasco YR (2006). B cell recognition of membrane-bound antigen: an exquisite way of sensing ligands. Curr Opin Immunol, 18, 286-91.

Shaw A & Cemerski S (2006). Immune synapses in T-cell activation. Curr Opin Immunol, 18, 298-304 .

Edit history

Date	Action	Author
2007-07-08	Authored	de Bono B
2007-07-08	Created	de Bono B
2007-08-06	Reviewed	Trowsdale J
2015-03-27	Authored	Garapati P V
2015-05-13	Reviewed	Barrow AD
2023-08-26	Modified	Wright A

77 submitted entities found in this pathway, mapping to 86 Reactome entities

Input	UniProt Id	Input	UniProt Id	Input	UniProt Id
B2M	P61769	CD160	O95971	CD1C	P29017
CD22	P20273	CD247	P20963-1	CD300E	Q496F6
CD34	P28906-1	CD40LG	P29965	CD8B	P10966
CD96	P40200	CLEC2B	Q92478	FCGR1A	P12314
FCGR3A	P08637	HLA-A	P04439	HLA-B	P01889
HLA-C	P10321	HLA-G	P17693	HLA-H	P01893
IFITM1	P13164	IFITM3	P13164	IGHV1-69	P01742
IGHV3-11	P01762	IGHV3-23	P01764	IGHV3-30	P01768
IGHV3-33	P01772	IGHV4-34	P06331	IGHV4-39	P01824
IGHV4-59	P01825	IGKC	P01834	IGKV1-12	A0A0C4DH73
IGKV1-17	P01599	IGKV1-33	P01594	IGKV1-5	P01602
IGKV1D-39	P04432	IGKV3-11	P04433	IGKV3-15	P01624
IGKV3-20	P01619	IGLC1	P0CG04	IGLC2	P0DOY2
IGLC3	P0DOY3	IGLC7	A0M8Q6	IGLV1-40	P01703, Q5NV69
IGLV1-44	P01699, Q5NV81	IGLV1-47	P01700	IGLV1-51	P01701
IGLV2-14	P01704	IGLV2-23	P01705, Q5NV89	IGLV3-1	P01715
IGLV3-19	P01714	IGLV3-21	P80748	IGLV3-25	P01717, Q5NV90
IGLV6-57	P01721	ITGA4	P13612	ITGB1	P05556
KIR2DL1	P43626, P43629	KIR3DL1	P43629	KLRB1	Q12918
KLRF1	Q9NZS2	KLRG1	Q96E93	LILRA4	P59901
LILRA5	A6NI73, Q8N149	LILRA6	Q6PI73	LILRB1	Q8NHL6
LILRB2	Q8N423	LILRB4	Q8N149, Q8NHJ6	MAL	P01732
MICB	Q29980	NCR3	O14931	NPDC1	Q9NQX5
PILRA	Q9UKJ1	SELL	P14151	SH2D1B	O14796
SIGLEC1	Q9BZZ2	SIGLEC5	O15389, O43699	SIGLEC7	Q9Y286
SIGLEC8	Q96PQ1, Q9NYZ4	TREML1	Q86YW5		

22. Regulation of expression of SLITs and ROBOs (R-HSA-9010553)

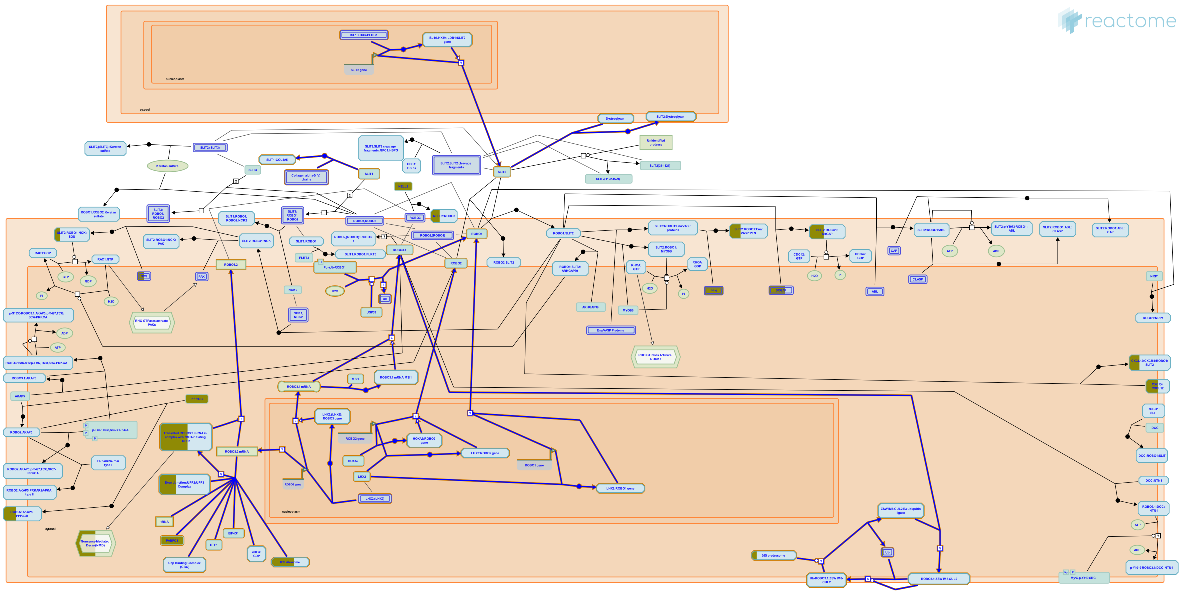

Expression of SLIT and ROBO proteins is regulated at the level of transcription, translation and protein localization and stability. LIM-homeodomain transcription factors LHX2, LHX3, LHX4, LHX9 and ISL1 have so far been implicated in a cell type-dependent transcriptional regulation of ROBO1, ROBO2, ROBO3 and SLIT2 (Wilson et al. 2008, Marcos-Mondejar et al. 2012, Kim et al. 2016). Homeobox transcription factor HOXA2 is involved in transcriptional regulation of ROBO2 (Geisen et al. 2008). Transcription of SLIT1 during optic tract development in Xenopus is stimulated by FGF signaling and may also involve the transcription factor HOXA2, but the mechanism has not been established (Atkinson-Leadbeater et al. 2010). PAX6 and the homeodomain transcription factor NKX2.2 are also implicated in regulation of SLIT1 transcription (Genethliou et al. 2009). An RNA binding protein, MSI1, binds ROBO3 mRNA and promotes its translation, thus increasing ROBO3 protein levels (Kuwako et al. 2010). A poorly studied E3 ubiquitin ligase ZSWIM8 promotes degradation of ROBO3 (Wang et al. 2013). ROBO1 is protein half-life is increased via deubiquitination of ROBO1 by a ubiquitin protease USP33 (Yuasa-Kawada et al. 2009, Huang et al. 2015). Interaction of SLIT2 with DAG1 (dystroglycan) is important for proper localization of SLIT2 at the floor plate (Wright et al. 2012). Interaction of SLIT1 with a type IV collagen COL4A5 is important for localization of SLIT1 to the basement membrane of the optical tectum (Xiao et al. 2011).

References

Rao Y, Kinoshita-Kawada M, Wu JY & Yuasa-Kawada J (2009). Deubiquitinating enzyme USP33/VDU1 is required for Slit signaling in inhibiting breast cancer cell migration. Proc. Natl. Acad. Sci. U.S.A., 106, 14530-5.

Ma L, Leung H, Lyon KA, Leahy DJ, Wright KM & Ginty DD (2012). Dystroglycan organizes axon guidance cue localization and axonal pathfinding. Neuron, 76, 931-44.

Brunet JF, Rijli FM, Pasqualetti M, Geisen MJ, Chédotal A, Di Meglio T & Ducret S (2008). Hox paralog group 2 genes control the migration of mouse pontine neurons through slit-robo signaling. PLoS Biol., 6, e142.

Dodd J, Shafer B, Lee KJ & Wilson SI (2008). A molecular program for contralateral trajectory: Rig-1 control by LIM homeodomain transcription factors. Neuron, 59, 413-24.

Zhu L, Wen P, Liu J, Kong R, Wu JY, Chen X, ... Quan C (2015). USP33 mediates Slit-Robo signaling in inhibiting colorectal cancer cell migration. Int. J. Cancer, 136, 1792-802.

Edit history

Date	Action	Author
2017-06-27	Authored	Orlic-Milacic M
2017-06-27	Created	Orlic-Milacic M
2017-07-31	Reviewed	Jaworski A
2017-08-04	Edited	Orlic-Milacic M
2023-08-26	Modified	Wright A

61 submitted entities found in this pathway, mapping to 75 Reactome entities

Input	UniProt Id	Input	UniProt Id	Input	UniProt Id
AGRN	P32969	CASC3	O15234	FAU	P62861
MAGOH	P61326	PABPC1	P11940	PSMB8	P28062
PSMB9	P28065	PSME1	Q06323	PSME2	Q9UL46
RPL10	P27635, Q96L21	RPL10A	P61313, P62906	RPL13	P26373, P40429
RPL13A	P40429, P61313	RPL15	P61313	RPL18	Q07020
RPL21	P46778	RPL27A	P46776, P61353	RPL29	P47914
RPL3	P39023, Q92901	RPL30	P62888	RPL31	P62899
RPL32	P62888, P62910	RPL34	P49207, P62899	RPL35	P42766
RPL35A	P18077	RPL36	Q9Y3U8	RPL38	P63173
RPL3L	Q92901	RPL4	P36578	RPL5	P46777
RPL7	P18124	RPL7A	P18124, P62424	RPL9P8	P32969
RPLP0	P05388	RPLP2	P05387	RPN2	Q99460
RPS10	P46783	RPS11	P62280	RPS12	P25398
RPS13	P62277	RPS14	P62263	RPS15	P62841
RPS15A	P62244	RPS16	P62249	RPS17	P08708
RPS18	P62269	RPS2	P15880, P46782	RPS23	P62266
RPS27	P42677, Q71UM5	RPS27A	P42677, P62979, P62987	RPS29	P62273
RPS3	P23396	RPS3A	P61247	RPS4X	P62701, Q8TD47
RPS5	P46782	RPS6	P62753	RPS8	P62241
RPS9	P46781	RPSA	P08865	SOS2	P42766
UPF3B	Q9BZI7				

23. Cellular response to starvation (R-HSA-9711097)

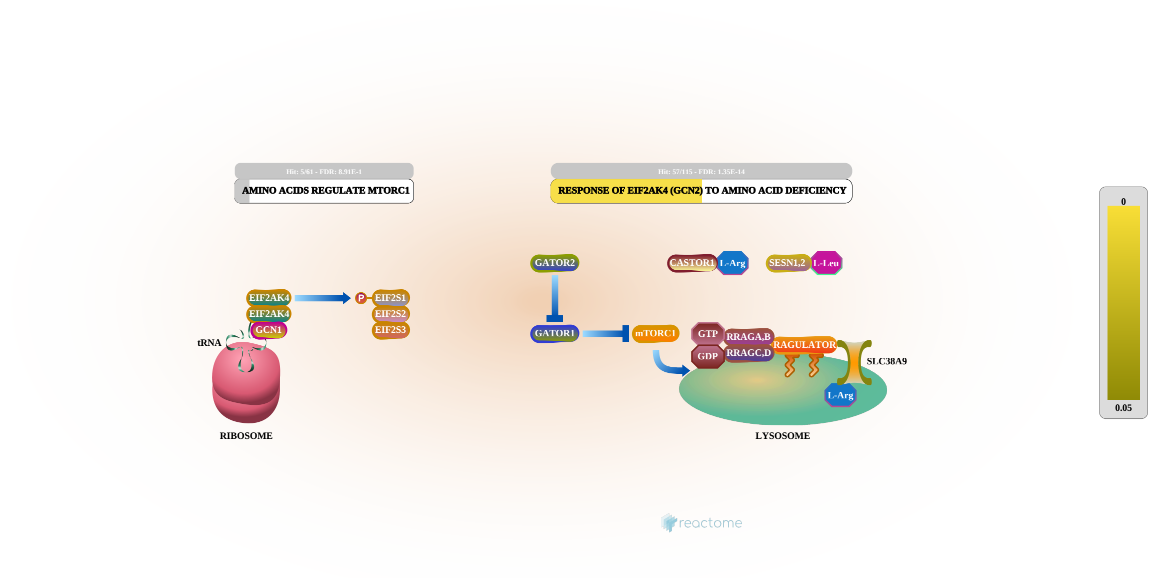

Deprivation of nutrients triggers diverse short- and long terms adaptations in cells. Here we have annotated two aspects of cellular responses to amino acid deprivation, ones mediated by EIF2AK4 and ones mediated by mTORC.

EIF2AK4 (GCN2) senses amino acid deficiency by binding uncharged tRNAs near the ribosome, and phosphorylating EIF2S1 (reviewed in Chaveroux et al. 2010, Castilho et al. 2014, Gallinetti et al. 2013, Bröer and Bröer 2017, Wek 2018). This reduces translation of most mRNAs but increases translation of mRNAs, notably ATF4, that mediate stress responses (reviewed in Kilberg et al. 2012, Wortel et al. 2017; Dever and Hinnebusch 2005).

The mTORC1 complex acts as an integrator that regulates translation, lipid synthesis, autophagy, and cell growth in response to multiple inputs, notably glucose, oxygen, amino acids, and growth factors such as insulin (reviewed in Sabatini 2017, Meng et al. 2018, Kim and Guan 2019).

MTOR, the kinase subunit of mTORC1, is activated by interaction with RHEB:GTP at the cytosolic face of lysosomal membrane (Long et al. 2005, Tee et al. 2005, Long et al. 2007, Yang et al. 2017). This process is regulated by various individual amino acids (reviewed in Zhuang et al. 2019, Wolfson and Sabatini 2017, Yao et al. 2017) and is reversed in response to the removal of amino acids, through the action of TSC1 (Demetriades et al. 2014).

References

Busch S, Lin Y, Long X, Avruch J & Ortiz-Vega S (2007). The Rheb switch 2 segment is critical for signaling to target of rapamycin complex 1. J. Biol. Chem., 282, 18542-51.

Demetriades C, Teleman AA & Doumpas N (2014). Regulation of TORC1 in response to amino acid starvation via lysosomal recruitment of TSC2. Cell, 156, 786-99.

Sabatini DM (2017). Twenty-five years of mTOR: Uncovering the link from nutrients to growth. Proc. Natl. Acad. Sci. U.S.A., 114, 11818-11825.

Sabatini DM & Wolfson RL (2017). The Dawn of the Age of Amino Acid Sensors for the mTORC1 Pathway. Cell Metab., 26, 301-309.

Yang A, Yang HJ, Jiang X, Li B, Pavletich NP, Yang H, ... Miller M (2017). Mechanisms of mTORC1 activation by RHEB and inhibition by PRAS40. Nature, 552, 368-373.

Edit history

Date	Action	Author
2020-11-19	Authored	Stephan R
2021-01-13	Created	Stephan R
2021-02-19	Edited	D'Eustachio P
2023-08-26	Modified	Wright A

58 submitted entities found in this pathway, mapping to 73 Reactome entities

Input	UniProt Id	Input	UniProt Id	Input	UniProt Id
AGRN	P32969	ATF3	P18847	ATP6V0E2	Q8NHE4
FAU	P62861	FLCN	Q8NFG4	KPTN	Q9Y664
LAMTOR1	Q6IAA8	RPL10	P27635, Q96L21	RPL10A	P61313, P62906
RPL13	P26373, P40429	RPL13A	P40429, P61313	RPL15	P61313
RPL18	Q07020	RPL21	P46778	RPL27A	P46776, P61353
RPL29	P47914	RPL3	P39023, Q92901	RPL30	P62888
RPL31	P62899	RPL32	P62888, P62910	RPL34	P49207, P62899
RPL35	P42766	RPL35A	P18077	RPL36	Q9Y3U8
RPL38	P63173	RPL3L	Q92901	RPL4	P36578
RPL5	P46777	RPL7	P18124	RPL7A	P18124, P62424
RPL9P8	P32969	RPLP0	P05388	RPLP2	P05387
RPS10	P46783	RPS11	P62280	RPS12	P25398
RPS13	P62277	RPS14	P62263	RPS15	P62841
RPS15A	P62244	RPS16	P62249	RPS17	P08708
RPS18	P62269	RPS2	P15880, P46782	RPS23	P62266
RPS27	P42677, Q71UM5	RPS27A	P42677, P62979, P62987	RPS29	P62273
RPS3	P23396	RPS3A	P61247	RPS4X	P62701, Q8TD47
RPS5	P46782	RPS6	P62753	RPS8	P62241
RPS9	P46781	RPSA	P08865	SESN1	Q9Y6P5
SOS2	P42766				

Input	Ensembl Id
ATF3	ENSG00000162772

24. SARS-CoV-1 modulates host translation machinery (R-HSA-9735869)

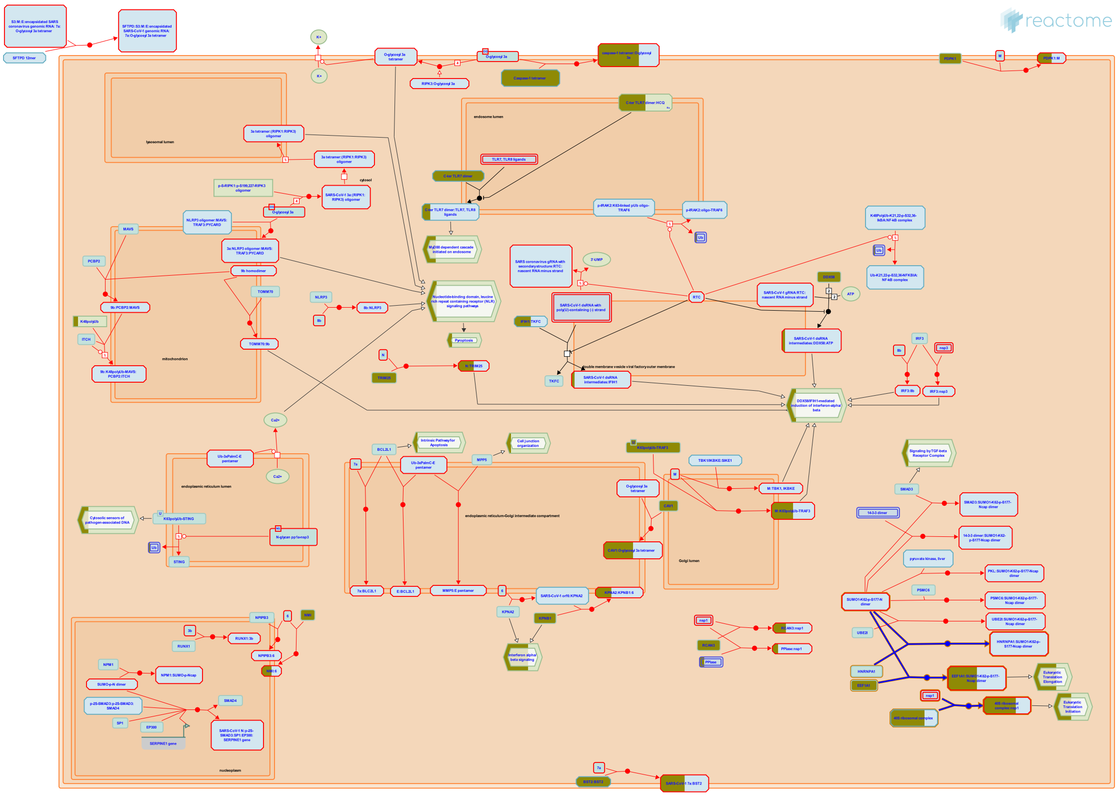

Diseases: severe acute respiratory syndrome.

Severe acute respiratory syndrome coronavirus type 1 (SARS-CoV-1) nonstructural protein 1 (nsp1) and nucleocapsid protein (N) disrupt mRNA translation upon SARS-CoV-1 infection in human cells.

References

Huang C, Makino S, Lokugamage KG & Narayanan K (2012). Severe acute respiratory syndrome coronavirus protein nsp1 is a novel eukaryotic translation inhibitor that represses multiple steps of translation initiation. J Virol, 86, 13598-608.

Cao C, Wang Q, Zhou B, Li X, Ma Q, Liu J, ... Liu X (2008). The nucleocapsid protein of severe acute respiratory syndrome coronavirus inhibits cell cytokinesis and proliferation by interacting with translation elongation factor 1alpha. J Virol, 82, 6962-71.

Edit history

Date	Action	Author
2020-06-25	Authored	Shamovsky V
2021-01-26	Reviewed	D'Eustachio P
2021-05-12	Authored	Stephan R
2021-07-05	Created	Shamovsky V
2022-08-11	Edited	Shamovsky V
2023-03-08	Modified	Matthews L

25 submitted entities found in this pathway, mapping to 29 Reactome entities

Input	UniProt Id	Input	UniProt Id	Input	UniProt Id
EEF1A1	P68104	FAU	P62861	RPS10	P46783
RPS11	P62280	RPS12	P25398	RPS13	P62277
RPS14	P62263	RPS15	P62841	RPS15A	P62244
RPS16	P62249	RPS17	P08708	RPS18	P62269
RPS2	P15880, P46782	RPS23	P62266	RPS27	P42677, Q71UM5
RPS27A	P42677, P62979	RPS29	P62273	RPS3	P23396
RPS3A	P61247	RPS4X	P62701, Q8TD47	RPS5	P46782
RPS6	P62753	RPS8	P62241	RPS9	P46781
RPSA	P08865				

25. FCGR activation (R-HSA-2029481)

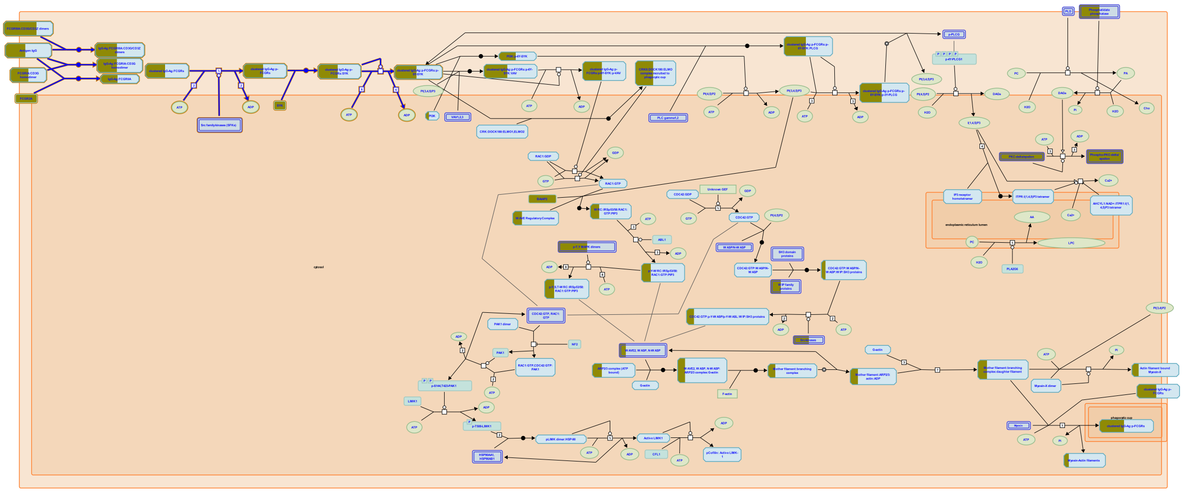

Cellular compartments: plasma membrane, cytosol.

Cross-linking of FCGRs with IgG coated immune complexes results in tyrosine phosphorylation of the immuno tyrosine activation motif (ITAMs) of the rececptor by membrane-bound tyrosine kinases of the SRC family. The phosphorylated ITAM tyrosines serve as docking sites for Src homology 2 (SH2) domain-containing SYK kinase. Recruitment and activation of SYK is critical for FCGR-mediated signaling in phagocytosis, but the exact role of SYK in this process is unclear. Activated SYK then transmits downstream signals leading to actin polymerization and particle internalization.

References

Rosales C & García-García E (2002). Signal transduction during Fc receptor-mediated phagocytosis. J Leukoc Biol, 72, 1092-108.

Tridandapani S, Joshi T & Butchar JP (2006). Fcgamma receptor signaling in phagocytes. Int J Hematol, 84, 210-6.

Dart AE, Caron E, Groves E & Covarelli V (2008). Molecular mechanisms of phagocytic uptake in mammalian cells. Cell Mol Life Sci, 65, 1957-76.

Edit history

Date	Action	Author
2012-01-04	Edited	Garapati P V
2012-01-04	Authored	Garapati P V
2012-01-04	Created	Garapati P V
2012-05-15	Reviewed	Rosales C
2023-08-26	Modified	Wright A

40 submitted entities found in this pathway, mapping to 45 Reactome entities

Input	UniProt Id	Input	UniProt Id	Input	UniProt Id
CD247	P20963-1	FCGR1A	P12314	FCGR2A	P12318

Input	UniProt Id	Input	UniProt Id	Input	UniProt Id
FCGR3A	P08637	IGHG1	P01857, P01861	IGHG3	P01860
IGHG4	P01861	IGHV1-69	P01742	IGHV3-11	P01762
IGHV3-23	P01764	IGHV3-30	P01768	IGHV3-33	P01772
IGHV4-34	P06331	IGHV4-39	P01824	IGHV4-59	P01825
IGKC	P01834	IGKV1-12	A0A0C4DH73	IGKV1-17	P01599
IGKV1-33	P01594	IGKV1-5	P01602	IGKV1D-39	P04432
IGKV3-11	P04433	IGKV3-15	P01624	IGKV3-20	P01619
IGLC1	P0CG04	IGLC2	P0DOY2	IGLC3	P0DOY3
IGLC7	A0M8Q6	IGLV1-40	P01703, Q5NV69	IGLV1-44	P01699, Q5NV81
IGLV1-47	P01700	IGLV1-51	P01701	IGLV2-14	P01704
IGLV2-23	P01705, Q5NV89	IGLV3-1	P01715	IGLV3-19	P01714
IGLV3-21	P80748	IGLV3-25	P01717, Q5NV90	IGLV6-57	P01721
SYK	P43405				

6. Identifiers found

Below is a list of the input identifiers that have been found or mapped to an equivalent element in Reactome, classified by resource.

1084 of the submitted entities were found, mapping to 1396 Reactome entities

Input	UniProt Id	Input	UniProt Id	Input	UniProt Id
AACS	Q86V21	ABAT	P80404	ABCA1	O95477
ABCB1	P08183	ABCB10	Q9NRK6	ABCG1	P45844
ABHD17A	Q96GS6	ABHD4	Q8TB40	ABLIM1	O14639
ACOT8	O14734	ACOT9	Q9Y305	ACP6	Q9NPH0
ACSL1	P33121	ACTA2	P62736	ACTR3	P61158
ADA2	Q9NZK5	ADAM12	O43184	ADAM9	Q13443
ADAMTS1	Q9UHI8	ADAMTS10	Q9H324	ADAMTS2	O95450
ADAMTSL4	Q6UY14	ADAR	P55265	ADCY4	Q8NFM4
ADGRE3	Q9BY15	ADGRG1	Q86Y34	ADM	P35318
ADRA1D	P25100	ADRA2B	P18089	AGRN	P32969
AIM2	O14862	AK4	P27144	AK5	Q9Y6K8
AKAP1	Q92667	AKR1B1	P15121	AKR1C3	P42330
ALDOA	P04075	ALDOC	P09972	ALKBH2	Q6NS38
ALOX15	P16050	ALPL	P05186	ALS2CL	Q60I27
AMY1A	P0DUB6	AMY2A	P04746, P19961	AMY2B	P19961
ANKFY1	Q9P2R3	ANKH	Q9HCJ1	ANPEP	P15144
AP1B1	Q10567	APBA2	Q99767	APBB1	O00213
APOBEC3A	P31941	APOBEC3B	Q9UH17	APOBEC3G	Q9HC16-1, Q9HC16-3
APOBEC3H	Q6NTF7	APOD	P05090	APOL1	O14791
ARF1	P84077	ARF4	P18085	ARL2	P36404
ARL3	Q13795	ARPC1B	O15143	ARRB1	P49407
ASCC2	Q9H1I8	ASIC1	P78348	ATAD2	Q6PL18
ATF3	P18847	ATG4B	Q96DT6, Q9Y4P1	ATG9B	Q674R7
ATP1A1	P05023	ATP1B1	P05026	ATP1B3	P54709
ATP2C1	P98194	ATP5MC2	Q06055	ATP5PD	O75947
ATP6V0E2	Q8NHE4	AURKA	O14965	AUTS2	Q8WXX7
AXIN2	Q9Y2T1	B2M	P61769	B3GALT2	O43825
BABAM1	Q9NWV8	BAG3	O95817	BAG6	P46379
BAIAP2	Q9UQB8	BANP	Q8N9N5	BBS7	Q8IWZ6
BCL2A1	Q16548	BCL2L14	Q9BZR8	BCL9	O00512
BCR	P11274	BIRC2	Q13489, Q13490	BLVRA	P53004
BMPR2	Q13873	BRCA1	P38398	BRCA2	P51587
BRI3	O95415	BST1	Q10588	BST2	Q10589
BTF3	O00478, P78410	BTK	Q06187	BTN2A2	Q8WVV5, Q9UIR0
BTN3A1	O00481	BTNL8	Q6UX41	BUB1	O43683, O60566
C1GALT1	Q9NS00	C1QA	P02745	C1QB	P02746
C1QC	P02747	C2	P06681	C3AR1	Q16581
C5	P01031	C5AR2	Q9P296	CA6	P23280
CACNA1A	O00555	CACNA1E	Q15878	CACNA1I	Q9P0X4
CAMK4	Q16566	CAMKMT	Q7Z624	CAMLG	P49069
CAPN3	P20807	CAPN5	O15484	CAPNS1	P04632, Q96L46

Input	UniProt Id	Input	UniProt Id	Input	UniProt Id
CARNS1	A5YM72	CASC3	O15234	CASP1	P29466
CASP4	P49662	CASP5	P51878	CASP7	P55210
CAT	P04040	CAV1	Q03135	CCL2	P13500
CCL20	P78556	CCL23	P55773-2	CCL8	P80098
CCNA1	P78396	CCNA2	P20248	CCR1	P32246
CCR6	P51684	CCR7	P32248	CCRL2	O00421
CD160	O95971	CD163	Q86VB7	CD1C	P29017
CD22	P20273	CD24	P25063	CD247	P20963-1
CD27	P26842	CD274	Q9NZQ7	CD300E	Q496F6
CD34	P28906-1	CD38	P28907	CD40LG	P29965
CD68	P34810	CD8B	P10966	CD96	P40200
CDC14B	O60729	CDC42EP2	O14613	CDC45	O75419
CDKN1A	P38936	CDKN1C	P49918	CEACAM1	P13688
CEP164	Q9UPV0	CEP41	Q9BYV8	CEP55	Q92753
CEP57	Q86XR8	CEP83	Q9Y592	CETP	P11597
CFB	P00751	CFP	P27918	CHM	P24386
CHMP2A	O43633	CHMP5	Q9NZZ3	CHN1	P15882
CHTF8	P0CG13	CIR1	P38117	CISH	Q9NSE2
CKAP4	Q07065	CLDN23	Q96B33	CLDN5	O00501
CLEC10A	Q6UW15, Q8IUN9	CLEC12A	Q5QGZ9	CLEC2B	Q92478
CLEC4D	Q8WXI8	CLEC4E	Q9UJ71, Q9ULY5	CLEC7A	Q9BXN2
CLOCK	O15516	CLTC	Q00610, Q00610-1	CNBP	P62633
CNOT11	Q9UKZ1	CNTRL	Q7Z7A1-3	COA6	Q5JTJ3
COG3	Q96JB2	COL13A1	Q5TAT6	COL16A1	Q07092
COL4A3	Q01955	COL5A3	P25940	COL6A2	P12110
COPE	O14579	CPNE8	Q86YQ8	CPSF6	Q16630
CRAT	P43155	CRTAP	O75718	CSAD	Q9Y600
CSTF3	Q12996	CTNNA1	P35221	CTNNBIP1	Q9NSA3
CTSF	Q9UBX1	CTSL	O60911, P07711	CUL9	Q8IWT3
CX3CR1	P49238	CXCL10	P02778	CXCL11	O14625
CXCL5	P42830, P80162	CXCL9	Q07325	CXCR2	P25024, P25025
CXCR4	P61073	CXCR6	O00574	CYBB	P04839
CYFIP2	Q96F07	CYP2E1	P05181	CYP2S1	Q96SQ9
CYP4F22	Q6NT55	CYRIB	Q9NUQ9	CYSLTR1	Q9Y271
CYTH3	O43739	DAPP1	Q9UN19	DBNL	Q9UJU6
DBP	Q03518	DCAF6	Q58WW2	DCLRE1A	Q6PJP8
DDX17	Q92841	DDX58	O95786	DENND6A	Q8IWF6
DHCR7	Q9UBM7	DHDDS	Q86SQ9	DHRS3	O75911
DHRS9	Q9BPW9	DHX58	Q96C10	DIABLO	Q9NR28
DIAPH2	O60879-2, O60879-3	DICER1	Q9UPY3	DLG5	Q8TDM6
DLGAP2	Q9P1A6	DNASE1L3	P49184	DOCK4	Q8N1I0
DOCK8	Q8NF50	DOLPP1	Q86YN1	DPYD	Q12882
DPYSL2	Q16555	DRAP1	Q14919	DSC1	Q08554
DSEL	Q8IZU8	DTX3L	Q8TDB6	DUSP5	Q16690
DUSP6	Q16828	DUT	Q6SW70	DYNC2H1	Q8NCM8
DYNLT1	P63172	E2F2	Q14209	ECT2	Q9H8V3
EDAR	Q9UNE0	EEF1A1	P68104	EEF1A1P5	Q5VTE0
EEF1B2	P24534	EEF1G	P26641	EEF2	P13639
EEPD1	Q7L9B9	EGLN1	Q9GZT9	EGR1	P18146

Input	UniProt Id	Input	UniProt Id	Input	UniProt Id
EIF2AK2	P19525	EIF3F	O00303	EIF3L	Q9Y262
EIF4B	P23588	ELOVL4	Q9GZR5	ELOVL5	Q9NYP7
ELP5	Q8TE02	ELP6	Q0PNE2	EMD	P50402
ENSA	O43768	EPB41L3	Q9Y2J2	EPHA4	P54764
EPHB2	P29323	EPHX2	P34913	EPSTI1	Q96J88
ERF	P50548	ESAM	Q96AP7	ESYT2	A0FGR8, A0FGR9
ETNK1	Q9HBU6	ETV6	P41212	EXOC1	Q9NV70
EXTL3	O43909	EZH2	Q15910	F11R	Q9Y624
FAAP20	Q6NZ36	FAM13A	O94988	FAM20A	Q96MK3
FANCA	O15360	FANCD2	Q9BXW9	FANCL	Q9NW38
FASTK	Q14296-4	FAU	P62861	FBL	P22087
FBLN2	P98095	FBXL8	Q96CD0	FBXO30	Q8TB52
FBXO6	Q9NRD1	FBXO7	Q9Y3I1	FBXO9	Q9UK97
FBXW11	Q9UKB1	FCAR	P24071	FCER1A	P12319
FCGR1A	P12314	FCGR1B	Q92637	FCGR2A	P12318
FCGR3A	P08637	FCGR3B	O75015	FCN1	O00602
FFAR2	O15552	FFAR3	O14843	FGD2	Q7Z6J4
FGD4	Q96M96	FGF9	P31371	FGL2	Q14314
FLCN	Q8NFG4	FLI1	Q01543	FLOT1	O75955
FLT3LG	P49771	FLT4	P35916	FMNL2	Q96PY5
FTH1	P02794	FXN	Q16595	GADD45B	P41440
GALNT12	Q8IXK2	GAMT	Q14353	GAPDH	P04406
GBP1	P32455	GBP2	P32456	GBP3	Q9H0R5
GBP4	Q96PP9	GBP5	Q96PP8	GBP6	Q6ZN66
GBX2	P52951	GCH1	P30793	GCHFR	P30047
GCLC	P48506	GCNT1	Q02742	GDI2	P50395
GET4	Q7L5D6	GFPT1	Q06210	GHRL	Q9UBU3-1, Q9UBU3-2
GJC2	Q5T442	GLRX	P35754	GLUL	P15104
GM2A	P17900	GNAQ	P50148	GNB4	Q9HAV0
GNG7	O60262	GNLY	P22749	GOLGA7	Q7Z5G4
GORASP1	Q9BQQ3	GPBAR1	Q8TDU6	GPC6	Q9Y625
GPD2	P43304	GPR183	P32249	GPR65	Q8IYL9
GPR68	Q15743	GPR84	Q9NQS5	GRAP	Q13588
GRB14	Q14449	GRIN3A	Q8TCU5	GRN	P28799
GSTM2	P28161	GTPBP1	Q9BX10	GTPBP2	Q9BX10
GYG1	P46976	GZMM	P51124	H1-2	P16403
H2BC12	O60814, Q99877	H2BC18	Q5QNW6	H2BC4	P62807, Q93079
H2BC5	P58876	H3-3A	P84243	H3C4	P68431, Q71DI3
H4C8	P62805	HADHA	P40939	HAS3	O00219
HBEGF	Q99075	HBG1	P69891	HBG2	P69892
HDAC5	Q9UQL6	HELZ2	Q9BYK8	HERC4	Q15034, Q5GLZ8
HERC5	Q9UII4	HERC6	Q8IVU3	HERPUD1	Q15011
HGD	Q93099	HK3	P52790	HLA-A	P04439
HLA-B	P01889	HLA-C	P10321	HLA-DQA1	P01909, P20036
HLA-DQA2	P01906	HLA-DRA	P01903	HLA-DRB4	P13762
HLA-G	P17693	HLA-H	P01893	HMBS	P08397
HMGN2	P05114	HNMT	P50135	HNRNPA2B1	P22626
HOMER2	Q9NSB8	HP	P00738	HPS4	Q9NQG7
HSD17B13	Q7Z5P4	HSH2D	Q9NP31	HSPA1A	P0DMV8

Input	UniProt Id	Input	UniProt Id	Input	UniProt Id
HSPA6	P17066	HSPA7	P48741	ICE1	Q9Y2F5
ICOSLG	O75144	ID2	Q02363	ID3	Q02535
IDO1	P14902, Q6ZQW0	IFI16	Q16666	IFI27	P40305
IFI30	P13284	IFI35	P80217	IFI6	P09912
IFIH1	Q9BYX4	IFIT1	P09914	IFIT2	P09913
IFIT3	O14879	IFIT5	Q13325	IFITM1	P13164
IFITM2	Q01629	IFITM3	P13164, Q01628	IFNG	P01579
IFT122	Q9HBG6	IFT140	Q96RY7	IGF1R	P08069
IGF2BP3	O00425	IGHA1	P01876	IGHE	P01854
IGHG1	P01857, P01861	IGHG3	P01860	IGHG4	P01861
IGHV1-69	P01742	IGHV3-11	P01762	IGHV3-23	P01764
IGHV3-30	P01768	IGHV3-33	P01772	IGHV4-34	P06331
IGHV4-39	P01824	IGHV4-59	P01825	IGKC	P01834
IGKV1-12	A0A0C4DH73	IGKV1-17	P01599	IGKV1-33	P01594
IGKV1-5	P01602	IGKV1D-39	P04432	IGKV3-11	P04433
IGKV3-15	P01624	IGKV3-20	P01619	IGLC1	P0CG04
IGLC2	P0DOY2	IGLC3	P0DOY3	IGLC7	A0M8Q6
IGLV1-40	P01703, Q5NV69	IGLV1-44	P01699, Q5NV81	IGLV1-47	P01700
IGLV1-51	P01701	IGLV2-14	P01704	IGLV2-23	P01705, Q5NV89
IGLV3-1	P01715	IGLV3-19	P01714	IGLV3-21	P80748
IGLV3-25	P01717, Q5NV90	IGLV6-57	P01721	IKBIP	Q70UQ0
IL11RA	P08887, Q14626	IL15	P40933	IL1RN	P18510
IL27	Q8NEV9	IL2RB	P14784	IL37	Q9NZH6
IL4R	P24394, Q01113	IL5RA	P15509, Q01344	IL6	P05231
IL6R	P08887, P08887-2	IL7R	P16871	IMPA2	O14732
IMPDH1	P20839	ING5	Q8WYH8	INPP5E	Q10713
INTS7	Q9NVH2	IP6K1	Q92551	IP6K2	Q9UHH9
IQGAP1	P46940	IRF1	P10914	IRF5	Q13568
IRF7	Q92985	IRF9	Q00978	ISG15	P05161
ISG20	Q96AZ6	ITGA4	P13612	ITGA6	P23229
ITGB1	P05556	ITGB4	P16144	JADE1	Q6IE81
JAK2	O60674	JUP	P14923	KAT8	Q9H7Z6
KCNH3	Q9ULD8	KCNJ15	Q99712	KCNQ3	O43525
KDM1B	Q8NB78	KDM4B	O94953	KHK	P50053
KIR2DL1	P43626, P43629	KIR3DL1	P43629	KISS1R	Q969F8
KIT	P10721	KLF4	O43474	KLHL3	Q9UH77
KLRB1	Q12918	KLRF1	Q9NZS2	KLRG1	Q96E93
KMO	O15229	KMT5B	Q4FZB7, Q86Y97	KPNB1	Q14974
KPTN	Q9Y664	KRT1	P04264	KRT5	P13647
KRT72	Q14CN4	KRT73	Q86Y46	KYNU	Q16719
LAG3	P18627	LAMP3	P34810	LAMTOR1	Q6IAA8
LAP3	Q13867	LDLRAP1	Q5SW96	LEF1	Q9UJU2
LGALS3BP	Q08380	LGALS9	O00182	LGMN	Q99538
LHFPL2	Q6ZUX7	LIF	P15018	LILRA4	P59901
LILRA5	A6NI73, Q8N149	LILRA6	Q6PI73	LILRB1	Q8NHL6
LILRB2	Q8N423	LILRB4	Q8N149, Q8NHJ6	LIPN	Q5VXI9
LMNB1	P20700	LMO2	P25791	LPAR6	P43657
LRFN3	Q9BTN0	LRFN4	Q6PJG9	LRRK2	Q5S007
LRTOMT	Q8WZ04	LTB	Q06643	LTBP3	Q14767, Q9NS15

Input	UniProt Id	Input	UniProt Id	Input	UniProt Id
LY6E	Q16553	LY96	Q9Y6Y9	LYPLA2	O95372
MAD1L1	Q9Y6D9	MAF	O75444	MAFB	Q9Y5Q3
MAGED1	Q9Y5V3	MAGED2	Q9UNF1	MAGOH	P61326
MAL	P01732, P58753	MAP2K3	P46734	MAP2K6	P52564
MAP3K1	Q13233	MAPK3	P27361	MARCKS	P29966
MASTL	Q96GX5	MATK	P42679	MAX	P61244
MBP	P13727	MCAT	Q8IVS2	MCM8	Q9UJA3
MCOLN3	Q8TDD5	MCRS1	Q96EZ8	MED16	Q9Y2X0
MED25	Q71SY5, Q9NWA0	MEF2C	Q06413	MEFV	O15553
MERTK	Q12866	METTL1	Q9UBP6	METTL21A	Q8WXB1
MFGE8	Q08431	MICAL1	Q8TDZ2	MICB	Q29980
MIS18BP1	Q6P0N0	MLKL	Q8NB16	MMP8	P22894
MNDA	P41218	MOV10	Q9HCE1	MPST	P25325
MRPL24	Q96A35	MRPL45	Q9BRJ2	MSR1	P21757
MSRA	Q9UJ68	MT1A	P02795	MT1E	P04732
MT1F	P04733	MT2A	P02795	MTHFD2	P13995
MTMR12	Q9C0I1	MTX1	Q13505	MUC1	P15941
MUC6	Q6W4X9	MX1	P20591	MX2	P20592
MYBL1	P10243	MYBL2	P10244	MYD88	Q99836
NADK	O95544	NAGK	Q9UJ70	NAMPT	P43490
NAPA	Q9Y2A7	NBN	O60934	NCALD	P61601
NCAM1	P13591	NCAPG	Q9BPX3	NCF1	P14598
NCF2	P19878	NCOA1	Q15788	NCOA3	Q9Y6Q9
NCR3	O14931	NDC80	O14777	NDEL1	Q9GZM8
NDUFA10	O95299	NDUFA3	O95167	NDUFAF1	Q9Y375
NDUFAF5	Q5TEU4	NDUFAF6	Q330K2	NDUFAF7	Q7L592
NDUFB4	O95168	NELL2	Q99435	NEO1	Q92859
NF1	P21359	NFIL3	Q16649	NHLRC3	Q5JS37
NLRC5	Q86WI3	NLRX1	Q86UT6	NMB	Q14956
NMI	Q13287	NMNAT3	Q96T66	NMUR1	O94822
NNMT	P40261	NOC4L	Q9BVI4	NOD2	Q9HC29
NOG	Q13253	NOSIP	Q9Y314	NPAS2	Q99743
NPC2	P61916	NPDC1	Q9NQX5	NR1D1	P20393
NR3C2	P08235	NRCAM	Q92823	NRIP1	P48552
NSD3	Q9BZ95	NT5C3A	Q9H0P0-4	NT5E	P21589
NTNG2	Q96CW9	NUB1	Q9Y5A7-1, Q9Y5A7-2	NUDT5	Q9UKK9
NUP188	Q5SRE5	OAS1	P00973	OAS2	P29728
OAS3	Q9Y6K5	OASL	Q15646	OAZ1	P54368
OGG1	O15527, O15527-4	OR2AG1	Q9H205	OSBPL6	Q9BZF3
OTOF	Q9HC10	P2RX1	P51575	P2RY13	Q9BPV8
P3H2	Q8IVL5	PABPC1	P11940	PACSIN1	Q9BY11
PAFAH1B3	Q15102	PANX2	Q96RD6	PARD6A	Q9NPB6
PARP10	Q53GL7	PARP14	Q460N5	PARP16	Q8N5Y8
PARP9	Q8IXQ6	PATL1	Q86TB9	PCGF5	Q86SE9
PCK2	Q16822	PCSK5	Q92824	PDCD1LG2	Q9BQ51
PDGFD	Q9GZP0	PDGFRB	P09619	PDK4	Q16654
PDLIM7	Q9NR12	PDPK1	O15530	PDSS1	Q5T2R2
PEX16	Q9Y5Y5	PFKP	Q01813	PFN2	P07737, P35080
PGAM1	P18669	PGAP1	Q75T13	PHC3	Q8NDX5

Input	UniProt Id	Input	UniProt Id	Input	UniProt Id
PHF1	O43189	PHF19	Q5T6S3	PHF20L1	A8MW92
PHGDH	O43175	PHYH	O14832	PI3	P19957
PI4K2B	Q8TCG2	PIGP	P57054	PIGQ	Q9BRB3-2
PIK3AP1	Q6ZUJ8	PIK3CA	P42336	PILRA	Q9UKJ1
PIN1	Q13526	PIWIL4	Q7Z3Z4	PJA1	Q8NG27
PKD2	Q13563	PLAAT4	P53816, Q9UL19	PLAUR	Q03405
PLEKHA1	Q9HB21	PLEKHG1	Q9ULL1	PLOD1	Q02809
PLPP5	Q8NEB5	PML	P29590	PMM2	O15305
PNPT1	Q8TCS8	POLQ	O75417	POLR1E	O15160
POLR3H	Q9Y535	POTEKP	Q9BYX7	PPARGC1B	Q86YN6
PPBP	P02775	PPIA	P62937	PPP2R2D	Q66LE6
PPP2R5D	Q14738	PPP3CA	Q08209	PPP3CB	P16298
PPP3CC	P16298	PPP3R1	P63098	PRAG1	Q86YV5
PRC1	O43663	PRKCB	P05771	PRKCD	Q05655
PRKCE	Q02156	PRKCSH	P14314	PRKDC	P78527
PRPF4B	Q13523	PRR5	P85299	PSMB8	P28062
PSMB9	P28065	PSME1	Q06323	PSME2	Q9UL46
PTCH1	Q13635	PTGDR	Q13258	PTGDS	P41222
PTGES3	Q15185	PTGFR	P43088	PTK2B	Q14289
PTPN2	P17706-2	PTPN22	Q9Y2R2	PTPRF	P10586
PTPRN2	Q92932	PTPRO	Q92729	PTPRS	P10586, Q13332
QTRT1	Q9BXR0	RAB24	Q969Q5	RAB33A	Q14088
RAB3D	O95716	RAB43	Q86YS6	RAD21	O60216
RAD51AP1	Q96B01	RASGRF2	O14827	RBBP8	Q99708
RBCK1	Q9BYM8	RCAN3	Q9UKA8	RDX	P35241
REC8	O95072	REG4	Q06141	REL	Q01201
RGCC	Q9H4X1	RGL1	Q9NZL6	RHBDF2	Q6PJF5
RHOBTB3	O94955	RHOT1	Q8IXI2	RIGI	O95786
RIN2	Q8WYP3	RLIM	Q9NVW2	RMI2	Q96E14
RNASE2	P10153	RNF213	Q63HN8	RNF31	Q96EP0
RNF38	Q9P000	RORA	P35398	RORC	P51449
RPA1	P27694	RPL10	P27635, Q96L21	RPL10A	P61313, P62906
RPL13	P26373, P40429	RPL13A	P40429, P61313	RPL15	P61313
RPL18	Q07020	RPL21	P46778	RPL27A	P46776, P61353
RPL29	P47914	RPL3	P39023, Q92901	RPL30	P62888
RPL31	P62899	RPL32	P62888, P62910	RPL34	P49207, P62899
RPL35	P42766	RPL35A	P18077	RPL36	Q9Y3U8
RPL38	P63173	RPL3L	Q92901	RPL4	P36578
RPL5	P46777	RPL7	P18124	RPL7A	P18124, P62424
RPL9P8	P32969	RPLP0	P05388	RPLP2	P05387
RPN2	P04844	RPS10	P46783	RPS11	P62280
RPS12	P25398	RPS13	P62277	RPS14	P62263
RPS15	P62841	RPS15A	P62244	RPS16	P62249
RPS17	P08708	RPS18	P62269	RPS2	P15880, P46782
RPS23	P62266	RPS27	P42677, Q71UM5	RPS27A	P42677, P62979, P62987
RPS29	P62273	RPS3	P23396	RPS3A	P61247
RPS4X	P62701, Q8TD47	RPS5	P46782	RPS6	P62753
RPS8	P62241	RPS9	P46781	RPSA	P08865
RRAS	P10301	RRM2	P31350	RSAD2	Q8WXG1

Input	UniProt Id	Input	UniProt Id	Input	UniProt Id
RSF1	Q96T23	RTN3	O95197	RTN4	Q9NQC3
RTN4R	Q9BZR6	S100B	P04271	S1PR5	Q9H228
SAP30	O75446, Q9HAJ7	SAT1	Q9H2H9	SCARB2	Q14108
SCO2	P78563	SDC3	O75056	SDCBP	O00560
SDK2	Q58EX2	SEC24D	O94855	SEC61G	P60059
SELENOI	Q9C0D9	SELL	P14151	SEPTIN4	O43236-6
SERPINE2	P07093	SERPING1	P05155	SERPINH1	P50454
SESN1	Q9Y6P5	SGF29	Q96ES7	SH2D1B	O14796
SHH	Q15465	SHISA5	Q8N114	SIGIRR	Q6IA17
SIGLEC1	Q9BZZ2	SIGLEC14	Q08ET2	SIGLEC5	O15389, O43699
SIGLEC7	Q9Y286	SIGLEC8	Q96PQ1, Q9NYZ4	SIK1	O00567
SIRPA	P78324	SIRPG	Q9P1W8	SIT1	Q9NP91
SKAP2	O75563	SLC15A2	Q16348	SLC15A4	Q8N697
SLC16A10	Q8TF71	SLC1A3	P43003	SLC22A23	Q3SY84
SLC24A4	Q8NFF2	SLC25A1	P53007	SLC25A6	P12236
SLC27A1	Q6PCB7	SLC27A3	O14975	SLC30A1	Q9Y6M5
SLC35B3	Q9H1N7	SLC39A3	Q9BRY0	SLC4A1	P02730
SLC4A10	Q6U841	SLC6A12	P48065	SLC7A7	Q9UM01
SLC7A8	Q9UHI5	SMG7	Q92540	SMG9	Q9H0W8
SMPD1	P17405	SMPD3	Q9NY59	SNRPN	P63162
SNU13	P55769	SOCS1	O15524	SOCS2	O14508
SOCS3	O14543	SORBS3	O60504	SORT1	Q99523
SOS2	P42766	SP100	P23497	SP110	P23497
SPON2	Q9BUD6	SPRED1	Q7Z699	SPSB2	Q99619
SPTLC2	O15270	SQLE	Q14534	SQOR	Q9Y6N5
SQSTM1	Q13501	SRGAP2	O43295, O75044	ST3GAL3	Q11203
ST3GAL4	Q11206	ST7	Q9Y561	STARD10	Q9Y365
STARD8	Q92502	STAT1	P42224-1	STAT2	P52630
SYK	P43405	TAB3	Q8N5C8	TAGLN2	P37802
TAP1	Q03518	TAP2	Q03519	TARBP2	Q15633
TAX1BP3	O14907	TBL1X	O60907	TBL3	Q12788
TCF7	P36402	TCF7L2	Q9NQB0	TCN1	P20061
TCN2	P20062	TCTN3	Q6NUS6	TEAD4	Q15561, Q15562
TECR	Q5VVJ2	THBD	P07204	THRA	P10827-2
TIA1	P31483	TIFA	Q96CG3	TIMELESS	Q9UNS1
TIMM10	P62072	TIMP2	P16035	TKTL1	P29401
TLE2	Q04725	TLR1	Q15399	TLR2	O60603
TLR4	O00206	TLR5	O60602	TLR7	Q9NYK1
TLR8	Q9NR97	TLR9	Q9NR96	TMED7	Q9Y3B3
TMOD2	Q9NZR1	TMX3	Q96JJ7	TNFAIP6	P98066
TNFRSF10B	O14763, Q9UBN6	TNFRSF17	Q02223	TNFRSF21	O75509
TNFRSF25	Q93038	TNFSF10	P50591	TNFSF13	O75888
TNFSF13B	Q9Y275	TNFSF14	O43557	TNNC2	P02585
TNRC6A	Q8NDV7	TOM1L2	O60784	TOMM6	Q96B49
TOMM7	Q9P0U1	TOP2A	P11388	TOR1B	O14657
TOR4A	Q9NXH8	TPM1	P06753	TPM2	P07951
TPX2	Q9ULW0	TRAF3	Q13114	TREML1	Q86YW5
TREX1	Q9NSU2	TRIM14	Q14142	TRIM21	P19474
TRIM22	Q8IYM9	TRIM25	Q14258	TRIM27	P14373

Input	UniProt Id	Input	UniProt Id	Input	UniProt Id
TRIM34	Q9BYJ4	TRIM38	O00635	TRIM5	Q9C035
TRIM56	Q9BRZ2	TRIM6	Q9C030	TRIM69	Q86WT6
TRIP12	Q14669	TRMT61B	Q9BVS5	TSC22D3	Q99576
TSPAN33	Q86UF1	TSPO	P30536	TSPOAP1	O95153
TST	Q16762	TTC26	A0AVF1	TTLL4	Q14679
TTYH3	Q9C0H2	TUBA4A	P68366	TXK	P42681
TXNDC5	Q8NBS9	TYMP	P19971	TYMS	P04818
U2AF1L4	Q8WU68	UBA6	A0AVT1	UBA7	P41226
UBE2C	O00762	UBE2G1	P62253	UBE2J2	Q8N2K1
UBE2L6	O14933	UBOX5	O94941	UBXN1	Q04323
UFD1	Q92890	UFL1	O94874	UHMK1	Q8TAS1
UHRF2	Q96PU4	UNC93B1	Q9H1C4	UPF3B	Q9BZI7
URM1	Q9BTM9	USP1	O94782	USP15	Q9Y4E8
USP18	Q9UMW8	USP25	Q9UHP3	USP41	Q3LFD5
USP8	P40818	UXT	Q9UBK9	VCAN	P13611
VCPIP1	Q96JH7	VEGFB	P49765	VIM	P08670
VPS51	Q9UID3	VRK2	Q86Y07	VRK3	Q8IV63
WARS1	P23381	WIPF1	O43516	WLS	Q5T9L3
WNT16	Q9UBV4	WNT2B	Q93097	WSB1	Q9Y6I7
XAF1	Q6GPH4	XCL2	Q9UBD3	XPO1	O14980
XRN1	Q8IZH2	ZBP1	Q9H171	ZC3HAV1	Q7Z2W4
ZDHHC9	Q9Y397	ZEB2	O60315	ZFYVE9	O95405-1
ZNF2	Q9BSG1	ZNF333	Q96JL9	ZNF446	Q9NWS9
ZNF451	Q9H7R0	ZNF467	Q7Z7K2	ZNF484	Q5JVG2, Q8N8J6
ZNF496	Q96IT1	ZNF586	Q9NXT0	ZNF595	Q8IYB9
ZNF600	Q6ZNG1	ZNF668	Q96K58	ZNF684	Q5T5D7
ZNF74	Q16587				

Input	Ensembl Id	Input	Ensembl Id	Input	Ensembl Id
ABCA1	ENSG00000165029, ENST00000374736.7	ABCG1	ENSG00000160179	ACSL1	ENSG00000151726
ACTA2	ENSG00000107796	ADAR	ENSG00000160710	ALOX15	ENSG00000161905
APOD	ENSG00000189058	ARF1	ENSG00000143761	ATF3	ENSG00000162772
AXIN2	ENSG00000168646	B2M	ENSG00000166710	BCL2A1	ENSG00000140379
BCL2L14	ENSG00000121380	BRCA1	ENSG00000012048	BST2	ENSG00000130303
CASP1	ENSG00000137752	CAT	ENSG00000121691	CAV1	ENSG00000105974
CCL2	ENSG00000108691	CCL20	ENSG00000115009	CCNA1	ENSG00000133101
CCNA2	ENSG00000145386	CCR1	ENSG00000163823	CD163	ENSG00000177575
CD274	ENSG00000120217	CDC45	ENSG00000093009	CDKN1A	ENSG00000124762
CETP	ENSG00000087237	CISH	ENSG00000114737	CLDN5	ENSG00000184113
CLOCK	ENSG00000134852	CXCL10	ENSG00000169245	CXCR4	ENSG00000121966
DBP	ENSG00000105516	DHCR7	ENSG00000172893	EEPD1	ENSG00000122547
EGR1	ENSG00000120738	EIF2AK2	ENSG00000055332	EPHA4	ENSG00000116106
EXTL3	ENSG00000012232	EZH2	ENSG00000106462	FANCD2	ENSG00000144554, ENST00000419585
FCGR1A	ENSG00000150337	FCGR1B	ENSG00000198019	FGF9	ENSG00000102678
FLT4	ENSG00000037280	FTH1	ENST00000273550	GAMT	ENSG00000130005
GBP1	ENSG00000117228	GBP2	ENSG00000162645	GBP3	ENSG00000117226
GBP4	ENSG00000162654	GBP5	ENSG00000154451	GBP6	ENSG00000183347
GBX2	ENSG00000168505	GCLC	ENSG00000001084	GFPT1	ENSG00000198380
GHRL	ENSG00000157017	HBG1	ENSG00000213934	HBG2	ENSG00000196565

Input	Ensembl Id	Input	Ensembl Id	Input	Ensembl Id
HERPUD1	ENSG00000051108	HLA-A	ENSG00000206503	HLA-B	ENSG00000234745
HLA-C	ENSG00000204525	HLA-DQA1	ENSG00000196735	HLA-DQA2	ENSG00000237541
HLA-DRA	ENSG00000204287	HLA-DRB4	ENSG00000227357	HLA-G	ENSG00000204632
HLA-H	ENSG00000206341	HNRNPA2B1	ENSG00000122566	HSPA1A	ENSG00000204389, ENSG00000215328, ENSG00000234475, ENSG00000235941, ENSG00000237724
HSPA6	ENSG00000173110	ID2	ENSG00000115738	ID3	ENSG00000117318
IFI27	ENSG00000165949	IFI30	ENSG00000216490	IFI35	ENSG00000068079
IFI6	ENSG00000126709	IFIT1	ENSG00000185745	IFIT2	ENSG00000119922
IFIT3	ENSG00000119917	IFIT5	ENSG00000152778	IFITM1	ENSG00000185885
IFITM2	ENSG00000185201	IFITM3	ENSG00000142089	IFNG	ENSG00000111537
IGHE	ENSG00000211891	IGHG1	ENSG00000211896	IGHG4	ENSG00000211892
IKBIP	ENSG00000166130	IL1RN	ENSG00000136689	IL4R	ENSG00000077238
IL6	ENSG00000136244	IL6R	ENSG00000160712	IP6K2	ENSG00000068745
IRF1	ENSG00000125347	IRF5	ENSG00000128604	IRF7	ENSG00000185507
IRF9	ENSG00000213928	ISG15	ENSG00000187608	ISG20	ENSG00000172183
ITGA4	ENSG00000115232	ITGB1	ENSG00000150093	KDM1B	ENST00000297792
KDM4B	ENST00000381759	KIT	ENSG00000157404	KLF4	ENSG00000136826
LIF	ENSG00000128342	LMNB1	ENSG00000113368	MEF2C	ENSG00000081189
MT2A	ENSG00000125148	MUC1	ENSG00000185499	MX1	ENSG00000157601
MX2	ENSG00000183486	MYBL2	ENSG00000101057	NAMPT	ENSG00000105835
NCAM1	ENSG00000149294	NPAS2	ENSG00000170485	NR1D1	ENSG00000126368
OAS1	ENSG00000089127	OAS2	ENSG00000111335	OAS3	ENSG00000111331
OASL	ENSG00000135114	OR2AG1	ENSG00000279486	PML	ENSG00000140464
PPIA	ENSG00000196262	PRKCB	ENSG00000166501	PSMB8	ENSG00000204264
PSME2	ENSG00000100911	PTCH1	ENSG00000185920	PTGDS	ENSG00000107317
PTPN2	ENSG00000175354	RBBP8	ENSG00000101773	RGCC	ENSG00000102760
RGL1	ENSG00000143344	RORA	ENSG00000069667	RORC	ENSG00000143365
RPLP0	ENSG00000089157	RPS2	ENST00000343262	RRM2	ENSG00000171848
RSAD2	ENSG00000134321	S100B	ENSG00000160307	SCO2	ENSG00000130489
SERPINH1	ENSG00000149257	SESN1	ENSG00000080546	SHH	ENSG00000164690
SLC27A1	ENSG00000130304	SOCS1	ENSG00000185338	SOCS2	ENSG00000120833
SOCS3	ENSG00000184557, ENST00000330871	SP100	ENSG00000067066	SQLE	ENSG00000104549
SQSTM1	ENSG00000161011	STAT1	ENSG00000115415	TNFRSF10B	ENSG00000120889
TNFRSF21	ENSG00000146072	TOP2A	ENSG00000131747	TRIM14	ENSG00000106785
TRIM21	ENSG00000132109	TRIM22	ENSG00000132274	TRIM25	ENSG00000121060
TRIM34	ENSG00000258659	TRIM38	ENSG00000112343	TRIM5	ENSG00000132256
TRIM6	ENSG00000121236	TYMS	ENSG00000176890	UXT	ENSG00000126756
VIM	ENSG00000026025	XAF1	ENSG00000132530	ZEB2	ENSG00000169554

Input	ChEBI Id
MAL	15589

7. Identifiers not found

These 576 identifiers were not found neither mapped to any entity in Reactome.

ABHD14A	ABTB3	ADAP2	ADGRE4P	ADGRL1	AFF1	AFG3L1P	AFTPH
AGAP1	AHNAK	AHSA2P	AIDA	AIG1	AIRE	ALKBH7	AMIGO1
AMN1	ANKRD17	ANKRD22	ANKRD23	ANXA2P1	ANXA2R	ANXA3	ANXA4
AP5B1	APOL2	APOL4	APOL6	ARL5B	ARMCX6	ARMT1	ASCL2
ASF1B	ASMTL	ASPM	ASPRV1	ATP6V0E2-AS1	ATRN	AZI2	BAIAP3
BATF2	BAZ1A	BAZ2B	BCAR3	BCL11B	BCL2L2	BCL7B	BOK
BRSK1	BSCL2	BTBD3	BTN2A3P	C10orf105	C12orf4	C12orf57	C14orf132
C15orf48	C17orf58	C1orf198	C21orf91	C3orf14	C5orf24	C8orf31	C9orf72
CA11	CACHD1	CALHM6	CAMK2N1	CAND2	CAPRIN1	CARD16	CARD17
CARD17P	CARD6	CASP4LP	CCDC124	CCDC146	CCDC17	CCDC90B	CCDC96
CCL18	CCM2	CCNJL	CCNY	CD248	CD6	CD69	CD7
CD82	CDCA7	CDHR1	CDK20	CDR2L	CENPBD2P	CENPV	CEP85L
CFAP410	CFAP44	CFAP58	CFAP58-DT	CHRM3-AS2	CIDECP1	CKAP2	CKS2
CLIC3	CLIC4	CLK4	CLPP	CMAHP	CMPK2	CMTR1	CNIH4
CNP	CNTNAP3	COCH	COLQ	CPNE2	CPQ	CRIP2	CSRNP1
CTDSPL	CTRL	CUTA	CUX2	CXCR2P1	CXorf21	CXorf65	Calcineurin
DALRD3	DBNDD1	DCANP1	DCBLD1	DDX60	DDX60L	DEAF1	DEXI
DHRS11	DIPK2A	DISC1	DLEU2L	DMTF1	DMXL2	DNAH6	DNAJB12
DNAJB14	DNAJB5	DONSON	DPCD	DUSP22	EEF1A1P6	EEF1GP1	EEIG2
EFCAB12	ELAPOR1	EMC9	ENKD1	EPB41L4A-AS1	EPB42	ERGIC3	ETV5
ETV7	EVA1B	EVA1C	EWSR1	EXOC3L1	EXOG	F8A1	FAM102A
FAM153B	FAM177A1	FAM177B	FAM209B	FAM225A	FAM53C	FAM72B	FAM76B
FAM8A1	FASTKD1	FAT4	FAXDC2	FBXO39	FCGBP	FCGR1CP	FCGR2C
FCRL5	FEZ1	FLVCR2	FNDC3A	FRMD3	FTH1P3	GALM	GASK1B
GATAD1	GBP1P1	GCNT2	GFOD2	GLRX2	GOLGA2P10	GOLGA6L1	GOLGA8B
GORAB	GPA33	GPATCH2	GPBP1	GPR162	GPRASP1	GRAMD1B	GRIPAP1
GTF2IP1	GUCD1	HDGFL2	HES4	HESX1	HLA-DRB6	HLA-J	HLA-K
HMGB1P3	HOPX	HOXB7	HSPBP1	IFI44	IFI44L	IGHGP	IGHV1-18
IGHV3-15	IGHV3-21	IGHV4-61	IGHV5-51	IGKV1-27	IGKV3D-15	IGLL5	IKZF4
INKA2	IRF1-AS1	IRX3	ITIH5	ITPRIPL2	Immunoglobulin	JPX	KATNAL1
KBTBD4	KIAA0319L	KIAA0895L	KIAA1109	KIAA1958	KLHDC7B	KLHDC8B	KLRC3
KRT10-AS1	KRTCAP2	L-tryptophan	LACTB	LAMP5	LBH	LEF1-AS1	LENG8
LGALS9B	LINC00487	LINC00857	LINC00937	LINC00999	LINC01002	LINC01061	LMO4
LRMDA	LRRC15	LRRC23	LRRC26	LRRC37A4P	LRRC56	LRRN3	LTK
LY9	LYRM9	LYSMD2	MACROD2	MAEA	MANSC1	MAP3K7CL	MAP4K1
MAP7	MARCHF1	MARCHF5	MAST2	MCF2L-AS1	MCRIP2	MELK	MICAL2
MID2	MKI67	MMP28	MOB3A	MOB3B	MOK	MORC2-AS1	MOXD1
MPZL1	MPZL2	MS4A1	MS4A4A	MS4A7	MTPN	MTRF1	MTSS1
MTURN	MTUS1	MUSTN1	MXI1	MYO7B	MYOF	MZB1	N4BP2L2
NAIP	NAP1L3	NBDY	NCOA7	NDRG2	NELL1	NEXN	NFE2L3
NIPSNAP3A	NLRP2	NLRP7	NOP53	NPTXR	NSG1	NUDT16L2P	NUFIP2
NUSAP1	ODAD4	ODF3B	ONECUT2	OR52K3P	OST4	OTUD6B	PABPC4
PARP12	PARP15	PARP3	PATL2	PAWR	PCDHGB6	PDCD2L	PDIA3P1
PDXP	PDZD4	PDZK1IP1	PGAM4	PGAP3	PGAP6	PHACTR1	PHF11
PHLDB1	PHLDB2	PIK3IP1	PITPNC1	PLEKHB1	PLLP	PLSCR1	PLXDC1
POGLUT3	POLR2J2	PPHLN1	PPM1H	PPP1R32	PPP3R2	PRAF2	PRF1

PRKCQ-AS1	PRORSD1P	PRRG4	PRRT2	PRSS30P	PRSS33	PRXL2A	PRXL2C
PSME2P2	PSTPIP2	PTBP2	PTGES3P1	PTMA	PTP4A1	R3HCC1	RABGAP1L
RAD54B	RASD1	RASGEF1B	RASSF3	RBM15B	RBM45	RBPMS2	RECQL
REEP6	REM2	RETREG1	RFLNB	RFX2	RGPD1	RIMS3	RN7SL1
RNF126P1	RNF148	RNF5P1	ROPN1B	RPH3A	RPL10P3	RPL13AP5	RPL3P4
RPL7P1	RPL7P9	RPP25L	RPS4XP22	RPSAP9	RSPH9	RTN2	RTP4
RUBCN	RUFY3	RUFY4	RWDD4	SALL2	SAMD14	SAMD4A	SAMD9
SAMD9L	SASH1	SASS6	SCAMP5	SCARNA9	SCFD2	SCML1	SCYL1
SDR39U1	SEC14L1	SECTM1	SELENBP1	SEMA4C	SERPINF1	SESTD1	SGTB
SH3TC1	SH3YL1	SHFL	SIGLEC17P	SIPA1L2	SLC25A39	SLC26A8	SLC41A3
SLF1	SLFN11	SLFN12	SMCHD1	SMCO4	SNHG29	SNHG32	SNHG5
SNORA32	SNORD32A	SNTA1	SNX10	SP140	SPATS2L	SPG11	SPG21
SPIN4	SPINK2	SPNS3	SPOCK2	SPRYD3	SRBD1	SRFBP1	SRGAP2B
SRGAP2C	SRRD	SS18	SSH3	STAC3	STIL	STK16	STMN3
STX11	SULF2	SUSD1	SYTL2	TAGLN	TARDBP	TARP	TASL
TBC1D8	TCEA3	TCP11L1	TDRD7	TENT5A	TFEC	TGFB1I1	TGM2
TGM3	THNSL1	TIGIT	TIPRL	TLCD3A	TMEM123	TMEM127	TMEM140
TMEM144	TMEM176A	TMEM176B	TMEM178B	TMEM191A	TMEM191C	TMEM203	TMEM204
TMEM207	TMEM258	TMEM259	TMEM268	TMEM62	TMEM87B	TMEM8B	TMEM97
TMIGD2	TMPRSS15	TMTC1	TMX4	TOP1MT	TOR2A	TP53INP2	TPRA1
TPT1	TRABD2A	TRAFD1	TRANK1	TRAV1-2	TRDC	TRDV2	TRGC1
TRGV9	TRIM6-TRIM34	TSPAN18	TTC21A	TTC39C	TXNDC12	UBE2Q2P6	UBL7
UBQLNL	VAMP5	VNN3	VOPP1	VPS50	VWCE	WDFY1	WDR5B
WDR73	XKR8	XKRX	XPO7	YBX3P1	YIF1B	YTHDC2	ZCCHC2
ZFC3H1	ZFP57	ZNF117	ZNF174	ZNF185	ZNF358	ZNF438	ZNF575
ZNF674	ZNF814	ZNFX1	ZNRD2	ZSCAN16	ZSCAN18	ZUP1	kynurenine
